# Multi-Technique Investigation of Grave Robes from 17th and 18th Century Crypts Using Combined Spectroscopic, Spectrometric Techniques, and New-Generation Sequencing

**DOI:** 10.3390/ma14133535

**Published:** 2021-06-24

**Authors:** Magdalena Śliwka-Kaszyńska, Marek Ślebioda, Anna Brillowska-Dąbrowska, Martyna Mroczyńska, Jakub Karczewski, Anna Marzec, Przemysław Rybiński, Anna Drążkowska

**Affiliations:** 1 Department of Organic Chemistry, Faculty of Chemistry, Gdańsk University of Technology (Gdańsk Tech), 80-233 Gdańsk, Poland; 2 Perlan Technologies, Sp. z.o.o., 02-785 Warszawa, Poland; m.slebioda@wp.pl; 3 Department of Molecular Biotechnology and Microbiology, Faculty of Chemistry, Gdańsk University of Technology (Gdańsk Tech), 80-233 Gdańsk, Poland; annbrill@pg.edu.pl (A.B.-D.); marmroc1@pg.edu.pl (M.M.); 4 Faculty of Applied Physics and Mathematics, Gdańsk University of Technology (Gdańsk Tech), 80-233 Gdańsk, Poland; jkarczew@mif.pg.gda.pl; 5 Institute of Polymer and Dye Technology, Faculty of Chemistry, Lodz University of Technology, 90-924 Lodz, Poland; anna.marzec@p.lodz.pl; 6 Institute of Chemistry, Faculty of Natural Science, The Jan Kochanowski University, 25-369 Kielce, Poland; przemyslaw.rybinski@ujk.edu.pl; 7 Faculty of History, Nicolaus Copernicus University in Torun, 87-100 Torun, Poland; annadr@umk.pl

**Keywords:** natural dyes, mordants, tandem mass spectrometry, grave robes, next-generation sequencing

## Abstract

The textile fragments of the funeral clothes found in the 17th and 18th century crypts were subjected to spectroscopic, spectrometric, and microbial investigation. The next-generation sequencing enabled DNA identification of microorganisms at the genus and in five cases to the species level. The soft hydrofluoric acid extraction method was optimized to isolate different classes of dyes from samples that had direct contact with human remains. High-performance liquid chromatography coupled with diode matrix and tandem mass spectrometry detectors with electrospray ionization (HPLC-DAD-ESI-MS/MS) enabled the detection and identification of 34 colourants that are present in historical textiles. Some of them are thus far unknown and uncommon dyes. Indigo, madder, cochineal, turmeric, tannin-producing plant, and young fustic were identified as sources of dyes in textiles. Scanning electron microscopy with energy-dispersive X-ray detector (SEM-EDS) and Fourier transform infrared spectroscopy (FT-IR) were used to identify and characterize fibres and mordants in funeral gowns. Of the 23 textile samples tested, 19 were silk while the remaining four were recognized as wool. The presence of iron, aluminium, sodium, and calcium suggests that they were used as mordants. Traces of copper, silica, and magnesium might originate from the contaminants. The large amount of silver indicated the presence of metal wire in one of the dyed silk textiles. SEM images showed that textile fibres were highly degraded.

## 1. Introduction

The study of archaeological textiles is now a rapidly developing field of research, providing important knowledge about ancient dyeing techniques. Historical samples are characterized by the use of various analytical methods and procedures to obtain data on their organic and inorganic components. Improved methods of extracting dyes from textiles have allowed the identification of specific dye sources in some cases [1,2,3]. The identification of dyes in historical objects requires sensitive and selective analytical methods because the amount of available materials is usually limited. Among them, high-performance liquid chromatography with spectrophotometric and mass-spectrometric detectors (HPLC-UV-Vis-MS) is one of the useful tools for the analysis of textile samples containing dyes [4,5,6,7,8,9]. Tandem electrospray ionization mass spectrometry (MS/MS) can identify these compounds and enables conclusions about their structures [10,11,12,13,14]. Energy dispersive X-ray spectroscopy combined with scanning electron microscopy (SEM-EDS), particle induced X-ray emission (PIXE), laser ablation ICP-MS, and laser induced breakdown spectroscopy (LIBS) give information on the mordanting ions and reveals details of ancient dyeing techniques [15,16,17,18,19].

In the past, only natural organic dyes of animal and plant origin have been used to dye textiles. The dyes belong to diverse molecular categories, such as anthraquinones, flavonoids, indigoids, curcuminoids, etc. By identifying dyes in textiles, dyeing techniques and even historical trade routes can be determined [20]. Burial textiles demonstrate our traditions, mores, funerary practices. They were usually a part of the wares chosen to accompany the dead in the hereafter [8]. Textiles recovered from archaeological sites are generally discoloured, fragile, mineralised, and are highly biodeteriorated [21,22,23]. The extent of preservation largely depends on the burial location. A crypt can be regarded as a particular environment where microbial contributes to the decomposition of the organic matrix over a long time [24]. Wool, cotton, and silk are destroyed by various types of microorganisms, and these microorganisms work together to cause them to decompose [25,26,27]. The taphonomic processes affecting exposed human remains also influence the burial clothes condition. Very little is known about the effect of microorganisms on the preservation of natural dyestuffs [16,28,29]. The present study supplements knowledge on this issue.

In this study, twenty-three textile fragments of funeral clothes found in 17th and 18th century crypts were examined. The mild hydrofluoric acid extraction was optimized for the isolation of dyes from burial robes. The dyes were identified by high performance liquid chromatography-mass spectrometry (HPLC-ESI-MS) and quadrupole time-of-flight (QTOF) mass spectrometry using atmospheric pressure electrospray ionization in negative mode. Thirty-four dyes have been discovered, identified or tentatively characterized, some of which have not yet been described. Mordant ions were identified by scanning electron microscopy and energy dispersive X-ray detector (SEM-EDS). Fourier transform infrared spectroscopy (FT-IR) was used for fibre characterisation. The morphology of samples was studied with a scanning electron microscope (SEM). Microorganisms present on textiles, which may be important in the degradation processes of fabrics and dyestuffs, have been identified using next-generation sequencing at the level of genera and species.

## 2. Materials and Methods

### 2.1. Chemicals

Methanol (MeOH) and acetonitrile (ACN) of HPLC grade were purchased from Merck (Darmstadt, Germany). Hydrofluoric acid (HF, 48% in water) was purchased from Sigma-Aldrich (Steinheim, Germany). Dimethyl sulfoxide (DMSO, ACS grade) was obtained from Merck KGaA (Darmstadt, Germany). All aqueous solutions were prepared using Milli Q water.

### 2.2. Equipment

Next generation sequencing was performed on Illumina platform. A scanning electron microscope (SEM) with a secondary electron detector operated in high vacuum mode at an accelerating voltage of 10–20 kV (FEI Quanta FEG 250, Thermo Fisher Scientific, Waltham, MA, USA) was used to examine the morphology of the samples. The elements were identified by energy dispersive spectroscopy (EDS) with ApolloX SDD spectrometer (Ametek, Berwin, PA, USA) at an accelerating voltage of 20 kV. A Nicolet iS50 FTIR spectrometer controlled by the OMNIC v9.2 software package (Thermo Fisher Scientific, Waltham, MA, USA) equipped with Specac Quest Total Reflection Diamond (ATR) accessory and a single reflection diamond was used to record infrared transmission spectra. Chromatographic analysis was performed using Agilent liquid chromatograph series 1290 (Agilent Technology, Waldbronn, Germany) consisting of binary pump G4220A, autosampler G4226A, thermostated column compartment G1316C, diode-array detector G1315C, and triple quadrupole mass spectrometer G6460 with AJS electrospray ionization source. The chromatographic system was controlled with Agilent MassHunter software B 06.01.

### 2.3. Next-Generation Sequencing

#### 2.3.1. DNA Extraction

Genomic DNA was extracted directly from 1 cm^2^ of textile using the Genomic Mini AX Plant (A&A Biotechnology, Gdynia, Poland) with minor modifications. Textile was fragmented with 0.3 g of zirconia beads in LS lysis suspension (A&A Biotechnology, Gdynia, Poland).

#### 2.3.2. NGS Amplicon Library Preparation, and Bioinformatic Analysis

Primers V3-V4 (Illumina, Inc., San Diego, CA, USA), which amplify a region of the 16S rRNA containing the overhang adapter sequences on the 5′ were used to detect bacterial DNA. Next-generation amplicon sequencing and libraries were made and next-generation sequencing was performed using MiSeq 600 cycle V3 (Illumina, Inc., San Diego, CA, USA). The quality control of the data and the method for selecting the operational taxonomic units are presented in Supplementary Materials (Table S1).

### 2.4. Extraction of Dyes

Dyes were extracted from the textile samples (estimated weight 2 mg) in an ultrasonic bath for 0.5 h (2 × 15 min) at a temperature not exceeding 40 °C using 500 µL of 8 M hydrofluoric acid/MeOH/ACN/DMSO (2:1:1:1, *v/v*). The mixture was centrifuged at 9000 rpm for 5 min to separate the particulate matter. The supernatant was stored in a refrigerator for 24 h in order to precipitate proteins. Next, the solution was filtered over a 0.45-μm RC syringe filter.

### 2.5. LC-MS Analysis

The samples (2 μL) were injected onto a Poroshell EC-C18 2.7 µm (3.0 mm × 150 mm) column thermostated at 40 °C. The mobile phase flow rate was 0.4 mL min^−1^, and elution was performed using 0.1% (*v/v*) formic acid in water (solvent A) and ACN/MeOH (1:1; *v/v*) (solvent B) in gradient mode: 10% B to 100% B in 20 min. The UV signal was registered at 254 and 350 nm. All mass-spectrometric scan data (m/z 50–1000) were recorded in negative ionization scan mode. The nebulizer pressure, nitrogen flow rate, drying gas temperature, drying gas flow rate, and sheath gas temperature were 45 psi, 5 L min^−1^, 300 °C, 11 L min^−1^, and 250 °C, respectively. The capillary voltage was 3.5 kV and the fragmentation voltages were 100, 200 and 300 V. The structures of identified dyes were confirmed with Agilent 1290 LC system coupled to Agilent quadrupole time-of-flight (QTOF) mass spectrometer G6540 (Agilent Technologies, Inc. Santa Clara, CA, USA) operated in negative ionization mode in the same chromatographic conditions.

## 3. Results and Discussion

**Figure 1:**
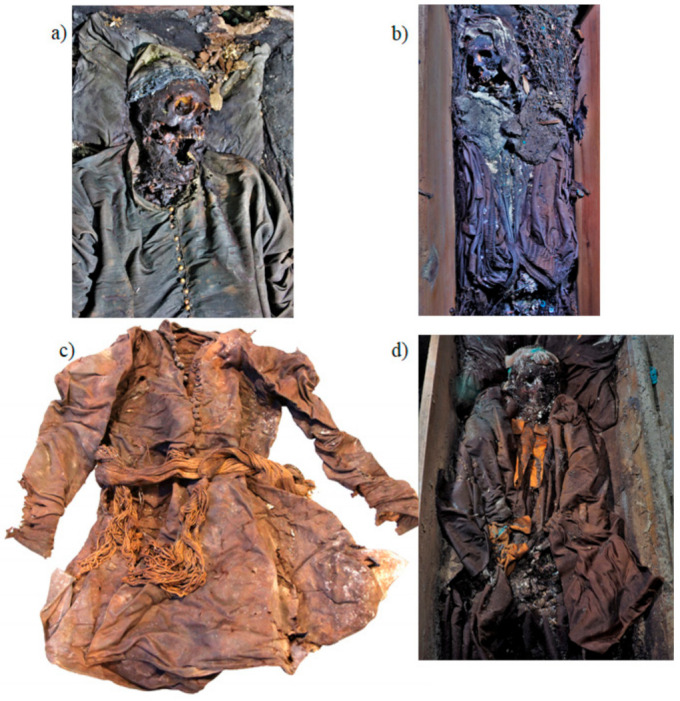
Images of the burial robes found in the crypts: (**a**) zhupan, male national costume C5/b14, (**b**) woman’s dress C5/b11, (**c**) zhupan C8/b6, and (**d**) monk habit C9/b7; photo by M. Łyczak.

In the basement of the Basilica of St. Francis of Assisi in Cracow and in the cloister of the monastery, a total of 22 crypts were found (Figure S1). The contents of nine crypts were opened and inventoried in the church, while the remaining ones were examined by non-invasive methods. In the crypts, 96 burials of members of a religious community, noble and bourgeois families, children and adults dating back to the 17th and 18th centuries were found. During the excavations, a rich collection of clothes was found, among others: men’s national costume, dresses of adult women and young children, chasuble, headgear, stockings, gloves, and footwear (Figure 1). Some of them were worn during life, and some were sewn specially for the grave. The remains rested in richly decorated coffins, painted or upholstered in silk and woollen fabrics. The deceased had herb-filled pillows under their heads. Most were made of linen, but only silk pillows were preserved in the crypts. Among a total of 136 textile samples collected from different parts of burial fabrics and analysed by LC-MS, twenty-three samples were selected for deeper valuation and presented in this paper (see Figure S2 for the object description).

### 3.1. Surface Morphology and Elemental Composition

#### 3.1.1. SEM

**Figure 2:**
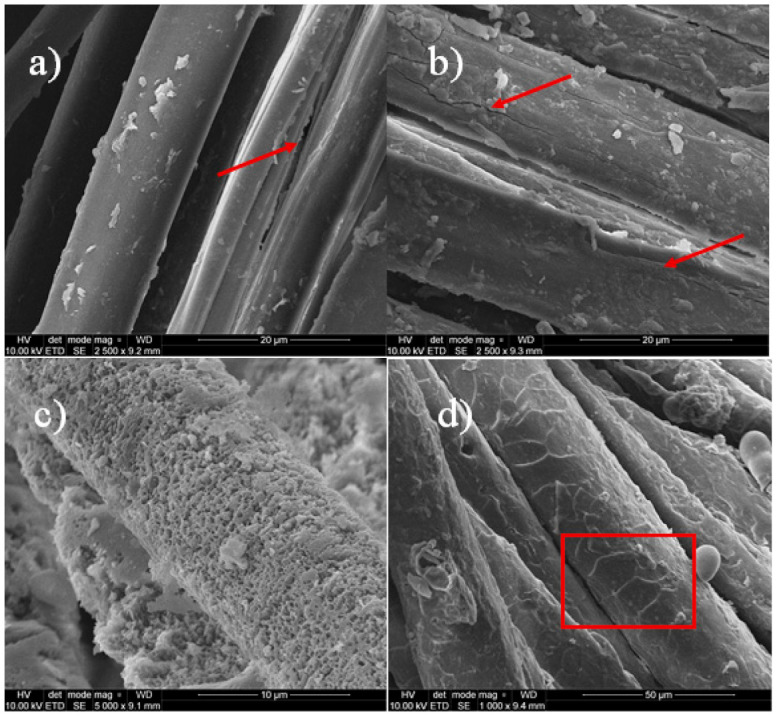
SEM micrographs of threads: (**a**) silk C5/b11, (**b**) silk C5/b9-2, (**c**) silk C5/b5-1, and (**d**) wool C6/b2 (magnitude 2500×). Arrows indicate visible fractures of the silk fibres, rectangle highlights scales characteristic for woollen fibres.

SEM analysis showed that most of the threads were severely degraded and corroded (Figure 2 and Figure S3). The traverse cracking and extensive fibrillation of the fibres were also noticeable. Most samples were contaminated with inorganic particles. Some of them were even severely heavily soiled. Micrographs of the historical fibres show also fracture, tearing, roughened surface, and biological colonization. Two textile samples had a brush-like fracture which is characteristic of fatigue damage (Figure S3a,u) [30]. The degradation of the fibres could have taken place throughout the wearing of the textiles but mainly during the time they were deposited in the crypts and due to taphonomic processes [30,31].

SEM images enable us to distinguish the silk and wool fibres. Silk fibres are relatively smooth with longitudinal striations, while the surface of the wool fibres is covered in scales. A central cavity known as the medulla oblongata can be seen in the larger wool threads but not in silk threads [32]. The fabrics found in the graves were made mainly silk. Among twenty-three investigated textile samples only four were recognized as wool threads. The silk fabrics with a fibre diameter of 7–15 μm were the most representative part of the textile finds. Raw silk is produced by silkworms and is composed of protein fibroin, which is connected by a rubber-like protein called sericin [33]. Fibroin is the structural centre of the silk and sericin is the sticky material surrounding it and protecting fibres against light damage. Studies on the biodegradation of silk in the soil show that bacteria grow vigorously, the fibre is degraded seriously, and the strength is reduced [25]. The fibres of woollen fabrics are more diverse and were from 15 to 30 μm in diameter. Wool is made of keratin, a protein with disulphide bonds that can cross-link the chains in a polymer to form a three-dimensional structure. During the wool ageing, the sulphur bridges are subjected to hydrolysis and oxidation that weakens thread stability [34].

#### 3.1.2. EDS

Most natural colourants are mordant dyes that require the use of a coordination metal to fix the dyestuff molecules to a textile fibre [35] and modulate the final hue of the fibre. Madder, for example, tints violet on iron, red on aluminium, and pink on a tin mordant. EDS analyses were performed to determine the presence of inorganic elements used as a mordant and coming from the crypts as contamination (Table 1).

**Figure 3:**
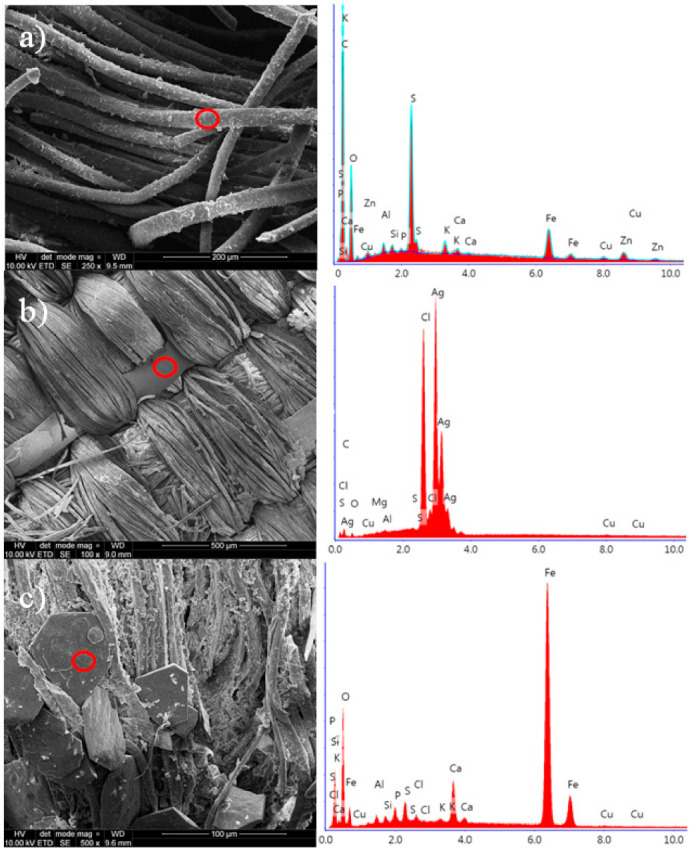
SEM micrographs (on left) and EDS spectra (on right) of: (**a**) woollen threads C8/b7 with high amount of sulphur, (**b**) silk threads C5/b14 with silver strips, and (**c**) silk threads C9/b1-2 containing iron contaminants (magnitude 2500×).

A typical EDS spectrum of the wool fibre specimen is given in Figure 3a. The investigated samples (with two exceptions) did not differ significantly in elemental composition although relative amounts of elements were variant. Several elements were detected, such as carbon and oxygen, which came from wool and silk proteins. In many samples Ca, Fe, Al, K, and traces of Cu were found. Aluminium, Fe, and K probably originate from a mordant essential to obtain fast colours. Copper was found in 19 samples but it is not clear whether it was added deliberately as a mordant agent or whether it was a contamination from the burial.

It is known that iron and copper salts cause darkening of red and yellow mordant dyes, affecting the final colour of the fabrics, whereas aluminium salts do not change the hue of dyed fabrics. No chromium was detected in all of the investigated samples although its salts have been frequently used as mordants in the past [35]. The presence of sulphur is not surprising since this element is found in animal fibres. In most of the samples trace amounts of Na, Mg, Si, and P were also detected. These elements might originate from the utilization of textiles during a lifetime or from residues of richly decorated coffins painted or upholstered in fabrics.

The textile sampled from the Polish national men’s robe *Zhupan* (C5/b14) was woven of two materials that include silver thread (150 μm diameter) and dyed silk yarns (Figure 3b and Figure S3h). Although the metal threads in this fabric are generally in good shape, blackening and tarnishing of the silver tape can be observed. The presence of chlorine in the silver wire indicates corrosion products. The thread sampled from the chasuble (C9/b1-2) has a relatively high content of iron and the SEM micrograph showed that this textile is contaminated by crystalline impurities containing iron, probably iron oxides, (Figure 3c) which may come from the metal fittings or nails of a destroyed lid of a wooden coffin.

#### 3.1.3. FT-IR

FT-IR spectroscopy revealed the presence of the amide I, amide II, and amide III bands, which are characteristic of the proteinaceous fibres. These bands were observed in all of the investigated samples (Figure S4).

The bands at 3277 cm^−1^ in silk fibres were assigned to amide bonds ν(N–H) bending free and H-bonded, the band at 1619 cm^−1^ was assigned to amide I ν(C=O) carbonyl stretching. The band at 1514 cm^−1^ was assigned to amide II δ(N–H) bending and the band at 1229 cm^−1^ due to amide III ν(C–N) stretching were assigned to the peptide bonds (–CONH–) that link the amino acids of proteins together (Figure S4a–f) [23,36]. The 997–978 cm^−1^ bands typical to Gly-Ala sequences in silk fibroin [37], most probably *B. mori* silk, were also detected. The amide I band together with a weaker band around 1674–1695 cm^−1^ were attributed to β-sheet structures of protein. The absorptions at 2930 and 2880 cm^−1^ were due to the C–H asymmetric stretching of aliphatic carbon compounds. The weak absorption band 1400–1390 cm^−1^ observed in the IR spectra of all silk samples, which may be assigned to sericin, indicates that these textile specimens were made of partially degummed or not degummed raw silk. Sericin is usually removed by degumming but in historic silk textiles, sericin may still partially remain [38]. The vibrational bands at 999 and 977 cm^−1^ and the two amide III bands at 1260 cm^−1^ and 1250 cm^−1^ often considered as markers for the crystalline and amorphous structure of silk fibroin were observed in the archaeological silk fibres [39]. The value of the crystallinity index of the investigated silk samples, especially C5/b11, C2/b4-1 and C9/b1-2 fibres, were larger than for *B. mori* silk which indicates fibroin degradation in the amorphous regions.

The amide I and II bands, mainly in-plane N–H bending and the C–N stretching vibration, are characteristic of α-helical conformation and the bands around 1626 and 1516 cm^−1^ were observed in the investigated wool threads. The β-sheet of protein usually gives amide I and II bands between 1635 and 1615 cm^−1^ and between 1535 and 1520 cm^−1^ [40]. The β-sheet conformation cannot be confirmed in the investigated wool threads because the FT-IR spectra did not show any additional shoulders in the amide I and amide II regions. A broad stretching band of amino –NH and phenolic –OH groups at 3272 cm^−1^ were also observed. The weak absorption band about 1230 cm^−1^ was attributed to C–N stretching vibrations and a band near 1040 cm^−1^ was assigned to the presence of ether linkages. Observation of bands in the 590–525 cm^−1^ region was designated to ν(C–S) stretching vibrations in thread samples of woollen fibres, however, these bands are very weak in comparison to the not aged wool sample (Figure S4).

The degradation of the examined fibres was noticeable at the molecular level probably due to biodegradation by microorganisms and a long time ageing in the burials.

### 3.2. Next-Generation Sequencing

In order to determine microbiological biodiversity, there are two different approaches. The classical one concerns the cultivation of microorganisms present in the specimen to be examined and their identification, and the modern one concerns DNA identification by next-generation sequencing. The limitation of the first approach is the possibility of identification of only culturable microorganisms, which in fact allows for the detection of a few percent of species. The limitation of the second one is a relatively low chance to identify microorganisms to the species level, as most frequently it allows for genera or higher levels identification. In the case of identification of microorganisms on archaeological textile, those two approaches were applied so far [21,24,25].

In our study, molecular identification of bacteria by next-generation sequencing has been performed. This approach allowed the identification of five following species: *Clostridium intestinale*, *Escherichia coli*, *Piscicoccus intestinalis*, *Propionibacterium acnes*, *Serratia marcescens,* and bacteria belonging to 17 genera: *Amycolatopsis*, *Anaerococcus*, *Bacillus*, *Cellulosimicrobium*, *Corynebacterium*, *Dokdonella*, *Leuconostoc*, *Mycobacterium*, *Ochrobactrum*, *Pseudonocardia*, *Prevotella*, *Staphylococcus*, *Stenotrophomonas*, *Streptococcus*, *Terracoccus*, *Terriglobus*, *Tsukamurrela*. The microorganisms identified to species or genus level came from different niches (Table 2). Some of them are reported to produce extracellular enzymes and could impact the examined textiles.

**Figure 4:**
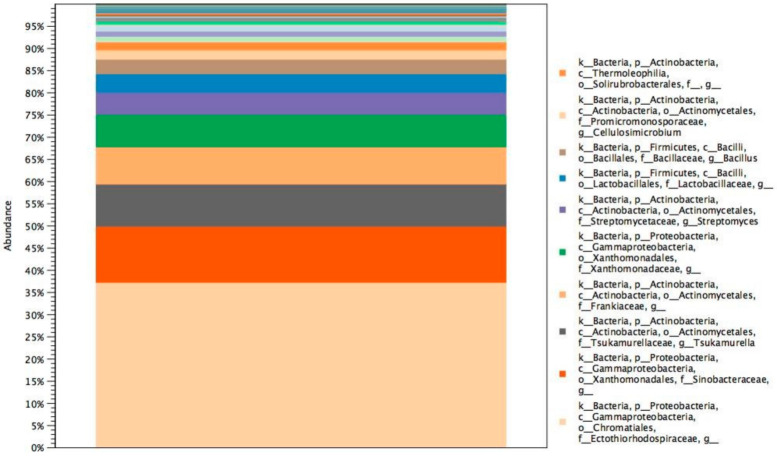
Relative abundance of microorganisms from the phylum to the genus level.

Phyla level is dominated by Proteobacteria which is in accordance with data presented by Szulc et al. [21], even though they examined silk textiles, and in our study woollen textiles were investigated (Figure 4). Identification of all operational taxonomic units is presented in the Supplementary Materials (Table S2). On the studied textiles, not only environmental but also human pathogens and microorganism DNA from microbiota were found. Within the identified environmental genera some bacteria are known for their abilities to degrade compounds that are durable, such as cellulose (*Cellulosimicrobium* sp.) and polymers (*Amycolatopsis* sp.) [41]. Moreover, *Dokdonella sp* is reported as a microorganism able to degrade ethers [42]. Enzymes produced by these bacteria could be involved in the degradation of dyes and may be responsible for the weak retention of colours.

### 3.3. Identification of the Colourants (HPLC-ESI(-)-MS)

Funerary textiles which were in direct contact with the bodies are fragile organic materials that have a tendency to decompose and to degrade. The condition of the fabric samples varied, some of them were stained and fragile, others were slightly stained, strong and in good general condition. Due to exposure to the destructive effects of microorganisms identification of dyes was possible in a limited number of the samples. Most of the samples were brownish but there were also fabrics with blue-green, reddish, and black colour.

**Figure 5:**
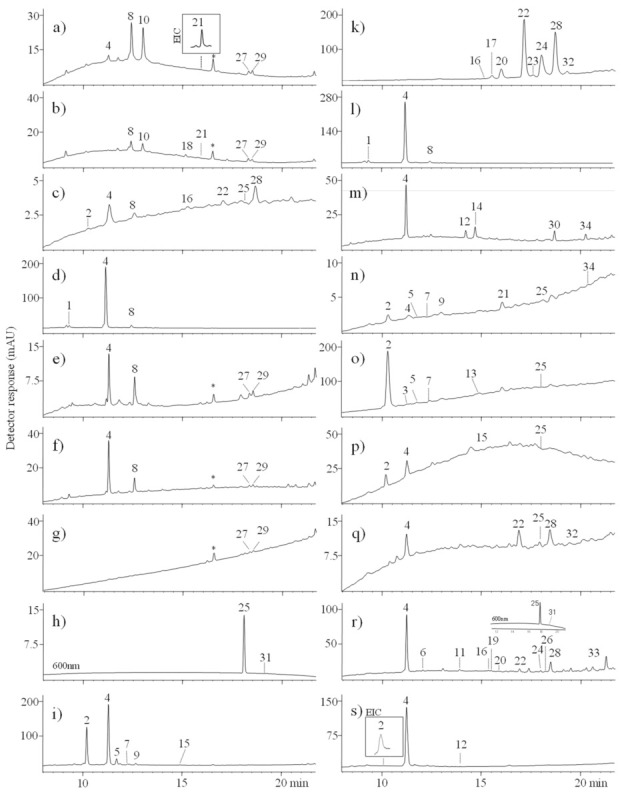
Spectrochromatograms of extracts taken from samples: (**a**) C2/b4-1, (**b**) C2/b4-2, (**c**) C3/b7, (**d**) C5/b9-1, (**e**) C5/b9-2, (**f**) C5/b11, (**g**) C5/b14, (**h**) C5/b25, (**i**) C5/b5-1, (**k**) C5/b5-2, (**l**) C6/b1, (**m**) C6/b2, (**n**) C7/b2, (**o**) C7/b4-1, (**p**) C7/b4-2, (**q**) C8/b6, (**r**) C8/b7, and (**s**) C9/b1-3. For chromatographic conditions, see experimental section, * not ionised in ESI (+/−) mode.

Twenty-three textile samples described in Figure S2 and Table 3 were collected from different parts of burial fabrics found in crypts 2, 3, 5, 6, 7, 8, and 9. MS analysis includes chromatographic separation and full scan MS detection followed by data processing through ion extraction based on the m/z values of the molecular ions of dyes in our database. The results were correlated to spectral chromatographic data in the ultraviolet-visible range. Standardless and unknown dyes were confirmed by high-resolution mass spectroscopy. Figure 5 shows the chromatograms obtained for the extracts of the selected textiles. The retention times, maximum absorbance wavelengths (λ_max_), molecular ions, main fragment ions, and proposed formulae for the compounds are summarised in Table 4. Descriptions of well-known fragmentation of dyestuffs are placed in the Supplementary Materials.

LC-MS analysis showed the presence of indigo dyes in thirteen textile samples (Table 3). The extract of the specimen C5/b25 was blue in appearance and was found to contain only indigotin (m/z 261, λ_max_ 620 nm) and indirubin (m/z 261, λ_max_ 550 nm), the isomeric forms of indigo. The twelve other samples contain also red, yellow and brown dyestuffs besides indigoid colourants. In the dark brown woollen threads (C6/b5) indigotin and ellagic acid were found. The colour of this sample was probably blue-black because of the brown shades of tannins used with iron mordant (found in this specimen by EDS), mingling with blue indigotin tone to give a black colour [43,44]. In the past tannins were not only used as natural dyes or colouring aids, with shades ranging from light yellow to dark brown but also used for silk weighting [45]. In six textile samples, not only indigo and ellagic acid but also red dyestuffs were found, it is supposed its original colour was purple. Alizarin (m/z 239) and purpurin (m/z 255), the main anthraquinone compounds of madder, were found in presently russet-brown threads coded as C8/b6, C9/b1-1, and C9/b1-2. Madder is known for its antimicrobial activity, it can reduce the growth of bacteria on dyed threads, thereby helping the preservation of the textiles [46,47,48].

The burial textile C3/b7 contained indigotin, ellagic acid, red anthraquinones: purpurin, alizarin, and hystazarin (m/z 239), and traces of carminic acid. Carminic acid (m/z 491), is the main dye component in Mexican cochineal (*Dactylopius coccus Costa*), Armenian cochineal (*Porphyrophora hameli Brandt*), and Polish cochineal (*Porphyrophora polonica* L.). The chromatogram of this extract showed also a peak at the retention time of 12.5 min and λ_max_ at 365 nm. The deprotonated molecular ion at m/z 346.2304 and fragment ions at m/z 302.2401, 285.2378, 257.2430, 229.2488, 201.2530, and 173.2581 were found in the mass spectrum of this compound. The fragment ion at m/z 302 [M–H–42]^−^ corresponds to the loss of the CO_2_. The ion at m/z 285 was formed by loss of 17 Da that could correspond to homolytic cleavage of the hydroxyl group. The ions m/z 257, 229, 201, and 173 are formed by subsequent losses of CO molecules (28 Da) which points towards the presence of four CO groups. The tandem mass spectrum suggests that this molecule may be related to the anthraquinones as they share the same fragment ions [M–CO_2_]^−^ and [M–CO_2_–CO]^−^ but we could not provide further explanation. This compound, coded as unidentified 346, was detected in seven of the investigated textile samples and may be a marker of undefined transformation of dyes under burial conditions. To the best of our knowledge, this specific molecule has not been described in the literature before.

The major component in dark brown specimen C7/b4-1 (Figure 5o) was carminic acid with smaller amounts of flavokermesic acid (m/z 313), kermesic acid 7-C-α-glucofuranoside (dc IV), kermesic acid 7-C-β-glucofuranoside (dc VII), and 5-aminokermesic acid (m/z 490), minor colourants present in American and Polish cochineals [49,50]. Apart from red colourants, indigotin was also detected in this specimen, it is supposed that the original colour of this robe was purple.

**Figure 6:**
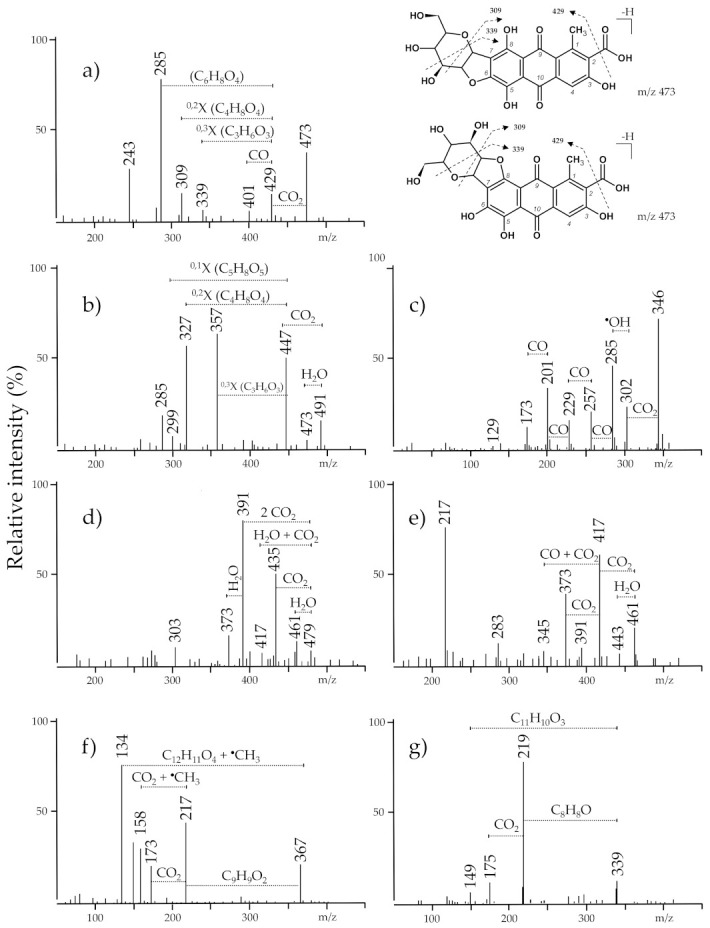
MS spectra acquired in negative ion mode and the proposed fragmentations of: (**a**) dehydrated carminic, (**b**) carminic acid, (**c**) unidentified 346, (**d**) xantholaccaic acid B, (**e**) dehydrated xantholaccaic acid B, (**f**) curcumin, and (**g**) dihydrodemethoxycurcumin.

Textile samples C7/b4-2 and C7/b2 contained carminic acid, *iso*-carminic acids, flavokermesic acid, erythrolaccin, and traces of kermesic acid together with indigotin and ellagic acid. Erythrolaccin ([M–H]^−^ at m/z 285) is a decarboxylated form of kermesic acid with similar fragmentation pathways. Two more colourants were detected in the textile C7/b2. A compound with molecular anion m/z 473 differs by 18 atomic mass units from carminic acid ion (Figure 6a). This compound may be formed from carminic acid by elimination of water involving the hydroxyl substituent at position C-2′ of the sugar moiety and the hydroxyl groups at the C-6 or C-8 position of the aglycone which is promoted by the hydrogen bond between the ether oxygen atom of the sugar ring and the 6- or 8-hydroxyl group [50,51]. This compound formed a fragment ion [M–H–CO_2_]^−^ at m/z 429. The formation of a dihydrofuran moiety leads to changes in the further fragmentation of the sugar ring leading to products that are classical of C-glucosides. It is confirmed by the presence of the ions at m/z 309 of the [^0.2^X–H–CO_2_]^−^ (loss of 120 Da) and at m/z 339 ([^0.3^X–H–CO_2_]^−^, loss of 90 Da). This hypothesis was supported by the ESI(−)-QTOF product ion mass spectrum with the pseudo-molecular peak of [M–H]^−^ at m/z 473.0727, corresponding to the elemental composition of C_22_H_17_O_12_ (mass diff. 0.3 ppm). This colouring compound has not been so far found in the dyer raw material as well as in dyed textiles, and it may be a marker of carminic’s acid transformation due to the taphonomic process. The textile extract C7/b2 (Figure 5 n) contained also a peak eluted at 16.0 min, marked as 21. A precursor ion [M–H]^−^ at m/z 461 and fragment ions at m/z 443 [M–H–18]^−^, m/z 417 [M–H–44]^−^, m/z 373 [M–H–88]^−^, as well as an ion at m/z 217, were found in the mass spectrum of this compound. The observed fragment ions [M–H–H_2_O]^−^, [M–H–CO_2_]^−^, and [M–H–2CO_2_]^−^ are characteristic of laccaic acids but the presence of a fragment ion corresponding to [M–H–2CO_2_–CO] is unexpected for these compounds [7]. This compound could be a derivative of xantholaccaic acid B formed by the loss of water ([M–H]^−^ at m/z 461.0523, elemental composition C_24_H_13_O_10_, mass diff. 1.9 ppm) but the acquired MS/MS spectra were insufficient for determining the exact structure of this compound.

Although red dyes were found in the majority of the examined samples, including some that also contained other dyestuffs, only one textile sample (C5/b5-2) had visible red colour (Figure S2). The chromatogram of this red extract (Figure 5k) revealed the presence of three major peaks (alizarin, xanthopurpurin, purpurin) and five minor peaks namely: hystazarin, munjistin, anthragallol, lucidin, and rubiadin, respectively. All of them are the main anthraquinone constituents in madder.

Carminic acid with a combination of the minor compounds of a scale insets dyes: *iso*-carminic acids, kermesic acid, and earlier mentioned dehydrated carminic acid were detected in the silk textile sample C5/b5-1. This textile was probably of brown colour due to the presence of an abundant amount of ellagic acid (Figure 5i).

Carminic acid with a high amount of ellagic acid of a tannin-producing plant source was found in a brownish sample C9/b1-3. Traces of fisetin, the main dye component in young fustic, was also detected in this extract.

Fisetin and sulfuretin with a molecular anion at m/z 269 were also identified in wool textile C6/b2. Three additional peaks were found in the UV chromatogram of this brown extract (Figure 5m). The main peak at 11.3 min was recognized as ellagic acid while minor peaks, identified as deoxyerythrolaccin and erythrolaccin, are decarboxylated derivatives of flavokermesic and kermesic acids (indicated as compounds 30 and 34).

The peak present at 9.3 min in the chromatograms of two textile extracts C5/b9-1 and C6/b1 was identified as protosappanin B (m/z 303 [M–H]^−^). Protosappanin B together with brazilin, brazilein, and other homoisoflavones are the main soluble redwood dyes of various species of the genus *Caesalpinia*. It is known that brazilein is not stable for wash fastness and light and it undergoes photodegradation to produce urolithin C (m/z 243 [M–H]^−^), also referenced as compound-type C [52], commonly used as a marker for the identification of soluble redwood in aged artefacts [12,53,54]. Specimens C5/b9-1 and C6/b1 did not contain this marker which probably is not formed in a specific crypt environment.

Ellagic acid, which is associated with the presence of tannins, was detected in most of the samples and was particularly common in samples C5/b9-1, C5/b9-2, C5/b11, C5/b5-1, C6/b1, C6/b2, C8/b7, C9/b1, and C9/b7. In the past, tannin dyes were often applied on proteinaceous fibres, especially wool and silk [35,55]. The tannin-related compound detected in the fabrics could indicate the use of these compounds to promote the interaction between the coloured chemical species and the fibres and possibly also to modify the final shade of the textiles [43,45].

Traces of curcumin were found in six burial textiles: C2/b4-1, C2/b4-2, C5/b9-2, C5/b11, C5/b14 and C5/b19. Its presence was confirmed by the molecular anion peak ([M–H]^−^ at m/z 367) and daughter ions (Figure 6f) [56,57]. Curcumin together with demethoxycurcumin with the molecular anion of m/z 337 [M–H]^−^) and bis-demethoxycurcumin (m/z 307 [M–H]^−^) are the main colouring matter present in various species of Curcuma, *Curcuma longa* L. being the most common source. However, demethoxycurcumin and bis-demethoxycurcumin were not found in all of the above mentioned textile extracts. We detected a compound with a mass peak at m/z 339 (Figure 6g) and fragment ions: m/z 219 [M–H–C_8_H_8_O]^−^, 175 [M–H–C_8_H_8_O–CO_2_]^−^, and 149 [M–H–C_11_H_10_O_3_]^−^. This molecule differs from demethoxycurcumin by saturation at carbons 6 and 7 and it could be dihydrodemethoxycurcumin. The hypothesis was confirmed by an ESI(-)-QTOF product ion mass spectrum, in which the peak [M–H]^−^ was observed at m/z 339.1228 (corresponding to the elemental composition of C_20_H_19_O_5_, mass diff. −2.9 ppm). Traces of dihydrodemethoxycurcumin were found in fresh turmeric rhizome [57] but never before in dyed textiles. Dihydrodemethoxycurcumin is commonly known as a metabolite of curcumin. Reduction of curcumin occurs mainly at heptadienone chain double bonds to form di-, tetra-, and octahydrocurcumin. These transformations are facilitated by numerous enzymes, e.g., NADPH-dependent reductase, alcohol dehydrogenase, and microsomal enzymes [58]. Several metabolites of curcumin have been already known including dihydrocurcumin, curcumin glucuronide, curcumin sulfate, etc. Some of them have been found to be very stable, even more stable than curcumin [59]. Turmeric dyes were widely used in the past despite the poor fastness to light and washing. They were frequently used in association with other dyestuffs, e.g., with indigo to obtain a green hue [60,61] and were also popular in the 17th century for shading red silk dyed with cochineal [35,62].

In three specimens (C5/b9-2, C5/b14 and C5/b19) indigotin was found apart from curcumin and dihydrodemethoxycurcumin suggesting that the original colour of these robes was green. In a fragment of a woman’s dress (textile sample C5/b11), curcumin dyes were mixed with ellagic acid probably to obtain a brown shade of a dress. Two burial textiles (C2/b4-1 and C2/b4-2) were dyed with a cocktail containing curcumins, ellagic acid, and lac dye colourants. Close inspection of mass spectra revealed also the presence of xantholaccaic acid B (m/z at 479) and its dehydrated derivative discussed earlier (compound 21, m/z at 461). C2/b4-2 fibre extract contained compound 18 with a molecular anion [M–H]^−^ at m/z 502 and five fragment ions at m/z 484 [M–H–H_2_O]^−^, 458 [M–H–CO_2_]^−^, 440 [M–H–H_2_O–CO_2_]^−^, 414 [M–H–2CO_2_]^−^, and 386 [M–H–2CO_2_–CO]^−^, respectively. The loss of one or two CO_2_ molecules followed by the loss of water is a fragmentation pathway typical for laccaic acids, but decarbonylation of one or more of the keto groups is the main fragmentation reaction of anthraquinoids [7]. The structure of this component was tentatively identified as a derivative of xantholaccaic acid A deprived by water molecule based on ESI(-)-QTOF product ion mass spectrum with the pseudo-molecular peak of [M–H]^−^ at m/z 502.0788, corresponding to the elemental composition of C_26_H_16_NO_10_ (mass diff. 1.6 ppm), although it is not possible to conclude the exact location of the dehydration reaction.

Only one of the burial fabrics (specimen C8/b7), sampled from a woman’s gown made especially for the grave, still shows deep black colour (Figure S2). Black is one of the most difficult colours to perform, particularly when using natural dyes [63]. Most traditional European black dyeing processes relied on a cocktail of different ingredients, including a combination of blue, red, brown based on a source of tannin, and yellow dyes, often including metallic mordants [64,65]. Red anthraquinones: alizarin, purpurin, xanthopurpurin, hystazarin, and anthragallol; blue indigoids: indigotin and indirubin; yellow flavonoids: chryoseriol, rhamnazin, quercetin and quercitrin, as well as ellagic acid were detected in this black sample (Figure 5r). Quercetin was confirmed by its UV spectrum, the retention time corresponding to the reference standard material, and molecular anion [M–H]^−^ at m/z 301. Compound **6** with the molecular anion [M–H]^−^ at m/z 447 and fragment ion at m/z 301 formed by loss of 146 mass units (elimination of rhamnose unit from quercetin-*O*-rhamnoside) is a unique example of the colourant containing preserved *O*-glycoside bond among the investigated samples. It is difficult to determine the raw material from which the dye originated because all of the above mentioned flavonoids and their glycosides are widely distributed in the plant kingdom, e.g., in Persian berries, weld, and some other plants [35].

## 4. Conclusions

A systematic multi-technique investigation of the dyeing burial textiles found in 17th and 18th century crypts in Poland was used in this work. The condition of the fabric samples varied. Some of them were heavily damaged. The others were slightly stained, strong, and in good general condition. Most of the fabrics were brownish but there were also fabrics with blue-green, reddish, and black colour. Application of next-generation sequencing enabled DNA identification of microorganisms at genus and species level. DNA of isolates belonging to genus *Dokdonella* with high biodegradation ability was found in the investigated textiles. SEM-EDS and FT-IR were used for the fibre identification and fibre degradation statement. SEM micrographs of the wool fibres displayed scale structures and cylindrical shapes with nodular thickening along their length, while the surface of the silk fibre is rather smooth with longitudinal striations. EDS analyses reveal Ca, Fe, Al, K, and traces of Cu, as well as carbon and oxygen derived from wool and silk proteins. Aluminium, Fe and K are most likely derived from a mordant required to obtain fast colours. Traces of copper, silica, and magnesium may have come from the use of textiles over a lifetime or from contaminants in the crypts. Silver thread woven together with dyed silk yarns has been found in textile sampled from the Polish national men’s robe zhupan.

Various dyestuff classes were isolated from textile samples that had direct contact with human remains using a gentle extraction method based on hydrofluoric acid. A number of colour organic compounds present in silk and wool fibre extracts were identified using high resolution mass spectrometry, while characteristic fragmentation pathways provided additional information on the structures of the analytes. Dyes found in the silk and woollen textiles indicates that dyes of both plant and insect origin were used for dyeing. The dyes belong to several classes of natural organic colourants: indigoids, anthraquinones, tannins, flavonoids, and curcuminoids. Ellagic acid, indigotin, carminic acid, and madder dyer components dominated in the investigated burial textiles. The curcumin ingredients found in six textiles samples have never been identified in any ancient Polish textiles so far. Five compounds: dihydrodemethoxycurcumin, dehydrated carminic acid, unidentified colourant 346, and dehydrated xantholaccaic acid A and B were for the first time found in historical textiles. *O*-glucoside form of flavonoid dye was detected only in one well preserved black textile while *C*-glucosides of anthraquinones seem to be more stable under burial conditions.

## Figures and Tables

**Table 1:** Composition of elements (in atomic %) based on EDS analysis.

Element (at%)	C	O	Na	Mg	Al	Si	P	S	Cl	K	Ca	Fe	Cu	Ag	Zn	Thread Diameter(μm)	Thread Type
Sample Code																	
C2/b4-1	72.5	27.0	0.5	*	*	*	*	*	*	0.5	1.0	0.5			*	8–9	silk
C2/b4-2	71.0	28.0	0.5	*	*	*	*	*	*	*	0.5	*	*			7–8	silk
C3/b7	56.5	38.0	*	*	0.5	*	1.0	*	*	0.5	1.5	0.5	*		*	7–10	silk
C5/b5-1	72.0	27.0	-		0.5	*	*	*	*	0.5	*	-	*	-	0.5	7–8	silk
C5/b5-2	70.0	27.0	0.5	*	0.5	*	*	0.5	*	0.5	0.5	*	*	-		20–25	wool
C5/b9-1	57.0	39.0	0.5	*	*	*	0.5	*	*	0.5	1.5	0.5	-	-	*	10–12	silk
C5/b9-2	63.0	34.0	-	*	*	*	1.0	*	-	*	0.5	0.5	*	-	-	10–14	silk
C5/b11	72.0	26.0	-		*	-	*	*	-	-	0.5	*	*	-	*	10–11	silk
C5/b14	21.0	15.5	-	1.0	1.0	-		1.0	30.0	-	-	-	0.5	30.0	*	11–12 150	silk silver
C5/b19	72.0	27.0	-	*	*	0.5	*	0.5	-	*	0.5	*	*	-	-	7–8	silk
C5/b25	75.0	25.0	-	*	*	*	*	*	-	-	*	-	*	-	*	10–11	silk
C6/b1	52.5	40.5	-	-	0.5	*	2.0	*	*	0.5	1.5	2.0	*	-	*	10–13	silk
C6/b2	63.5	32.5	*	*	0.5	1.0	*	1.0	*	*	1.0	2.5	-	-	-	15–30	wool
C6/b5	61.5	32.0	-	*	0.5	0.5	*	2.0		*	2.0	1.0	*	-	-	20–21	wool
C7/b2	61.5	35.0	0.5	*	0.5	*	0.5	*	*	*	1.0	*	*	-	*	8–9	silk
C7/b4-1	72.0	26.0	-	*	*	*	*	0.5	*	*	0.5	*	*	-	-	13–14	silk
C7/b4-2	72.0	27.0	-		*	*	-	*	-	-	*	*	*	*	-	9–10	silk
C8/b6	66.0	30.0	-	*	*	*	*	*	*	0.5	1.5	*	*	-	-	9–11	silk
C8/b7	73.0	24.0	-	-	*	*	*	1.5	-	*	*	0.5	*	-	*	20–30	wool
C9/b1-1	75.0	22.5	0.5	*	*	*	*	0.5	*	*	*	*	*	-	-	9–12	silk
C9/b1-2	48.0	40.0	-	-	0.5	*	0.5	0.5	*	*	1.0	9.0	*	-	-	10–14	silk
C9/b1-3	63.0	30.0	0.5	-	*	*	1.0	0.5	*	1.5	1.0	1.5	*	-	*	12–13	silk
C9/b7	69.0	29.5	*	*	*	*	*	*	*	*	0.5	*		-	-	10–15	silk

Uncertainty of carbon and oxygen content is +/− 3% and for other elements +/− 0.1%; * elements detected below 0.1%.

**Table 2:** Genus and species identified by next-generation sequencing.

Niche	Human Pathogens	Human Microbiota	Animal Microbiota	Environment
Species	*Serratia marcescens*	*Escherichia coli*	*Piscicoccus intestinalis*	-
-	-	*Propionibacterium acnes*	*Clostridium intestinale*	-
Genus	*Mycobacterium*	*Anaerococcus*	*Corynebacterium*	*Dokdonella*
-	-	*Prevotella*	-	*Leuconostoc*
-	-	*Streptococcus*	-	*Amycolatopsis*
-	-	*Staphylococcus*	-	*Stenotrophomonas*
-	-	-	-	*Terracoccus*
-	-	-	-	*Tsukamurrela*
-	-	-	-	*Cellulosimicrobium*
-	-	-	-	*Bacillus*
-	-	-	-	*Terriglobus*
-	-	-	-	*Ochrobactrum*
-	-	-	-	*Pseudonocardia*

**Table 3:** Summary of identified colourants, possible dyestuffs sources and type of fibres.

Sample Code	Identified Compounds	Biological Source
C2/b4-1	Curcumin, dihydrodemethoxycurcumin, ellagic acid, xantholaccaic acid B, dehydrated xantholaccaic acid B, unidentified 346	Turmeric, tannin plant, lac dye
C2/b4-2	Curcumin, dihydrodemethoxycurcumin, ellagic acid, xantholaccaic acid B, dehydrated xantholaccaic acid B, dehydrated xantholaccaic acid A, unidentified 346	Turmeric, tannin plant, lac dye
C3/b7	Indigotin, carminic acid, alizarin, purpurin, hystazarin, ellagic acid, unidentified 346	Indigo, cochineal, madder, tannin plant
C5/b5-1	Carminic acid, dc IV, dc VII, dehydrated carminic acid, ellagic acid, kermesic acid	Cochineal, tannin plant
C5/b5-2	Alizarin, anthragallol, purpurin, hystazarin, xanthopurpurin, munjistin, lucidin, rubiadin	Madder
C5/b9-1	Protosappanin B, ellagic acid, unidentified 346	Redwood, tannin plant
C5/b9-2	Indigotin, dihydrodemethoxycurcumin, ellagic acid, curcumin, unidentified 346	Indigo, tannin plant, turmeric
C5/b11	Ellagic acid, dihydrodemethoxycurcumin, curcumin, unidentified 346	Tannin plant, turmeric
C5/b14	Indigotin, curcumin, dihydrodemethoxycurcumin	Indigo, turmeric
C5/b19	Indigotin, curcumin, dihydrodemethoxycurcumin	Indigo, turmeric
C5/b25	Indigotin, indirubin (traces)	Indigo
C6/b1	Protosappanin B, ellagic acid, unidentified 346	Redwood, tannin plant
C6/b2	Ellagic acid, deoxyerythrolaccin, erythrolaccin, fisetin, sulfuretin	Tannin plant, cochineal, young fustic
C6/b5	Indigotin, ellagic acid	Indigo, tannin plant
C7/b2	Carminic acid, dc IV, dc VII, dehydrated carminic acid, erythrolaccin, dehydrated xantholaccaic acid B, ellagic acid, indigotin	Cochineal, tannin plant, indigo, lac dye
C7/b4-1	Carminic acid, dc IV, dc VII, flavokermesic acid, 5-aminokermesic acid, indigotin	Cochineal, indigo
C7/b4-2	Ellagic acid, carminic acid, kermesic acid (traces), indigotin	Tannin plant, indigo, cochineal
C8/b6	Ellagic acid, indigotin, alizarin, purpurin, rubiadin	Tannin plant, indigo, madder
C8/b7	Ellagic acid, indigotin, indirubin, alizarin, purpurin, xanthopurpurin, hystazarin, anthragallol, rhamnazin, chrysoeriol, emodin, quercetin, quercitrin	Tannin plant, indigo, madder, undefined flavone producing plant
C9/b1-1	Ellagic acid, alizarin, indigotin (traces)	Tannin plant, madder, indigo
C9/b1-2	Ellagic acid, alizarin, munjistin, rubiadin, indigotin	Tannin plant, madder, indigo
C9/b1-3	Carminic acid, ellagic acid, fisetin	Tannin plant, cochineal, young fustic
C9/b7	Ellagic acid	Tannin plant

“Tannin plant” is not indicative of a particular dye source. “Indigo” refers to several plants that produce indigotin and indirubin.

**Table 4:** Spectrochromatographic data of the dyes extracted from the investigated textiles.

Peak No.	t_R_ (min)	[M–H]^−^ (m/z)	Fragment Ions (m/z)	Proposed Identification	Colour	λ_max_ (nm)
1	9.3	303	273, 229	protosappanin B	red	287, 390
2	10.1	491	447, 473, 357, 327, 299, 285	carminic acid	red	276, 490
3	11.2	490	446, 356	5-aminokermesic acid	red	-
4	11.3	301	245, 183, 169, 139, 124	ellagic acid	cream	275, 365
5	11.6	491	447, 357, 327, 299	kermesic acid 7-C-glucofuranoside (dc IV)	red	276, 490
6	12.2	447	301, 211, 151	quercitrin	yellow	257, 349
7	12.3	491	447, 357, 327, 299	kermesic acid 7-C-glucofuranoside (dc VII)	red	276, 490
8	12.5	346	302, 285, 257, 229, 201, 173	unidentified	-	260, 365
9	12.6	473	429, 401, 339, 309, 285, 243	dehydrated carminic acid	red	245, 380
10	13.0	479	461, 435, 417, 391, 373	xantholaccaic acid B	red	260, 365
11	14.0	301	232, 151, 121	quercetin	yellow	255, 360
12	14.2	285	241, 229, 149, 135, 121	fisetin	yellow	355, 360
13	14.4	313	285, 269, 241, 197	flavokermesic acid	red	280, 430
14	14.7	269	241, 225, 213, 195, 135, 133	sulfuretin	yellow	256, 396
15	14.9	329	285, 257, 269, 213, 185, 169	kermesic acid	red	270, 490
16	15.2	239	211, 195, 183, 167	hystazarin	red	282, 413
17	15.4	283	239, 211	munjistin	red	287, 492
18	15.4	502	484, 458, 440, 414, 386	dehydrated xantholaccaic acid A	red	-
19	15.5	299	284, 256, 243, 227, 199	chryoseriol	yellow	270, 350
20	15.9	255	239, 227, 183	anthragallol	red	279, 405
21	16.0	461	443, 417, 391, 373, 345	dehydrated xantholaccaic acid B	red	210, 245
22	16.8	239	211, 183, 167, 151	alizarin	red	280, 430
23	17.5	269	251, 239, 223, 211, 195	lucidin	red	280, 420
24	18.0	239	211, 195, 167	xanthopurpurin	red	250, 420
25	18.1	261	233, 217	indigotin	blue	288, 620
26	18.2	329	314, 301, 299, 286, 271, 258	rhamnazin	yellow	256, 371
27	18.3	339	337, 219, 175, 149	dihydrodemethoxycurcumin	yellow	260, 400
28	18.3	255	227, 183	purpurin	red	255, 480
29	18.5	367	217, 173, 158, 149, 134	curcumin	yellow	250, 415
30	18.7	269	241, 225, 213, 197	deoxyerythrolaccin	red	260, 440
31	19.2	261	233, 217	indirubin	blue	290, 550
32	19.8	253	225, 197	rubiadin	red	245, 410
33	20.1	269	241, 225, 197	emodin	orange	250, 290
34	20.2	285	257, 241, 229, 213, 185	erythrolaccin	red	265

## Data Availability

The data presented in this study are available in article and Supplementary Materials file.

## Supplementary Materials

The following are available online at https://www.mdpi.com/article/10.3390/ma14133535/s1, Figure S1: Schematic plan of the Basilica St. Francis of Assini in Cracow and location of the crypts in the church and in the cloister; Figure S2: Images and description of the burial textile samples; Figure S3: Compilation of SEM images of investigated threads at a magnification of 100×, 500× and 2500×; Figure S4: FT-IR spectra of the selected silk and woollen fibres; Table S1: Data QC and OUT clustering; Table S2: Genus and species identified by next-generation sequencing.
