# Supplementary material for: Multi-Technique Investigation of Grave Robes from 17th and 18th Century Crypts Using Combined Spectroscopic, Spectrometric Techniques, and New-Generation Sequencing
Source: Materials (Basel). 2021 Jun 24;14(13):3535. doi: 10.3390/ma14133535 (PMC8269536; doi:10.3390/ma14133535)
Supplement: Supplementary file 1 [file materials-14-03535-s001.zip › materials-1244209-supplementary.pdf]

materials

Supplementary Materials

Multi-Technique Investigation of Grave Robes from 17th and 18th Century Crypts Using Combined Spectroscopic, Spectrometric Techniques, and New-Generation Sequencing

Magdalena Śliwka-Kaszyńska [1,*], Marek Ślebioda [2], Anna Brillowska-Dąbrowska [3], Martyna Mroczyńska [3], Jakub Karczewski [4], Anna Marzec [5], Przemysław Rybiński [6] and Anna Drążkowska [7]

[1] Department of Organic Chemistry, Faculty of Chemistry, Gdańsk University of Technology (Gdańsk Tech), 80-233 Gdańsk, Poland
[2] Perlan Technologies, Sp. z.o.o., 02-785 Warszawa, Poland; m.slebioda@wp.pl
[3] Department of Molecular Biotechnology and Microbiology, Faculty of Chemistry, Gdańsk University of Technology (Gdańsk Tech), 80-233 Gdańsk, Poland; annbrill@pg.edu.pl (A.B.-D.); marmroc1@pg.edu.pl (M.M.)
[4] Faculty of Applied Physics and Mathematics, Gdańsk University of Technology (Gdańsk Tech), 80-233 Gdańsk, Poland; jkarczew@mif.pg.gda.pl
[5] Institute of Polymer and Dye Technology, Faculty of Chemistry, Lodz University of Technology, 90-924 Lodz, Poland; anna.marzec@p.lodz.pl
[6] Institute of Chemistry, Faculty of Natural Science, The Jan Kochanowski University, 25-369 Kielce, Poland; przemyslaw.rybinski@ujk.edu.pl
[7] Faculty of History, Nicolaus Copernicus University in Torun, 87-100 Torun, Poland; annadr@umk.pl
* Correspondence: magkaszy@pg.edu.pl; Fax: +48-58-347-2694

Table S1. Data QC and OTU Clustering.

**Trim Reads**	
Quality trim	true
Quality limit	0.05
Ambiguous trim	true
Ambiguous limit	2
Trim adapter list	
Automatic read-through adapter trimming	true
Remove 5' terminal nucleotides	false
Number of 5' terminal nucleotides	1
Remove 3' terminal nucleotides	false
Number of 3' terminal nucleotides	1
Discard short reads	true
Minimum number of nucleotides in reads	15
Discard long reads	false
Maximum number of nucleotides in reads	1000
**Filter Samples Based on Number of Reads**	
Minimum number of reads	100
Minimum percent from the median	50
**OTU Clustering**	
OTU picking	Reference based OTU clustering
OTU database	Greengenes v13_8 91%
Similarity percent specified by OTU database	true
Allow creation of new OTUs	true
Taxonomy similarity percentage	80
Similarity percentage	97.0

Minimum occurrences	2
Fuzzy match duplicates	false
Find best match	true
Chimera crossover cost	3
Kmer size	6
Mismatch cost	1
Minimum score	40
**OTU Clustering**	
Gap cost	4
Maximum unaligned end mismatches	5
Include all reads	false

Table S2. Operational Taxonomic Units.

Name	Taxonomy	Sequence
1046116	k__Bacteria, p__Bacteroidetes, c__Flavobacteriia, o__Flavobacteriales, f__[Weeksellaceae], g__, s__	CCTACGGGAGGGAGCAGTGAGGAATATTGGAC-<br>CATGGGTGGGAGCCTGAT-<br>TCCGCCATTCCCCGTGGAGGAAGAAAGTCTTATGGATTGTA<br>AACTGCTTTTATATAGGGATAAACCTACTCTCGAGAGGG-<br>TAGCTGAAGGTACTATATGAA-<br>TAAGCACCGGGTAAATCCGTGGCCGCAGCCGCGGTAATACC<br>GAGGGTGCGAAC-<br>GTTATCCCGACTTATTGGGGTTAAAAGGGCCGTAA-<br>GCGGGTTATTAAGTCAGTGGTGAAATCTCATAGCTCAACTA<br>TGAAACTGCCATTGATACTGATAGTCTTGAGTATATTT-<br>GAAGTAGCTGGAATAAGTAGTG-<br>TAGCGGTGAAATGCATAGATATTACTTAGAACACCAATAGC<br>GAAGGCAGGTTACTAAGATATTACTGACGCTGAGGGACGA-<br>GAGCGTAGGGAGCGAACAGGATTAGATACCCTGGTAGTC
865748	k__Bacteria, p__Actinobacteria, c__Actinobacteria, o__Actinomycetales, f__Frankiaceae, g__, s__	CCTACGGGAGGCAGCAGTGGGGAA-<br>TATTGCACAATGGGCGAAAGCCTGATGCAGCGAC-<br>GCCGCGTGAGGGATGACGGCCTTCGGGTTGTAAGCCTCTTT<br>CAGCTCCGACGAAGCGAAAGTGACGGTAGGAGCAGAA-<br>GAAGCACCGGCCAACTACGTGCCAG-<br>CAGCCGCGGTAATACGTAGGGTGCAAGCGTTGTCCGGAATT<br>ATTGGGCGTAAAGAGCTCGTAGGCGGCTT-<br>GTCGCGTCGGCTGTGAAAAC-<br>CTGGGGCTCAACCCCGGGCCTGCAGCCGATACGGGCAAGCT<br>AGAGTTCGGTAGGGGAGACTGGAATTCCTGGTG-<br>TAGCGGTGAAATGCGCAGATATCAGGAG-<br>GAACACCGGTGGCGAAGGCGGGTCTCTGGGCCGATACTGA<br>CGCTGAGGAGCGAAAGCGTGGGGAGCGAACAGGATTAGA-<br>TACCCTGGTAGTCC
853703	k__Bacteria, p__Actinobacteria, c__Acidimicrobiia, o__Acidimicrobiales, f__, g__, s__	CCTACGGGAGGCAGCAGCAGGGAA-<br>TATTGCACAATGGGCGAAAGCCTGATGCAGCAAC-<br>GCCGCGTGGGGGATGAAGGCCTTCGGGTTGTAAACCCCTTT<br>CAGGAGGGAAGAAATTGACGGTACCTCCAAAAGAA-<br>GCCCCGGCCAACTACGTGCCAG-<br>CAGCCGCGGTAATACGTAGGGGGCGAGCGTTGTCCGGAATC<br>ATTGGGCGTAAAGAGCTCGTAGGCGGCTCAGTTAG-<br>TCGGCTGTGAAATCCCAGGGCTCAACCCTGGAACTGCAGTC

		GATACTGCTGTGGCTAGAGTTTGGTAGAGGA-<br>GAATGGAATTCCCGGTGTAGCGGTG-<br>GAATGCGCAGATATCGGGAGGAACACCAGTAGCGAAGGCG<br>GTTCTCTGGGCCAATACTGACGCTGAGGAGCGAAA-<br>GCGTGGGGAGCAAACAGGATTAGATACCCTGGTAGTC
796529	k__Bacteria, p__Acidobacteria, c__Solibacteres, o__Solibacter-ales, f__Solibacteraceae, g__, s__	CCTACGGGAGGCAGCAGTGGGGAATCTT-<br>GCACAATGGAGGCAACTCTGATGCAGCGAC-<br>GCCGCGTGAGCGAAGAAGCCCTTCGGGGTGTAAAGCTCTTT<br>CGGCCGGGACGATTATGACGGTACCGGCAGAAGAA-<br>GCTGCGGCTAACTACGTGCCAG-<br>CAGCCGCGGTAATACGTAGGCAGCAAGCGTTGTTCGGAATT<br>ACTGGGCGTAAAGAGTGTG-<br>TAGGCGGCGTTCTAAGTCCCGTGTGAAATCTCCCGGCTCAA<br>CCGGGAGGGGGCGCGGAAACTGGAGTGCTCGAGCGTGG-<br>GAGAGGAAAGCG-<br>GAATTCCTGGTGTAGCGGTGAAATGCGTAGAGATCAGGAGG<br>AACACCAGCGGTGTAGACGGCTTTCTGGACCATAGCTGAC-<br>GCTGAGACACGAAAGCGTGGG-<br>GAGCAAACAGGATTAGATACCCTGGTAGTC
610440	k__Bacteria, p__Firmicutes, c__Bacilli, o__Bacillales, f__, g__, s__	CCTACGGGAGGCAGCAGTAGGGAATCATCCGCAATGGGG-<br>GAAACCCTGACGGTGCAAC-<br>GCCGCGTGAGCGAGGAAGGTTTTCGGATCGTAAAGCTCTGT<br>TGTGGAGGAAGAACGACCTTCAGAG-<br>GAAATGCTGAGGGAGTGACGGTACTTCACGAGGAA-<br>GCCCCGGCTAACTACGTGCCAGCAGCCGCGGTAATACGTAG<br>GGGGCGAGCGTTGTCCGGAATGATTGGGCGTAAAGGGCAC-<br>GCAGGCGGCTTTTTAAGTCTGATGTGAAAGGCCCCGGCTTA<br>ACCGGGGAGGTGCATTGGAAACTGAAGAGCTTGAGTCCAG-<br>GAGAGGGGAGTGGAATTCCAC-<br>GTGTAGCGGTGAAATGCGTAGAGATGTGGAGGAACACCAG<br>TGGCGAAAGCGGCTCTCTGGCCTGAGACTGAC-<br>GCTGAGGTGCGAAAGCGTGGG-<br>GAGCGAACAGGATTAGATACCCTGGTAGTCC
590637	k__Bacteria, p__Actinobacteria, c__Acidimicrobiia, o__Acidimi-crobiales, f__, g__, s__	CCTACGGGAGGCAGCAGTGGGGAATCTTGCG-<br>CAATGGGCGAAAGCCTGACGCAGCAAC-<br>GCCGCGTGAGGGATGAAGGTCTTCGGGTTGTAAACCTCTTT<br>CAAGCAGGGACGATTGTGACGGTACCTGCAGAAGAA-<br>GCTCCGGCCAACTACGTGCCAG-<br>CAGCCGCGGTGATACGTAGGGAGCGAGCGTTGTCCGGATTC<br>ATTGGGCGTAAAGAGCTCGTAGGCGGCTCAG-<br>TAAGTCAGGTGTGAAATCCTCAGGCTCAAC-<br>CTGAGGTCGCCATCTGATACTGCTGTGGCTAGAGCCCGGTA<br>GGGGTCCACGGAATTCCTGGTGTAGCGGTGAAATGCG-<br>CAGATATCAGGAGGAACAC-<br>CGGTGGCGAAGGCGGTGGACTGGGCCGGTGCTGACGCTGA<br>GGAGCGAAAGCGTGGGGAGCGAACAGGATTAGA-<br>TACCCTGGTAGTC
575079	k__Bacteria, p__Chloroflexi, c__TK17, o__, f__, g__, s__	CCTACGGGAGGCAGCAGCAAGGAATTTTGCACAATCGAC-<br>GCAAGTCGGATGCAGCAAC-<br>GCCGCGTGGGGGAAGAAGGCCTTCGGGTCGTAAACCCCTTT

		TGCGAGGGAAGAAGATCTGACGGTACCTCGCGAA-<br>TAAGTCACGGCTAACTACGTGCCAG-<br>CAGCCGCGGTAATACGTAGGTGGCAAACGTTGTCCGGATTC<br>ACTGGGCGTAAAGCGCGCGCAGGCGGGCTGATCAG-<br>TCTGGGGTGAAATCTCTCGGCTCAAC-<br>CGGGAGAGGCCCTCAGATACTGTCAGCCTTGAGGTGCCTAG<br>AGGAGAGTGGAATTCAGGGTGTAGTGGTGGAATGCGTAGA-<br>TATCCTGAGGAACACCAG-<br>TGGCGAAGGCGACTCTCTGGGGGCATTCTGACGCTGAGGCG<br>CGAAAGCGTGGGGAGCGAACCGGATTAGATACCCGGG-<br>TAGTC
571264	k__Bacteria, p__Actinobacteria, c__Actinobacteria, o__Actinomycetales, f__Tsukamurellaceae, g__Tsukamurella, s__	CCTACGGGAGGCAGCAGTGGGGAATATTGCACAATGGGCG-<br>GAAGCCTGATGCAGCGAC-<br>GCCGCGTGAGGGATGACGGCCTTCGGGTTGTAAACCTCTTT<br>CACCAGGGACGAAGCGTGAGTGACGGTACCTGGAGAA-<br>GAAGCACCGGCCAACTACGTGCCAG-<br>CAGCCGCGGTAATACGTAGGGTGCGAGCGTTGTCCGGAATT<br>ACTGGGCGTAAAGAGCTCG-<br>TAGGCGGTCTGTCGCGTCGTCTGTGAAAACCCG-<br>CAGCTCAACTGCGGGCTTGCAGGCGATACGGGCATGACTTG<br>AGTACTGTAGGGGAGACTGGAATTCCTGGTGTAGCGGTG-<br>GAATGCGCAGATATCAGGAG-<br>GAACACCGGTGGCGAAGGCGGGTCTCTGGGCAGTAACTGA<br>CGCTGAGGAGCGAAAGCGTGGGTAGCGAACAGGATTAGA-<br>TACCCTGGTAGTCC
565537	k__Bacteria, p__Firmicutes, c__Bacilli, o__Lactobacillales, f__Leuconostocaceae, g__Leuconostoc, s__	CCTACGGGAGGCTGCAG-<br>TAGGGAATCTTCCACAATGGGCGAAAGCCTGATGGAG-<br>CAAC-<br>GCCGCGTGTGTGATGAAGGCTTTCGGGTCGTAAAGCACTGT<br>TGTATGGGAAGAACAGCTAGAATAGGAAATGATTTTAGTTT-<br>GACGGTAC-<br>CATACCAGAAAGGGACGGCTAAATACGTGCCAGCAGCCGC<br>GGTAATACGTATGTCCCGAGCGTTATCCGGAT-<br>TTATTGGGCGTAAAGCGAGCGCAGAC-<br>GGTTTATTAAGTCTGATGTGAAAGCCCGGAGCTCAACTCCG<br>GAATGGCATTGGAAACTGGTTAACTTGAGTGCAG-<br>TAGAGGTAAGTGGAACTCCATGTG-<br>TAGCGGTGGAATGCGTAGATATATGGAAGATCACCAGTGGC<br>GAAAGCGGCTCTCTGGTCTGTAACTGAC-<br>GCTGAGGCTCGAAAGCGTGGGGAGCAAACAG-<br>GATTAGATACCCTGGTAGTCC
563764	k__Bacteria, p__Firmicutes, c__Bacilli, o__Bacillales, f__, g__, s__	CCTACGGGAGGCAGCAGTAGGGAATCTTCCACAATGGAC-<br>GAAAGTCTGATGGAG-<br>CAATGCCGCGTGAGTGAAGAAGGTTTTCGGATCGTAAAACT<br>CTGTTGTGAGGGAAGACAAGTACGAAAGTAACTGCTCG-<br>TACCTTGACGGTACCTCATT-<br>AGAAAGCCACGGCTAACTACGTGCCAGCAGCCGCGGTAAT<br>ACGTAGGTGGCAAGCGTTGTCCGGAATTATTGGGCGTAAA-<br>GCGCGCG-<br>CAGGCGGTCCCTTAAGTCTGATGTGAAATCCCACGGCTCAA

		CCGTGGAAGGTCATTGGAAACTGGGGGACTTGAG-<br>TACAGAAGAGGAAAGCG-<br>GAATTCCAAGTGTAGCGGTGAAATGCGTAGAGATTTGGAGG<br>AACACCAGTGGCGAAGGCGGCTTTCTGGTCTGTAACTGAC-<br>GCTGAGGCGCGAAAGCGTGGG-<br>GAGCAAACAGGATTAGATACCCTGGTAGTC
549954	k__Bacteria, p__Chloroflexi, c__Thermomicrobia, o__JG30-KF-CM45, f__, g__, s__	CCTACGGGAGGCAGCAGCAAGGAATCTTCCGCAATGGGGG-<br>CAACCCTGACGGAGCAAC-<br>GCCGCGTGGGGGATGACGCCCTTCGGGGTGTAAACCCCTTT<br>TCTGCGGGACGAAGGCGGGGGCGACCTCGCTTGACGGTAC-<br>CGGAGGAAGAAGGAC-<br>CGGCTAACTACGTGCCAGCAGCCGCGGTAATACGTAGGGTC<br>CGAGCGTTGTCCGGAGTTACTGGGCGTAAAGCGCGCG-<br>CAGGCGGCGGTGCTGGCCCGGCGTGAAAGCCCCCGGCTCA<br>ACCGGGGAGGGTCGTCGGGGACCGCACCGCTTGAGGGCGG-<br>TAGGGGCTGGTG-<br>GAATGCCTGGTGTAGTGGTGAAATGCGTAGAGATCAGGCGG<br>AACACCTGTGGCGAAGGCGGCCAGCTGGGCCGTCCCTGAC-<br>GCTGAGGCGCGAAGGCGTGGG-<br>GAGCGAACGGGATTAGATACCCCGGTAGTCC
542110	k__Bacteria, p__Actinobacteria, c__Actinobacteria, o__Actinomycetales, f__Promicromonosporaceae, g__Cellulosimicrobium, s__	CCTACGGGAGGCAGCAGTGGGGAA-<br>TATTGCACAATGGGCGAAAGCCTGATGCAGCGAC-<br>GCCGCGTGAGGGATGAAGGCCTTCGGGTTGTAAACCTCTTT<br>CAGCAGGGAAGAAGCGCAAGTGACGGTACCTGCAGAA-<br>GAAGCGCCGGCTAACTACGTGCCAG-<br>CAGCCGCGGTAATACGTAGGGCGCAAGCGTTGTCCGGAATT<br>ATTGGGCGGGGAGGGAGGGGCGTAGGCGGTTT-<br>GTCGCGTCTGGTGG-<br>GAAAACTCGAGGCTCAACCCCCGAGCTTGCATCGGGTACGG<br>GCAGACTAGAGTGCGGTAGGGGAGACTG-<br>GAATTCCTGGGTGTAGCGGTGGAATGCGCAGA-<br>TATCATGAGGAACCACCGATGGGCGAAGCGCAGGTCTCTGG<br>GCCGCAACTGACGCTGAGAGCGAA-<br>GCATGTGAGCGACAGAATAGAATACCTTGTAGTCC
533026	k__Bacteria, p__Proteobacteria, c__Gammaproteobacteria, o__Xanthomonadales, f__Xanthomonadaceae, g__Dokdonella, s__	CCTACGGGAGGCAGCAGTGGGGAATATTGGACAATGGGGG-<br>CAACCCTGATCCAGCCATGCCGCGTGTGTGAAGAAGGCCTT<br>CGGGTTGTAAAGCACTTTTGTTGGGGAAGAAAC-<br>CGGCCGGGTTAATACCCTGGTTGAATGAC-<br>GGTACCCAAAGAATAAGCACCGGCTAACTTCGTGCCAGCA<br>GCCGCGGTAATACGAAGGGTGCAAGCGTTACTCG-<br>GAATTACTGGGCGTAAAGCGTGCG-<br>TAGGCGGTTTGTTAAGTCTGCTGTGAAAGCCCTGGGCTCAA<br>CCTGGGAACTGCAGTGGATACTGGCGAGCTAGAG-<br>TGTGTCAGAGGATGGTG-<br>GAATTCCCGGTGTAGCGGTGAAATGCGTAGAGATCGGGAG<br>GAACACCAGTGGCGAAGGCGGCCATCTGGGACAACAC-<br>TGACGCTGAGGCACGAAAGCGTGGG-<br>GAGCAAAACAGGATTAGATACCCTGGTAGTC
528869	k__Bacteria, p__Firmicutes, c__Bacilli, o__Bacillales, f__Bacillaceae, g__Bacillus, s__	CCTACGGGAGGCAGCAGTAGGGAATCTTCCGCAATGGAC-<br>GAAAGTCTGACGGAACAAC-<br>GCCGCGTGAGTGATGAAGGTTTTCGGATCCTAAAGCTCTGTT

		GATAAGGAAAAACAAGTACCGTTCGAATATGGCGGTAC-<br>CTTGACGGTACCTAACCAGAAA-<br>GCCACGGCTAACTACGTGCCAGCAGCCGCGGTAATACGTAG<br>GTGGCAAGCGTTGTCCGGAATTATTGGGCG-<br>TAAAGGGCTCG-<br>CAGGCGGTTTCTTAAGTCTGATGTGAAAGCCCCCGGCTCAA<br>CCGGGGAGGGTCATTGGAAACTGGAGAACTTGAGTG-<br>CAGAAGAGGAAAGTGGAATTCCAC-<br>GTGTAGCGGTGAAATGCGTAAAGATGTGGAGGAACACCAG<br>TGGCGAGGCGACTCTCTGGTCTGTACTGGCGTTATGAG-<br>GAAACCGTGGGTAACGAACAAGATTAGATACCGGGTGTC
519673	k__Bacteria, p__Firmicutes, c__Bacilli, o__Gemellales, f__, g__, s__	CCTACGGGAGGCAGCAGTAGGGAATCTTCCG-<br>CAATGGGCGAAAGCTTGACGGAGCAAC-<br>GCCGCGTGAGTGATGAAGGTCTTCGGATCGTAAAACTCTGT<br>TATTAGGGAAGAACAAATGTGTAAGTAACTATGCACGTCTT-<br>GACGGTACCTAATCAGAAA-<br>GCCACGGCTAACTACGTGCCAGCAGCCGCGGTAATACGTAG<br>GTGGCAAGCGTTATCCGGAATTATTGGGCGTAAA-<br>GCGCGCG-<br>TAGGCGGTTTTTTAAGTCTGATGTGAAAGCCCACGGCTCAA<br>CCGTGGAGGGTCATTGGAAACTGGAAAACTTGAGTG-<br>CAGAAGAGGAAAGTG-<br>GAATTCCATGTGTAGCGGTGAAATGCGCAGAGATATGGAA<br>GAACACCAGTGGCGAAGGCGGCTCTCTGGTCTG-<br>CAACTGACGCTGAGGCTCGAAAGCATGGG-<br>TAGCGAACAGGATTAGATACCCTGGTAGTC
504674	k__Bacteria, p__Firmicutes, c__Clostridia, o__Clostridiales, f__[Tissierellaceae], g__Anaero-coccus, s__	CCTACGGGAGGCAGCAGTGGGGAATTTTGCACAATGGGG-<br>GAAACCCTGATGCAGCGAC-<br>GCCGCGTGATTTAGAAGGCCTTCGGGTTGTAAAAATCTTTTG<br>TATAGGAAGAAAATGACAGTACTATACGAA-<br>TAAGGTCCGGCTAATTACGTGCCAG-<br>CAGCCGCGGTAATACGTAAGGACCGAGCGTTGTCCGGAATC<br>ATTGGGCGTAAAGGGTACGTAGGCGGCTAGAAAAGTTA-<br>GAAGTCAAAGGCTATAGCTCAAC-<br>TATAGTAAGCTTCTAAAACTATTTAGCTTGAGAGATGGAAG<br>GGAAAGTGGAATTCCTAGTGTAGCGGTGGAATGCGCAGA-<br>TATTAGGAGGAATAC-<br>CGGTGGCGAAGGCGACTTTCTGGCCATTTTCTGACGCTGAG<br>GTACGAAAGCGTGGGTAGCAAACAGGATTAGATACCCTGG-<br>TAGTC
403853	k__Bacteria, p__Actinobacteria, c__Actinobacteria, o__Actinomy-cetales, f__Propionibacteriaceae, g__Propionibacterium, s__acnes	CCTACGGGAGGCAGCAGTGGGGAATATTGCACAATGGGCG-<br>GAAGCCTGATGCAGCAAC-<br>GCCGCGTGCGGGATGACGGCCTTCGGGTTGTAAACCGCTTT<br>CGCCTGTGACGAAGCGTGAGTGACGGTAATGGGTAAAGAA-<br>GCACCGGCTAACTACGTGCCAG-<br>CAGCCGCGGTGATACGTAGGGTGCGAGCGTTGTCCGGATTT<br>ATTGGGCGTAAAGGGCTCGTAGGTGGTTGATCGCGTCG-<br>GAAGTGTAATCTT-<br>GGGGCTTAACCCTGAGCGTGCTTTCGATACGGGTTGACTTG<br>AGGAAGGTAGGGGAGAATGGAATTCCTGGTGGAGCGGTG-

		GAATGCGCAGATATCAGGAG-<br>GAACACCAGTGGCGAAGGCGGTTCTCTGGGCCTTTCCTGAC<br>GCTGAGGAGCGAAAGCGTGGGGAGCGAACAGGCTTAGA-<br>TACCCTGGTAGTC
365828	k__Bacteria, p__Actinobacteria, c__Actinobacteria, o__Actinomycetales, f__Nocardioidaceae, g__, s__	CCTACGGGAGGCAGCAGTGGGGAA-<br>TATTGGACAATGGGCGAAAGCCTGATCCAGCAAC-<br>GCCGCGTGAGGGATGACTGCCTTCGGGTTGTAAACCTCTTTC<br>AGTACCGACGAAGCGAGAGTGACGGTAGGTACAGAAGAA-<br>GCACCGGCCAACTACGTGCCAG-<br>CAGCCGCGGTAATACGTAGGGTGCGAGCGTTGTCCGGAATT<br>ATTGGGCGTAAAGGGCTCGTAGGCGGTTTGTCGCGTCGG-<br>GAG-<br>TGAAAACTCAGGGCTTAACTCTGAGCTTGCTTCCGATACGG<br>GCAGACTAGAGGTATGCAGGGGAGAACGGAATTCCTGGTG-<br>TAGCGGTGAAATGCGCAGA-<br>TATCAGGAGGAACACCGGTGGCGAAGGCGGTTCTCTGGGC<br>ATGACCTGACGCTGAGGAGCGAAAGTGTGGG-<br>GAGCGAACAGGATTAGATACCCTGGTAGTCC
332486	k__Bacteria, p__Firmicutes, c__Bacilli, o__Lactobacillales, f__Lactobacillaceae, g__, s__	CCTACGGGAGGCAGCAGTAGGAAATCTTCCACAATGGAC-<br>GAAAGTCTGATGGAGCAAC-<br>GCCGCGTGAGTGAAGAAGGGTTTCGGCTCGTAAAACTCTGT<br>TGTTAAAGAAGAACATATCTGAGAG-<br>TAACTGTTCAGGTATTGACGGTATTTAACCAGAAA-<br>GCCACGGCTAACTACGTGCCAGCAGCCGCGGTAATACGTAG<br>GTGGCAAGCGTTGTCCGGATTTATTGGGCGTAAA-<br>GCGAGCG-<br>CAGGCGGTTTTTTAAGTCTGATGTGAAAGCCTTCGGCTCAAC<br>CGAAGAAGTGCATCGGAAACTGGGAAACTTGAGTG-<br>CAGAAGAGGACAGTGGAACTCCATGTG-<br>TAGCGGTGAAATGCGTAGATATATGGAAGAACACCAGTGG<br>CGAAGGCGGCTGTCTGGTCTGTAACAGACGCTGAGGCTCG-<br>GAAAGTATGGGTAAAAACCAA-<br>GAATAAATACCCTGGTAAACCC
303239	k__Bacteria, p__Firmicutes, c__Bacilli, o__Lactobacillales, f__Leuconostocaceae, g__Leuconostoc, s__	CCTACGGGAGGCTGCAG-<br>TAGGGAATCTTCCACAATGGGCGAAAGCCTGATGGAG-<br>CAAC-<br>GCCGCGTGTGTGATGAAGGCTTTCGGGTCGTAAAGCACTGT<br>TGTATGGGAAGAACAACTAGAGTAGGGAATGACTTTAG-<br>TTTGACGGTAC-<br>CATACCAGAAAGGGACGGCTAAATACGTGCCAGCAGCCGC<br>GGTAATACGTATGTCCCGAGCGTTATCCGGAT-<br>TTATTGGGCGTAAGGCGAGCGCAGACGGTT-<br>GATTAAGTCTGATGTGAAAGCCCGGAGCTCAACTCCGGAAA<br>GGCATTGGAAACTGGTCAACTTGAGTGCAG-<br>TAGAGGTAAGTGGAACTCCATGTGTAGCGGTG-<br>GAATGCGTAGATATATGGAAGAACACCAGCGGCGAAGGCG<br>GCTTACTGGACTGTAACTGACGTT-<br>GAGGCTCGAAAGTGTGGGTAGCAAACAGGATTAGA-<br>TACCCTGGTAGTC

301554	k__Bacteria, p__Actinobacteria, c__Actinobacteria, o__Actinomycetales, f__Nocardioidaceae, g__, s__	CCTACGGGAGGCAGCAGTGGGGAA-<br>TATTGGACAATGGGCGAAAGCCTGATCCAGCAAC-<br>GCCGCGTGAGGGATGACTGCCTTCGGGTTGTAAACCTCTTTC<br>AGTAGGGACGAAGCGCAAGTGACGGTACCTACAGAAGAA-<br>GCACCGGCCAACTACGTGCCAG-<br>CAGCCGCGGTAATACGTAGGGTGCGAGCGTTGTCCGGAATT<br>ATTGGGCGTAAAGGGCTCGTAGGCGGTTTGTCACGTCGG-<br>GAGTGAAAAC-<br>CAGGTGCTTAACACCTGGCCTGCTTCCGATACGGGCAGACT<br>AGAGGTATGCAGGGGAGAACGGAATTCCTGGTG-<br>TAGCGGTGAAATGCGCAGATATCAGGAG-<br>GAACACCGGTGGCGAAGGCGGTTCTCTGGGCATTACCTGAC<br>GCTGAGGAGCGAAAGTGTGGGGAGCGAACAGGATTAGA-<br>TACCCTGGTAGTC
291846	k__Bacteria, p__Actinobacteria, c__Actinobacteria, o__Actinomycetales, f__Corynebacteriaceae, g__Corynebacterium, s__	CCTACGGGAGGCAGCAGTGGGGAATCTT-<br>GCACAATGGGCGAAAGCCTGATGCAGCGAC-<br>GCCGCGTGGGGGATGACGGCCTTCGGGTTGTAAACCCCTTT<br>CGCTAGGGAAGAAGCCTTTCGGGGTGACGGTACCTGGA-<br>TAAGAAGCACCGGCTAACTAC-<br>GTGCCAGCAGCCGCGGTAATACGTAGGGTGCAAGCGTTGTC<br>CGGAATTACTGGGCGTAAAGAGCTCGTAGGTGGTTT-<br>GTCGCGTCGTCTGTGAAATCCCGGGGCTTAACTCCGGGTCTG<br>CAGGCGATACGGGCATAACTAGAGTGCTGTAGGGGA-<br>GACTGGAATTCCTGGTG-<br>TAGCGGTGAAATGCGCAGATATCAGGAGGAACACCGATGG<br>CGAAGGCAGGTCTCTGGGCAGTAACTGACGCTGAG-<br>GAGCGAAAGCATGGGGAG-<br>CAAACAGAATTAGATACCCTGGTAGTC
276168	k__Bacteria, p__Proteobacteria, c__Gammaproteobacteria, o__Pseudomonadales, f__Pseudomonadaceae, g__, s__	CCTTACGGGAGGCGAGCCAGGGGGAATATTT-<br>GGACAATGGGGGAAAC-<br>CTGATCCAGCCATCCCGGCGTTTGTGAAGAAGGTCTTCGGA<br>TTGTAAAGCACTTTAAGTTGGGAGGAAGGGCATTAAC-<br>CTAATACGTTAGTGTTTTGAC-<br>GTTACCGACAGAATAAGCACCGGCTAACTTCGTGCCAGCAG<br>CCGCGGTAATACGAAGGGTGCAAGCGTTAATCG-<br>GAATTACTGGGCGTAAAGCGCGCG-<br>TAGGTGGTTCGTTAAGTTGGATGTGAAAGCCCCGGGCTCAA<br>CCTGGGAACTGCATCCAAAACTGGCGAGCTAGAGTACGG-<br>TAGAGGGTGGTG-<br>GAATTTCCTGTGTAGCGGTGAAATGCGTAGATATAGGAAGG<br>AACACCAGTGGCGAAGGCGACCACCTGGACTGA-<br>TACTGACACTGAGGTGCGAAAGCGTGGG-<br>GAGCAAACAGGATTAGATACCCTGGTAGTC
268598	k__Bacteria, p__Bacteroidetes, c__[Saprospirae], o__[Saprospirales], f__Saprospiraceae, g__, s__	CCTACGGGAGGCAGCAGTAAGGAATATTGGTCAATGGAC-<br>GAAAGTCTGAAC-<br>CAGCCATCCCGCGTGCAGGATGAAGGTCCTATGGATTGTAA<br>ACTGCTTTTAGCAGAGAAGAAAA-<br>GCGTCCTTTTAAGGGTGTCTGACGGTATCTGCGGAATAA-<br>GCACCGGCTAACTCCGTGCCAGCAGCCGCGGTAATACGGA<br>GGGTGCAAGCGTTATCCGGAATCAC-

		TGGGTTTAAAGGGTGCGTAGGCGGAATATTAAGTCAG-<br>TGGTTGGAAAACCCTTTCGGCCTTAACCGAATAGAACTGCC<br>ATTGATACTGATATTCTTGAATTGGGTTGAGGTTAGCG-<br>GAATGTGACATGTAGCGGTGAAATGCATAGATATGTCATGG<br>AACACCAATTGCGAAGGCAGCTAGCTGGGCCTTGATTGAC-<br>GCTGAGGCACGAAAGCGTGGG-<br>TAACGAACAGGATTAGATACCCTGGTAGTC
257531	k__Bacteria, p__Actinobacteria, c__Thermoleophilia, o__Gaiellales, f__Gaiellaceae, g__, s__	CCTACGGGAGGCAGCAGTAGGGAATCTTGCG-<br>CAATGGGCGAAAGCCTGACGCAGCGAC-<br>GCCGCGTGCGGGAAGAAGGCCTTCGGGTTGTAAACCGCTTT<br>CAGGAGGGACGAAGCCAC-<br>TCGGGTTAACAGCCCAGAGGGTGACGGTACCTCCAGAA-<br>GAA-<br>GCCCCGGCTAACTACGTGCCAGCAGCCGCGGTAATACGTAG<br>GGGGCAAGCGTTGTCCGGATTCATTGGGCGTAAA-<br>GAGCGTG-<br>TAGGCGGCCAGGTAGGTCCGTTGTGAAAACTCGAGGCTCAA<br>CCTCGAGACGTCGATGGAAACCATCTGGCTAGAGTCCG-<br>GAAGAGGAGAGTG-<br>GAATTCCTGGTGTAGCGGTGAAATGCGCAGATATCAGGAAG<br>AACACCTATGGCGAAGGCAGCTCTCTGGGACGGTACTGAC-<br>GCTGAGACGCGAAAGCGTGGG-<br>GAGCGAACAGGATTAGATACCCTGGTAGTCC
255576	k__Bacteria, p__Proteobacteria, c__Gammaproteobacteria, o__Xanthomonadales, f__Sinobacteraceae, g__, s__	CCTACGGGAGGCAGCAGTGGGGAA-<br>TATTGGACAATGGGCGAAAGCCTGATCCAG-<br>CAATGCCGCGTGTGTGAAGAAGGCCTTCGGGTTGTAAAGCA<br>CTTTAGGTCGGAAAGAAAAAGCTTTGGTGAATATCCAAA-<br>GCCTTGACGGTACCGACAGAA-<br>TAAGCACCGGCTAACTCTGTGCCAGCAGCCGCGGTAATACA<br>GAGGGTGCTAGCGTTAATCGGATTTACTGGGCGTAAA-<br>GCGTGCGTAGGTGGCTAC-<br>GAAAGTCGGATGTGAAAGCCCCGGGCTTAACCTGGGAATG<br>CCATTCGATACTTCGTGACTAGAATTCGGTAGAGGGAGGCG-<br>GAACTCCAGGTG-<br>TAGCGGTGAAATGCGTAGATATCTGGAAGAACACCGATGG<br>CGAAGGCAACCTCCTGGCCTGAATTGACACTGAGGCAC-<br>GAAAGCGTGGGGAGCAAACAG-<br>GATTAGATACCCTGGTAGTC
246939	k__Bacteria, p__Actinobacteria, c__Actinobacteria, o__Actinomycetales, f__, g__, s__	CCTACGGGAGGCAGCAGTGGGGAATCTTGCGCAATGGGCG-<br>GAAGCCTGACGCAGCGAC-<br>GCCGCGTGGGGGATGACGGCCTTCGGGTTGTAAACCTCTTT<br>CACCATTGGCGAAGGCCAGCATTTTGTGTTGGTTGACGG-<br>TAGGTGGGGAAGAA-<br>GCGCCGGCTAACTACGTGCCAGCAGCCGCGGTAATACGTAG<br>GGCGCAAGCGTTGTCCGGATTTATTGGGCGTAAAGAGCTCG-<br>TAGGTGGTTT-<br>GTCGCGTCTGTCGTGAAAGCCCATGGCTTAACTGTGGGTTTG<br>CGGTGGATACGGGCTGACTAGAGGCAGGTAGGGGAGAGTG-<br>GAATTCCCGGTG-<br>TAGCGGTGAAATGCGCAGATATCGGGAGGAACACCGGTGG

		CGAAGGCGGCTCTCTGGGCCTGTCCTGACGCTGAG-<br>GAGCGAAAGCGTGGGTAGCGAACAG-<br>GATTAGATACCCTGGTAGTC
244132	k__Bacteria, p__Proteobacteria, c__Alphaproteobacteria, o__Rhizobiales, f__Brucellaceae, g__Ochrobactrum, s__	CCTACGGGAGGCAGCAGTGGGGAATATTGGACAATGGGCG-<br>CAA-<br>GCCTGATCCAGCCATGCCGCGTGAGTGATGAAGGCCCTAGG<br>GTTGTAAAGCTCTTTCACCGGTGAAGATAATGACGGTAAC-<br>CGGAGAAGAA-<br>GCCCCGGCTAACTTCGTGCCAGCAGCCGCGGTAATACGAAG<br>GGGGCTAGCGTTGTTCGGATTTACTGGGCGTAAAGCGCAC-<br>GTAGGCG-<br>GACTTTTAAGTCAGGGGTGAAATCCCGGGGCTCAACCCCGG<br>AACTGCCTTTGATACTGGAAGTCTTGAGTATGGAA-<br>GAGGTGAGTGGAATTCCGAGTG-<br>TAGAGGTGAAATTCGTAGATATTCGGAGGAACACCAGTGGC<br>GAAGGCGGCTCACTGGACCATTACTGAC-<br>GCTGAGGTGCGAAAGCGTGGGGAGCAAACAG-<br>GATTAGATACCCTGGTAGTC
243177	k__Bacteria, p__[Thermi], c__Deinococci, o__Deinococcales, f__Trueperaceae, g__, s__	CCTACGGGAGGCAGCAGTTAG-<br>GAATCTTCCCCAATGGGCGAAAGCCTGAGGGAGCGACAC-<br>TGCGTGAAGGATGACGGCCTTCGGGTTGTAAACTTCTGAAC<br>GAGGGACGAATACTGACGGTACCTCGCTAACAGCAC-<br>CGGCTAACTCCGTGCCAG-<br>CAGCCGCGGTAATACGGAGGGTGCAAGCGTTATCCGGAATC<br>ACTGGGCGTAAAGGGCGCGTAGGCGGTTTACT-<br>AAGTCCGATGTTAAAGAC-<br>CGGGGCTCAACCCCGATATGGCGTTGGATACTGGTAGGCTT<br>GACGACTGGAGAGGAAAGTAGAATTACCAGTGTAGCGGTG-<br>GAATGCGTAGATACTGGTAG-<br>GAATACCCATTGCGAAGGCAGCTTTCTGGACAGTCCGTGAC<br>GCTGAGGCGCGAAAGTGTGGGGAGCAAACCGGATTAGA-<br>TACCCGGGTAGTCC
235293	k__Bacteria, p__Actinobacteria, c__Thermoleophilia, o__Solirubrobacterales, f__, g__, s__	CCTACGGGAGGCAGCAGTGGGGAATCTTGCG-<br>CAATGCGCGAAAGCGTGACGCAGCAAC-<br>GCCGCGTGAGGGAAGAAGGCCTTCGGGTTGTAAACCTCTTT<br>CAGGAGGGACGAAGGTTCAGGCGCGAATAGCGTCCTG-<br>GACTGACGGTACCTCCAGAAGAA-<br>GCCCCGGCTAACTACGTGCCAGCAGCCGCGGTAATACGTAG<br>GGGGCTAGCGTTGTCCGGAATCATTGGGCGTAAA-<br>GCGCGCGTAGGCGGCCCGG-<br>TAAGTCCGCTGTAAAAGTCAAAGGCTCAACCTTTGAATGTC<br>AGTGGATACTGCCGGGCTAGAGTCCGGAAGAGGCGAGTG-<br>GAATTCCTGGTGTAGCGGTG-<br>GAATGCGCAGATATCAGGAGGAACACCAACGGCGAAGGCA<br>GCTCGCTAGGACGGTACTGACGCTAAGGCGCGAAA-<br>GCGTGGGGAGCGAACAGGATTAGATACCCTGGTAGTCC
234307	k__Bacteria, p__TM7, c__TM7-1, o__, f__, g__, s__	CCTACGGGAGGCAGCAGTAGGGAATTTTCCACAATGGAC-<br>GAAAGTCTGATGGAGCAAC-<br>GCCGCGTGCAGGATGAATGCCTTCGGGTTGTAAACTGCTTTT<br>ATCTGTGACGAATATGACGGTAGCAGATGAA-

		TAAGGATCGGCTAACTCCGTGCCAG-<br>CAGCCGCGGTCATACGGAGGATCCAAGCGTTATCCGGAATT<br>ACTGGGCGTAAAGAGTTGCGTAGGTGGCAGAGTATGTCTG-<br>TAGTGAAAGCGTGTGGCTCAAC-<br>CATATACACATTTACAGAAACTGCTCAGCTAGAGGGCGAGA<br>GAGGTTACTAGAATTCCTAGTGTAGGAGTGAAATCCG-<br>TAGATATTAGGAGGAATAC-<br>CGATGGCGTAGGCAGGTAACTGGCTCGTCCCTGACACTAA<br>GCACGAAAGCGTGGGTAGCAAACGGGATTAGATACCCCGG-<br>TAGTCC
225335	k__Bacteria, p__Bacteroidetes, c__Bacteroidia, o__Bacteroidales, f__Prevotellaceae, g__Prevotella, s__	CCTACGGGAGGCAGCAGTGAGGAA-<br>TATTGGTCAATGGGCGAGAGCCTGAAC-<br>CAGCCAAGTAGCGTGCAGGACGACGGCCCTATGGGTTGTAA<br>ACTGCTTTTATGCGGGGATAAAACGCGGGAC-<br>GTGTCCCGCCTTGCAGGTACCGCATGAA-<br>TAAGGACCGGCTAATTCCGTTCCAGCAGCCGCGGTAATACG<br>GAAGGTCCGGGCGTTATCCGGAT-<br>TTATTGGGTTTAAAGGGAGCGTAGGCCGCTTTGTAA-<br>GCGTGTTGTGAAATTTGCCGGCTCAACCGGTAACTTGCAGC<br>GCGAACTGCAGAGCTTGAGTGTGCGCAACGTTGGCG-<br>GAATTCGTCGTG-<br>TAGCGGTGAAATGCTTAGATATGACGAAGAACTCCGATTGC<br>GAAGGCAGCTGACGGGAGCACTACTGACGCTGAA-<br>GCTCGAAAGTGCGGGTATCGAACAG-<br>GATTAGATACCCTGGTAGTC
225201	k__Bacteria, p__Acidobacteria, c__Acidobacteriia, o__Acidobacteriales, f__Koribacteraceae, g__, s__	CCTACGCGGAGGCAGCAGTGGGGAATTTTGCGCAATGGGG-<br>GAAACCCTGACGCAGCAAC-<br>GCCGCGTGGAGGATGAAGTCCCTTGGGACGTAAACTCCTTT<br>CGATCGGGACGATAATGACGGTACCGGAAGAAGAA-<br>GCCCCGGCTAACTTCGTGCCAG-<br>CAGCCGCGGTAATACGAGGGGGGCAAGCGTTGTTCGGAATT<br>ATTGGGCGTAAAGGGCGCGTATGCGGTGCGG-<br>TAAGTCTTCTGTGAAATCTCCGGGCTCAACTCGGAGCCTGC<br>AGAGGAAACTGCCGTGCTGGAGTGTGGGAGAGGAGAGTG-<br>GAATTCCCGGTG-<br>TAGCGGTGAAATGCGTAGATATCGGGAGGAACACCCGTGG<br>CGAAAGCGGCTCTCTGGACCACTACTGAC-<br>GCTGAGGCGCGAAAGCTAGGGGAGCAAACAG-<br>GATTAGATACCCTGGTAGTCC
224670	k__Bacteria, p__Proteobacteria, c__Gammaproteobacteria, o__Enterobacteriales, f__Enterobacteriaceae, g__Serratia, s__marcescens	CCTACGGGAGGCAGCAGTGGGGAATATTGCACAATGGGCG-<br>CAA-<br>GCCTGATGCAGCCATGCCGCGTGTGTGAAGAAGGCCTTCGG<br>GTTGTAAAGCACTTTCAGCGAGGAG-<br>GAAGGTGGTGAGCTTAATACGCTCATCAATTGAC-<br>GTTACTCGCAGAAGAAGCACCGGCTAACTCCGTGCCAGCAG<br>CCGCGGTAATACGGAGGGTGCAAGCGTTAATCG-<br>GAATTACTGGGCGTAAAGCGCAC-<br>GCAGGCGGTTTGTTAAGTCAGATGTGAAATCCCCGGGCTCA<br>ACCTGGGAACTGCATTTGAAACTGGCAAGCTAGAGTCTCG-<br>TAGAGGGGGG-<br>TAGAATTCCAGGTGTAGCGGTGAAATGCGTAGAGATCTGGA

		GGAATACCGGTGGCGAAGGCGGCCTCTTGCACGAAA-GACTGACGCTCAGGTCAGAAA-GCGTGGGGAAGCAAACAGGGATTAGATACCCTGGTAGTCC
216345	k__Bacteria, p__Actinobacteria, c__Actinobacteria, o__, f__, g__, s__	CCTACGGGAGGCAGCAGTGGGGAATATTGCG-CAATGGGCGAAAGCCTGACGCAGCGAC-GCCGCGTGAGGGATGAAGGCCTTCGGGTTGTAAACCTCTTTCAGCAGGGACGAAAACAGACGGTACCTGCAGAAGAA-GCACCGGCCAACTACGTGCCAG-CAGCCGCGGTACTACGTAGGGTGCAAGCGTTGTCCGGAATTATTGGGCGTAAAGAGCTCG-TAGGCGGTCTGTCGCGTCGGCTGTGAAAACTCGGGGCTCAACCCCGAGCCTGCAGTCGATACGGGCAGACTAGAGTGCGG-TAGGGGAGACTG-GAATTCCTGGTGTAGCGGTGAAATGCGCAGATATCAGGAGGAACACCGGTGGCGAAGGCGGGACTCTGGGCCGATATTGAC-GCTGAGGAGCGAAAGCGTGGG-GAGCGAACAGGATTAGATACCCTGGTAGTC
212239	k__Bacteria, p__Acidobacteria, c__Acidobacteriia, o__Acidobacteriales, f__Acidobacteriaceae, g__Terriglobus, s__	CCTACGGGAGGCAGCAGTGGGGAATTTTGCGCAATGGGG-GAAACCCTGACGCAGCAAC-GCCGCGTGGAGGATGAAGTCTCTTGGGACGTAAACTCCTTTCGATAGGGAAGATAATGACGGTACCTATAGAAGAA-GCCCCGGCTAACTTCGTGCCAG-CAGCCGCGGTAATACGAGGGGGGCAAGCGTTGTTCGGAATTATTGGGCGTAAAGGGCGCGTAGGCGGTTTGG-CAAGTTTCGTGTGAAATCTTCGGGCTCAACTCGAAGTCTGCACGGAAAACTGCCGGGCTTGAGTATGGGAGAGGTGAGTG-GAATTTCCGGTGTAGCGGTG-GAATGCGTAGATATCGGAAGGAACACCTGTGGCGAAAGCGGCTCACTGGACCATTACTGACGCTGAGGCGCGAAA-GCTAGGGGAGCAAACAGGATTAGATACCCTGGTAGTC
211222	k__Bacteria, p__Actinobacteria, c__Actinobacteria, o__Actinomycetales, f__Nocardioidaceae, g__, s__	CCTACGGGAGGCAGCAGTGGGGAATATTGGACAATGGGCG-GAAGCCTGATCCAGCAAC-GCCGCGTGAGGGATGACGGCCTTCGGGTTGTAAACCTCTTTCAGCACCGACGAAGCTCCGCTTTTTGGTGGTTTGACGG-TAGGTGCAGAAGAAGGAC-CGGCCAACTACGTGCCAGCAGCCGCGGTAATACGTAGGGTCCGAGCGTTGTCCGGAATTATTGGGCGTAAAGGGCTCG-TAGGCGGTTTGTCGCGTCGGGAG-TGAAAACATCTGGCTTAACTGGGTGCTTGCTTTCGATACGGGCAGACTGGAGGTATGCAGGGGAGAACGGAATTCCTGGTG-TAGCGGTGAAATGCGCAGA-TATCAGGAGGAACACCGGTGGCGAAGGCGGTTCTCTGGGCATGACCTGACGCTGAGGAGCGAAAGTGTGGG-GAGCGAACAGGATTAGATACCCTGGTAGTC
201979	k__Bacteria, p__Proteobacteria, c__Betaproteobacteria, o__Burkholderiales, f__Alcaligenaceae, g__Achromobacter, s__	CCTACGGGAGGCAGCAGTGGGGAATTTTGGACAATGGGG-GAAACCCTGATCCAGCCATCCCGCGTGTGCGATGAAGGCCTTCGGGTTGTAAAGCACTTTTGGCAGGAAAGAAAC-GTCATGGGTTAA-TACCCCGTGAAACTGACGGTACCTGCAGAATAAGCACCGGCTAACTACGTGCCAGCAGCCGCGGTAATACGTAGGGTG-

		CAAGCGTTAATCGGAA-<br>TACTGGGCGTAAGCGTGCGCAGGCGGTCGGAAAGAAAGAT<br>GTGAAATCCCAAGCTTACTTTGAACTGCATTTTTAACTAC-<br>CAGCTAAAGTGTGTCAAAGGGAGGTGGATTCCCCGTGTACC<br>AATGAAATGCCTAATTTCGGAGGACCCGGTGGCAAGCAC-<br>CTCTGGGATACACTGAC-<br>CTATGCCAAACTGGGACAACAGATTAATCCTGTATCC
200629	k__Bacteria, p__Actinobacteria, c__Thermoleophilia, o__Solirubrobacterales, f__, g__, s__	CCTACGGGAGGCAGCAGTGGGGAATCTTGCG-<br>CAATGCGCGAAAGCGTGACGCAGCAAC-<br>GCCGCGTGGGGGAAGACGGCCTTCGGGTTGTAAACCCCTTT<br>CAGGAGGGACGAAGGCTCACCGGTGAATAGCCGTGTGAG-<br>TTGACGGTACCTCCACAAGAA-<br>GCTCCGGCTAACTACGTGCCAGCAGCCGCGGTAATACGTAG<br>GGAGCAAGCGTTGTCCGGAATCATTGGGCGTAAA-<br>GCGCGCG-<br>TAGGTGGCTTCATAAGTCCGCTGTGAAAGTCAAAGGCTCAA<br>CCTTTGAAAGCCGGTGGATACTGTGGGGCTAGAGTAC-<br>CGAAGAGGCGAGTG-<br>GAATTCCTGGTGTAGCGGTGGAATGCGCAGATATCAGGAGG<br>AACACCAATAGCGAAGGCAGCTCGCTGGAC-<br>GTTACTGACACTGAGGCGCGAAAGCGTGGG-<br>GAGCGAACAGGATTAGATACCCCGGTAGTCC
172410	k__Bacteria, p__Proteobacteria, c__Gammaproteobacteria, o__Xanthomonadales, f__Xanthomonadaceae, g__, s__	CCTACGGGAGGCAGCAGTGGGGAATATTGGACAATGGGCG-<br>CAAGCCTGATCCAG-<br>CAATGCCGCGTGTGTGAAGAAGGCCTTCGGGTTGTAAAGCA<br>CTTTTATCGGGAGCGAAATACCATTGGCTAATAACCAG-<br>TGGGGCTGACGGTACCCGAGGAA-<br>TAAGCACCGGCTAACTTCGTGCCAGCAGCCGCGGTAATACG<br>AAGGTGCAAGCGTTAATCGGAATTACTGGGCG-<br>TAAAGGGTGCGTAGGCGGTTT-<br>GTTAAGTCTGTCGTGAAATCCCCGGGCTCAACCTGGGAATG<br>GCGATGGATACTGGCAAGCTAGAGTGTGTCAGAG-<br>GATGGTGGAATTTCCGGTG-<br>TAGCGGTGAAATGCGTAGAGATCGGAAGGAACATCAGTGG<br>CGAAGGCGGCCATCTGGGACAACACTGACGCTGAAGCAC-<br>GAAAGCGTGGGGAGCAAACAG-<br>GATTAGATACCCTGGTAGTCC
170244	k__Bacteria, p__Firmicutes, c__Clostridia, o__Clostridiales, f__Clostridiaceae, g__Clostridium, s__intestinale	CCTACGGGAGGCAGCAGTGGGGAATATTGCACAATGGGG-<br>GAAACCCTGATGCAGCAAC-<br>GCCGCGTGAGTGATGAAGGCCTTCGGGTTGTAAAGCTCTGT<br>CTTCAGGGACGATAATGACGGTACCTGAGGAGGAAGCCAC-<br>GGCTAACTACGTGCCAG-<br>CAGCCGCGGTAATACGTAGGTGGCAAGCGTTGTCCGGATTT<br>ACTGGGCGTAAAGGGAGCGTAGGCGGATACTTAAGTGG-<br>GATGTGAAATAC-<br>CTGGGCTTAACTTGGGTGCTGCATTCCAAACTGGGTGTCTAG<br>AGTGTTGGAGAGGAAAGTGGAATTCCTAGTG-<br>TAGCGGTGAAATGCGTAGAGATTAGGAA-<br>GAACACCAGTGGCGAAGGCGACTTTCTGGACAATAACTGA<br>CGCTGAGGCTCGAAAGCGTGGGGAGCAAACAGGATTAGA-<br>TACCCTGGTAGTC

142506	k__Bacteria, p__Actinobacteria, c__Actinobacteria, o__Actinomycetales, f__, g__, s__	CCTACGGGAGGCAGCAGTGGGGAATATTGCG-<br>CAATGGGCGAAAGCCTGACGCAGCGAC-<br>GCCGCGTGAGGGATGACGGCCTTCGGGTTGTAAACCTCTTT<br>CAGCAGGGACGAAGCGAAAGTGACGGTACCTGCAGAA-<br>GAAGCACCGGCCAACTACGTGCCAG-<br>CAGCCGCGGTAATACGTAGGGTGCAAGCGTTGTCCGGAATT<br>ATTGGGCGTAAA-<br>GAGCTCCTAGGCGGTCTGTCGCGTCGGCTGTGAAACCCCGA<br>GGCACAACCTCGGGCCTGCAGTCGATACGGGCAGACTA-<br>GAGTTCGGTAGGGGAGACTT-<br>GGAATTCCTGGTGTTGCGGTGAAATGCGCAGTTATCAGAGA<br>ACACCGGTGGCGAAGGCGGTCTCTGGCCGAAACTGAC-<br>GCTGAGAGCGAAAGCGAGGGGAG-<br>CAACAGATTAGATACCCTGGTAGTC
134121	k__Bacteria, p__Actinobacteria, c__Actinobacteria, o__Actinomycetales, f__Microbacteriaceae, g__, s__	CCTACGGGAGGCAGCAGTGGGGAATATTGCACAATGGGCG-<br>CAAGCCTGATGCAGCAAC-<br>GCCGCGTGAGGGACGAAGGCCTTCGGGTTGTAAACCTCTTT<br>TAGTAGGGAAGAAGCGAAAGTGACGGTACCTG-<br>CAGAAAAAGCACCGGCTAACTACGTGCCAG-<br>CAGCCGCGGTAATACGTAGGGTGCAAGCGTTATCCGGAATT<br>ATTGGGCGTAAAGAGCTCGTAGGCGGTTT-<br>GTCGCGTCTGCTGTGAAAACTGGGGGCTCAACCCCCAGCCT<br>GCAGTGGGTACGGGCAGACTAGAGTGCGGTAGGGGA-<br>GATTGGAATTCCTGGTGTAGCGGTG-<br>GAATGCGCAGATATCAGGAGGAACACCGATGGCGAAGGCA<br>GATCTCTGGGCCGTAACTGACGCTGAGGAGCGAAA-<br>GCATGGGGAGCGAACAGGATTAGATACCCTGGTAGTCC
132112	k__Bacteria, p__Firmicutes, c__Bacilli, o__Bacillales, f__Bacillaceae, g__Bacillus, s__	CCTACCGGAGGGAACAGTAGGGAATCCTCCCCAATGGAC-<br>CAAAGTCTGAC-<br>CGAACAACCCCCCCTGAGTGATGAAAGTTTTCCGATCCTAA<br>AACTCTGTTGTTAGGGAAAAACAAGTACCGTTCCAA-<br>TAAGGGGGGACCTTGACCGGAC-<br>CTAACCCAAAAGCCCCCGCTAACTACCTGACCACCGCCCCG<br>GTAATACCTAAGTGGCAAGCGTTGTCCCGAATTATTGGGCG-<br>TAAAGCGCGCG-<br>CAGGCGGTTTTTTAAGTTTGATGTGAAAGCCCCCGGCTCAAC<br>CGGGGAGGGTCATTGGAAACTGGGGAACTTGAGTGCAGAA-<br>GAGGAGAGTGGAATTCCACGTG-<br>TAGCGGTGAAATGCGTAGAGATGTGGAGGAACACCAGTGG<br>CGAAGGCGACTCTCTGGTCTGTAACTGAC-<br>GCTGAGGCGCGAAAGCGTGGGGAGCGAACAG-<br>GATTAGATACCCTGGTAGTCC
115045	k__Bacteria, p__Actinobacteria, c__Actinobacteria, o__Actinomycetales, f__Pseudonocardiaceae, g__Amycolatopsis, s__	CCTACGGGAGGCAGCAGTGGGGAATATTGCACAATGGGCG-<br>CAAGCCTGATGCAGCGAC-<br>GCCGCGTGAGGGATGACGGCCTTCGGGTTGTAAACCTCTTT<br>CGCCAGGGACGAAGCGCAAGTGACGGTACCTGGATAA-<br>GAAGCACCGGCTAACTACGTGCCAG-<br>CAGCCGCGGTAATACGTAGGGTGCGAGCGTTGTCCGGAATT<br>ATTGGGCGTAAAGAGCTCGTAGGCGGTTT-<br>GTCGCGTCGGCCGTGAAATCTCCACGCTTAAC-<br>GTGGAGCGTGCGGTCGATACGGGCAGACTTGAGTTCGGCAG

		GGGAGACTGGAATTCCTGGTGTAGCGGTGAAATGCGCAGA-<br>TATCAGGAGGAACAC-<br>CGGTGGCGAAGGCGGGTCTCTGGGCCGATACTGACGCTGAG<br>GAGCGAAAGCGTGGGGAGCGAACAGGATTAGA-<br>TACCCTGGTAGTCC
71833	k__Bacteria, p__Firmicutes, c__Bacilli, o__Bacillales, f__Paenibacillaceae, g__, s__	CCTACGGGAGGCAGCAG-<br>TAGGGAATTTTCCACAATGGGCGAAAGCCTGATGGAG-<br>CAAC-<br>GCCGCGTGAACGATGAAGGTCTTCGGATTGTAAAGTTCTGT<br>TGTCAGGGACGAATAAGTACCGTTCGAACAGGGCGGTAC-<br>CTTGACGGTACCTGACGA-<br>GADDGCCCCGGCTAACTACGTGCCAGCAGCCGCGGTAATA<br>CGTAGGGGGCAAGCGTTGTCCGGAATTATTGGGCGTAAA-<br>GCGCGCGCAGGCGGTCGGG-<br>TAAGTCTGATGTTAAAGCCCGGGGCTCAACCCCGGTTCGCA<br>TTGGAAACTGCTCGACTTGAGTGCAGAAGAGGAAAGCG-<br>GAATTCCACGTG-<br>TAGCGGTGAAATGCGTAGAGATGTGGAGGAACACCAGTGG<br>CGAAGGCGGCTTTCTGGTCTGTAACTGAC-<br>GCTGAGGCGCGAAAGCGTGGGGAGCAAACAG-<br>GATTAGATACCCTGGTAGTC
37034	k__Bacteria, p__Bacteroidetes, c__[Saprospirae], o__[Saprospirales], f__Chitinophagaceae, g__, s__	CCTACGGGAGGCAGCAGTAAGGAATATTGGTCAATGGAC-<br>GCAAGTCTGAAC-<br>CAGCCATGCCGCGTGAAGGATTAAGGCCCTCAGGGTTGTAA<br>ACTTCTTTTATCTGGGAAGAAACTCTCGCATTCTTGCGA-<br>GATTGACGGTACCAGTTGAATAA-<br>GCACCGGCTAACTCCGTGCCGGCAGCCGCGGTAATACGGA<br>GGGTGCAAGCGTTATCCGGAT-<br>TTACTGGGTTTAAAGGGTGCGTAGGCGGG-<br>CAGGTAAGTCTGTGGTGAAATCTCCGAGCTCAACTCGGAAA<br>TTGCCATGGATACTATTTGTCTTGAATGTCGTG-<br>GAGGTGAGCGGAATATGG-<br>CATGTAGCGGTGAAATGCTTAGATATGCCATGGAACACCAA<br>TTGCGAAGGCAGCTCGCTACACGATGATTGAC-<br>GGTGATGCACGAAAGCGTGGG-<br>TAGCGAACAGGATTAGATACCCTGGTAGTC
11622	k__Bacteria, p__Actinobacteria, c__Actinobacteria, o__Actinomycetales, f__Frankiaceae, g__, s__	TCCTACGGGAGGCGGCAGTGGGGAA-<br>TATTGCACAATGGGCGAAAGCCTGATGCAGCGAC-<br>GCCGCGTGAGGGACGACGGCCTTCGGGTTGTAAACCTCTTT<br>CAGCTCCGACGAAGCGAAAGTGACGGTAGGAGCAGAA-<br>GAAGCACCGGCCAACTACGTGCCAG-<br>CAGCCGCGGTAATACGTAGGGTGCAAGCGTTGTCCGGAATT<br>ATTGGGCGTAAAGAGCTCGTAGGCGGCTT-<br>GTCGCGTCGGCTGTGAAAAC-<br>CTGGGGCTCAACCCCGGGCCTGCAGCCGATACGGGCAAGCT<br>AGAATTCGGTAGGGGAGACTGGAATTCCTGGTG-<br>TAGCGGTGAAATGCGCAGATATCAGGAG-<br>GAACACCGGTGGCGAAGGCGGGTCTCTGGGCCGATATTGAC<br>GCTGAGGAGCGAAAGCGTGGGGAGCAAACAGGATTAGA-<br>TACCCTGGTAGTCCA

1710148	k__Bacteria, p__Firmicutes, c__Bacilli, o__Bacillales, f__Bacillaceae, g__Bacillus, s__	CCTACGGGAGGCAGCAGTAGGGAATCTTCCGCAATGGAC-<br>GAAAGTCTGACGGAGCAAC-<br>GCCGCGTGAGTGATGAAGGTTTTCGGATCGTAAAGCTCTGTT<br>GTTAGGGAAGAACAAGTACTCGTTCGAATAGGGCGGTAC-<br>CTTGACGGTACCTAAACAGAAA-<br>GCCACGGATTAACTACGTGCCAGCAGCCGCGGTAATACGTA<br>GGTGGCAAGCGGTCTCCGGAATTATTGGGCG-<br>TAAAGGGCTCGCAC-<br>CGCCGTTTCTTAAGTCTGATGTGAAAGCCCCCGGCTCAACC<br>GGGGAGGGTCATTGGAAACTGGGGAACTTGAGTGCGAGAA-<br>GAGGAGAGTGGAATTCCCCGTG-<br>TAGCGGTGAAATGCGAAGAGATGTGGAGGAACACCAGTG<br>GCGAAGGGCGACTCCTCTGGTCTGTAACCTGACGCTGAG-<br>GAGACAAAAAACCTGGGG-<br>GAGCCAACAGGATTAGATACCTCTGGTAGTCC
1842480	k__Bacteria, p__Proteobacteria, c__Gammaproteobacteria, o__Chromatiales, f__Ectothiorhodospiraceae, g__, s__	TCCTACGGGAGGCAGCAGTGGGGAA-<br>TATTGGACAATGGGGGCAACCCTGATCCAG-<br>CAATGCCGCGTGTGTGAAGAAGGCCTGCGGGTTGTAAAGCA<br>CTTTCGGTCGGGAAGAGTGCCGGTATGGTTAATAAC-<br>CATGCCGGTTGACGTTACCGGCAGAA-<br>GAAGCACCGGCTAACTCCGTGCCAGCAGCCGCGGTAATAC<br>GGAGGGTGCGAGCGTTAATCGGAATTACTGGGCG-<br>TAAAGGGTGCGTAGGCGGTTGAATCAG-<br>TCGGATGTGAAAGCCCCGGGCTTAACCTGGGAATTGCATTC<br>GATACTGTTCAGCTAGAGTACGGTAGAGGGTGGTG-<br>GAACTCCAGGTGTAGCGGTGAAATGCG-<br>TAGATATCTGGAAGAACACCAGTGGCGAAGGCGGCCACCT<br>GGATCGTAACTGACGCTGAGGCACGAAAGCGTGGGGAG-<br>CAAACAGGATTAGATACCCTGGTAGTCCA
1130640	k__Bacteria, p__Actinobacteria, c__Actinobacteria, o__Actinomycetales, f__Intrasporangiaceae, g__Terracoccus, s__	CCTACGGGAGGCAGCAGTGGGGAATATTGCACAATGGGCG-<br>GAAGCCTGATGCAGCGAC-<br>GCCGCGTGAGGGATGACGGCCTTCGGGTTGTAAACCTCTTT<br>CAGCAGGGAAGAAGCGAAAGTGACGGTACCTGCAGAA-<br>GAAGCACCGGCTAACTACGTGCCAG-<br>CAGCCGCGGTAATACGTAGGGTGCGAGCGTTGTCCGGAATT<br>ATTGGGCGTAAAGAGCTTGTAGGCGGTTT-<br>GTCGCGTCTGCTGTGAAAATCCGGGGCTCAACCCCGGACTT<br>GCAGTGGGTACGGGCAGACTAGAGTGTGGTAGGGGA-<br>GACTGGAATTCCTGGTGTAGCGGTG-<br>GAATGCGCAGATATCAGGAGGAACACCGATGGCGAAGGCA<br>GGTCTCTGGGCCACTACTGACGCTGAGAAGCGAAA-<br>GCATGGGGAGCGAACAGGATTAGATACCCTGGTAGTCC
2251909	k__Bacteria, p__Firmicutes, c__Clostridia, o__Clostridiales, f__Peptostreptococcaceae, g__, s__	CCTACGGGAGGCAGCAGTGGGGAA-<br>TATTGCACAATGGGCGAAAGCCTGATGCAGCAAC-<br>GCCGCGTGAGCGATGAAGGCCTTCGGGTCGTAAAGCTCTGT<br>CCTCAAGGAAGATAATGACGGTACTTGAGGAGGAA-<br>GCCCCGGCTAACTACGTGCCAG-<br>CAGCCGCGGTAATACGTAGGGGGCTAGCGTTATCCCGGAAT<br>TACTGGGCGTAAAGGGTGCGTAGGTGGTTTTTT-

		NAAGTCAGAAGTGAAAGGCTAC-<br>GGCTCAACCGTAGTAAGCTTTTGAAACCTAGAGAACTTGAG<br>TGCAGGAGAGGAGAGTAGAATTCCTAGTG-<br>TAGCGGTGAAATGCGTAGATATTAGGAGGAA-<br>TACCAGTAGCGAAGGCGGCTCTCTGGACTGTAACTGACACT<br>GAGGCACGAAAGCGTGGGGAGCAAACAGGATTAGA-<br>TACCCTGGTAGTC
2331572	k__Bacteria, p__Proteobacteria, c__Gammaproteobacteria, o__Xanthomonadales, f__Xan-thomonadaceae, g__, s__	TCCTACGGGAGGCAGCAGTGGGGAA-<br>TATTGGACAATGGGCGAAAGCCTGATCCAG-<br>CAATGCCGCGTGTGTGAAGAAGGCCCTCGGGTTGTAAAGCA<br>CTTTTATCAGGAACGAAATCTGCACGGTTAATACCCGTG-<br>TAGTCTGACGGTACCTGAGGAA-<br>TAAGCACCGGCTAACTCCGTGCCAGCAGCCGCGGTAATACG<br>GAGGGTGCAAGCGTTAATCGGAATTACTGGGCGTAAA-<br>GCGTGCGCAGGCGGTTAG-<br>TTAAGTCTGCTGTGAAAGCCCCGGGCTCAACCTGGGAATTG<br>CAGTGGATACTGGCTAGCTAGAGTGTGGTAGAGGAAGGTG-<br>GAATTCCCGGTG-<br>TAGCGGTGAAATGCGTAGAGATCGGGAGGAACATCAGTGG<br>CGAAGGCGGCCTTCTGGACCAACACTGACGCTCATGCAC-<br>GAAAGCGTGGGGAGCAAACAG-<br>GATTAGATACCCTGGTAGTCCA
1085410	k__Bacteria, p__Firmicutes, c__Bacilli, o__Lactobacillales, f__Streptococcaceae, g__Strepto-coccus, s__	CCTACGGGAGGCAGCAGTAGGGAATCTTCGGCAATGGAC-<br>GGAAGTCTGACCGAGCAAC-<br>GCCGCGTGAGTGAAGAAGGTTTTCGGATCGTAAAGCTCTGT<br>TGTAAGAGAAGAACGAGTGTGAGAGTGGAAAGTTCACAC-<br>TGTGACGGTATCTTAC-<br>CAGAAAGGGACGGCTAACTACGTGCCAGCAGCCGCGGTAA<br>TACGTAGGTCCCGAGCGTTGTCCGGATTTATTGGGCGTAAA-<br>GCGAGCGCAGGCGGTTAGA-<br>TAAGTCTGAAGTTAAAGGCTGTGGCTTAACCATAGTACGCT<br>TTGGAAACTGTTTAACTTGAGTGCAAGAGGGGAGAGTG-<br>GAATTCCATGTG-<br>TAGCGGTGAAATGCGTAGATATATGGAGGAACACCGGTGG<br>CGAAAGCGGCTCTCTGGCTTGTAACTGAC-<br>GCTGAGGCTCGAAAGCGTGGGGAGCAAACAG-<br>GATTAGATACCCTGGTAGTC
928538	k__Bacteria, p__Firmicutes, c__Bacilli, o__Bacillales, f__Staphylococcaceae, g__Staph-ylococcus, s__	CCTACGGGAGGCAGCAGTAGGGAATCTTCCGCAATGGAC-<br>GCAAGTCTGACGGAGCAAC-<br>GCCGCGTGAGTGATGAAGGTCTTCGGATCGTAAAACTCTGT<br>TATTAGGGAAGAACAAATGTGTAAGTAACTATGCACATCTT-<br>GACGGTACCTAAACAGAAA-<br>GCCACGGCTAACTACGTGCCAGCAGCCGCGGTAATACGTAG<br>GTGGCAAGCGTTATCCGGAATTATTGGGCGTAAA-<br>GCGCGCG-<br>TAGGCGGTTTTTTAAGTCTGATGTGAAAGCCCACGGCTCAA<br>CCGTGGAGGGTCATTGGAAACTGGAAAACTTGAGTG-<br>CAGAAGAGGAAAGTG-<br>GAATTCCATGTGTAGCGGTGAAATGCGCAGAGATATGGAGG

		AACACCAGTGGCGAAGGCGACTTTCTGGTCTGTAACTGAC-GCTGATGTGCGAAAGCGTGGG-GATCAAACAGGATTAGATACCCTGGTAGTC
652444	k__Bacteria, p__Actinobacteria, c__Actinobacteria, o__Actinomycetales, f__Streptomycetaceae, g__, s__	CCTACGGGAGGCAGCAGTGGGGAATCTTGCG-CAATGGGCGAAAGCCTGACGCAGCAAC-GCCGCGTGGGGGATGAAGGCCTTCGGGTTGTAAACCTCTTTCACCATCGACGAAGCCCTTTGGGGTGACGGTAGGTGGG-GAAGAAGCACCGGCTAACTAC-GTGCCAGCAGCCGCGGTAATACGTAGGGTGCGAGCGTTGTCCGGATTTATTGGGCGTAAAGAGCTCGTAGGCGGTTT-GTCGCGTCGGCCGTGAAAAC-CTACAGCTTAACTGTGGGCTTGCGGTCGATACGGGCAGACTTGAGTTCGGCAGGGGAGACTGGAATTCCTGGTG-TAGCGGTGAAATGCGCAGATATCAGGAG-GAACACCGGTGGCGAAGGCGGGTCTCTGGGCCGATACTGACGCTGAGGAGCGAAAGCGTGGGGAGCGAACAGGATTAGA-TACCCTGGTAGTCC
2770490	k__Bacteria, p__Proteobacteria, c__Alphaproteobacteria, o__Caulobacterales, f__Caulobacteraceae, g__, s__	CCTACGGGAGGCAGCAGTGGGGAATTTTCCG-CAATGGGCGAAAGCCTGAC-GCAGCCATGCCGCGTGAATGATGAAGGTCTTAGGATTGTAAAATTCTTTCACCGGGGACGATAATGACGGTACCCGGAGAA-GAA-GCCCCGGCTAACTTCGTGCCAGCAGCCGCGGTAATACGAAGGGGGCTAGCGTTGCTCGGAATTACTGGGCG-TAAAGGGAGCGTAGGCGGACTGTTTAG-TCAGAGGTGAAAGCCCAGGGCTCAACCTTGGAATTGCCTTTGATACTGGCAGTCTTGAGTACGGAAGAGGTATGTG-GAACTCCGAGTGTAGAGGTGAAATTCG-TAGATATTCGGAAGAACACCAGTGGCGAAGGCGACATACTGGTCCGTTACTGACGCTGAGGCTCGAAAGCGTGGGGAG-CAAACAGGATTAGATACCCTGGTAGTC
606349	k__Bacteria, p__Actinobacteria, c__Actinobacteria, o__Actinomycetales, f__Pseudonocardiaceae, g__Amycolatopsis, s__	CCTACGGGAGGCAGCAGTGGGGAATATTGCACAATGGGCG-CAAGCCTGATGCAGCGAC-GCCGCGTGAGGGATGACGGCCTTCGGGTTGTAAACCTCTTTCGCCAGGGACGAAGCGCAAGTGACGGTACCTGGATAA-GAAGCACCGGCTAACTACGTGCCAG-CAGCCGCGGTAATACGTAGGGTGCGAGCGTTGTCCGGAATTATTGGGCGTAAAGAGCTCGTAGGCGGTTT-GTCGCGTCGTTCGTGAAAACTCCACGCTTAAC-GTGGAGCGTGCGGGCGATACGGGCAGACTTGAGTTCGGTAGGGGAGACTGGAATTCCTGGTGTAGCGGTGAAATGCGCAGA-TATCAGGAGGAACAC-CGGTGGCGAAGGCGGGTCTCTGGGCCGATACTGACGCTGAGGAGCGAAAGCGTGGGGAGCGAACAGGATTAGA-TACCCTGGTAGTCC
702094	k__Bacteria, p__Actinobacteria, c__Actinobacteria, o__Actinomycetales, f__Pseudonocardiaceae, g__, s__	CCTACGGGAGGCAGCAGTGGGGAA-TATTGCACAATGGGCGAAAGCCTGATGCAGCAAC-GCCGCGTGAGGGATGACGGCCTTCGGGTTGTAAACCTCTTTCGGCGCCGACGAAGCTTTCGGGTGACTGTAGGCGCAGAA-GAAGCACCGGCCAACTAC-GTGCCAGCAGCCGCGGTAATACGTAGGGTGCGAGCGTTGTC

		CGGAATTATTGGGCGTAAAGGGCTCG-<br>TAGGCGGCCTGTCGCGTCCGTCGTGAAA-<br>GCCCGGGGCTCAACCCCGGGTCTGCGGTGGATACGGGCAG<br>GCTAGAGTCCGGCAGGGGATGACTGGAATTCCTGGTG-<br>TAGCGGTGAAATGCGCAGATATCAG-<br>GAGGAACACCAGTGGCGAAGGCGGGTCTCTGGGCCGGAAC<br>TGACGCTGAGGAGCGAAAGCGTGGGGAGCGAACAG-<br>GATTAGATACCCTGGTAGTC
830322	k__Bacteria, p__Acidobacteria, c__Acidobacteriia, o__Acidobacteriales, f__Acidobacteriaceae, g__, s__	CCTACGGGAGGCAGCAGTGGGGAATTTTGCGCAATGGGG-<br>GAAACCCTGACGCAGCAAC-<br>GCCGCGTGGAGGATGAAGTCCCTTGGGACGTAAACTCCTTT<br>CGATCGGGACGATTATGACGGTACCGGAAGAAGAA-<br>GCCCCGGCTAACTTCGTGCCAG-<br>CAGCCGCGGTAATACGAGGGGGGCAAGCGTTGTTCGGAATT<br>ATTGGGCGTAAAGGGAGTGTAGGCGGTTTGG-<br>TAAGTCTCGTGTGAAATCTTCAGGCTCAAC-<br>CTGAAGTCTGCACGAGAAACTGCCGGGCTTGAGTATGGGAG<br>AGGTGAGTGGAATTCCTGGTGTAGCGGTGAAATGCGTAGA-<br>TATCAGGAGGAACAC-<br>CTGTGGCGAAAGCGGCTCACTGGACCATAACTGACGCTGAG<br>ACTCGAAAGCTAGGGGAGCAAACAGGATTAGATACCCTGG-<br>TAGTCC
1131894	k__Bacteria, p__Cyanobacteria, c__Chloroplast, o__Streptophyta, f__, g__, s__	CCTACGGGAGGCAGCAGTGGGGAATTTTCCG-<br>CAATGGGCGAAAGCCTGACGGAG-<br>CAATGCCGCGTGGAGGTAGAAGGCCGTCGTGAACTTCTTTT<br>CCCGGAGAAGAAGCAATGACGGTATCCGGGGAATAA-<br>GCATCGGCTAACTCTGTGCCAG-<br>CAGCCGCGGTAAGACAGAGGATGCAAGCGTTATCCGGAAT<br>GATTGGGCGTAAAGCGTCTG-<br>TAGGTGGCTTTTTAAGTCCGCCGTCAAATCCCAGGGCTCAA<br>CCCTGGACAGGCGGTGGAAACTACCAAGCTGGAGTACGG-<br>TAGGGG-<br>CAGAGGGAATTTCCGGTGGAGCGGTGAAATGCGTAGAGAT<br>CGGAAAGAACACCAACGGCGAAAGCAC-<br>TCTGCTGGGCCGACACTGACACTGAGAGACGAAA-<br>GCTAGGGGAGCGAATGGGATTAGATACCCCAGTAGTC
823036	k__Bacteria, p__Firmicutes, c__Bacilli, o__Bacillales, f__Bacillaceae, g__Bacillus, s__	CCTACGGGAGGCAGCAGTAGGGAATCTTCCGCAATGGAC-<br>GAAAGTCTGACGGAGCAAC-<br>GCCGCGTGAGTGATGAAGGTTTTCGGATCGTAAAGCTCTGTT<br>GTTAGGGAAGAACAAGTGCGAGAGTAACTGCTCGCACCTT-<br>GACGGTACCTAACCAGAAA-<br>GCCACGGCTAACTACGTGCCAGCAGCCGCGGTAATACGTAG<br>GTGGCAAGCGTTGTCCGGAATTATTGGGCG-<br>TAAAGGGCTCG-<br>CAGGCGGTTTCTTAAGTCTGATGTGAAAGCCCCCGGCTCAA<br>CCGGGGAGGGTCATTGGAAACTGGGAAACTTGAGTG-<br>CAGAAGAGGAGAGTGGAATTCCAC-<br>GTGTAGCGGTGAAATGCGTAGAGATGTGGAGGAACACCAG<br>TGGCGAAGGCGACTCTCTGGTCTGTAACTGACGCTGAG-<br>GAGCGAAAGCGTGGGGAGCGAC-<br>CAGGATTAGATACCCTGGTAGTCC

663573	k__Bacteria, p__Proteobacteria, c__Gammaproteobacteria, o__Xanthomonadales, f__Xanthomonadaceae, g__Stenotrophomonas, s__	CCTACGGGAGGCAGCAGTGGGGAATATTGGACAATGGGCG-<br>CAA-<br>GCCTGATCCAGCCATACCGCGTGGGTGAAGAAGGCCTTCGG<br>GTTGTAAAGCCCTTTTGTTGGGAAA-<br>GAAATCCAGCCGGCTAATACCTGGTTGGGATGACGG-<br>TACCCAAAGAATAAGCACCGGCTAACTTCGTGCCAGCAGCC<br>GCGGTAATACGAAGGGTGCAAGCGTTACTCG-<br>GAATTACTGGGCGTAAAGCGTGCG-<br>TAGGTGGTCGTTTAAGTCCGTTGTGAAAGCCCTGGGCTCAA<br>CCTGGGAACTGCAGTGGATACTGGGCGACTAGAGTGTGG-<br>TAGAGGGTAGCG-<br>GAATTCCTGGTGTAGCAGTGAAATGCGTAGAGATCAGGAGG<br>AACATCCATGGGCGAAGCAGCTACCTGGACCAACAC-<br>TGACACTGAGGCACGAA-<br>GCAGCGTGGGGAGCAACAGATTATATACCCTGGTATTCC
3377451	k__Bacteria, p__Actinobacteria, c__Actinobacteria, o__Actinomycetales, f__Rarobacteraceae, g__, s__	CCTACGGGAGGCAGCAGTGGGGAA-<br>TATTCCACAATGGGCGAAA-<br>GCCTGATGCAGCGACCCCGCGTGAGGGATGACCGCCTTCNG<br>GGTTATAAAACTCTTTCCAGTAAGGAAGAA-<br>GCGAAAAGTGACGGTACTTGCAAAAA-<br>NAAGCGCCGGCTAACTACGTGCCAGCAGCCGCGGTAATAC<br>GTAGGGCGCAAGCGTTGTCCGGAATTATTNGGGCGTAAA-<br>GAGCTCGTAGGTGGTTT-<br>GTCGCGTCTGGTGTGAAATTCCCAAGGCTCAACCTTTGGGCT<br>TGCATTGGGTACGGGCAGACTAGAGTGCGGTAGGGGA-<br>GACTGGAATTCCTGGTGTAGCGGTG-<br>GAATGCGCAGATATCAGGAGGAACACCGATGGCGAAGGCA<br>GGTCTCTGGGCCGCAACTGACGCTGAGGAGCGAAA-<br>GCATGGGGAGCGAACAGGATTAGATACCCTGGTAGTC
3393186	k__Bacteria, p__Proteobacteria, c__Betaproteobacteria, o__Neisseriales, f__Neisseriaceae, g__, s__	CCTACGGGAGGCAGCAGTGGGGAATTTTGGACAATGGGGG-<br>GAACCCTGATCCAGCCATGCCGCGTGTATGAAGAAGGCCTT<br>AGGGTTGTAAAGTACTTTTGTTAGGGAAGAAAAGCTAG-<br>TTTTGAATAAAGATTAGTGCTGAC-<br>GGTACCTAAAGAATAAGCACCGGCTAACTACGTGCCAGCA<br>GCCGCGGTAATACGTAGGGTGCGAGCGTTAATCG-<br>GAATTATTGGGCGTAAAGCGAGTG-<br>CAGACGGTTGTTTAAGCCAGATGTGAAATCCCCGAGCTTAG<br>CTTGGGACGTGCATTTGGAACTGGATAACTAGAG-<br>TGTGTCAGAGGGAGGTAGAATTCCACATGTAGCGGTGGAAT<br>GCGTAGAGATGTGGAGGAATAC-<br>CGATGGCGAAGGCAGCCTCCTGGGATAACACTGACGTT-<br>GAGGCTCGAAAGCGTGGGGAGCAAACAGGATTAGATACCC<br>TGGTAGTC
637528	k__Bacteria, p__Actinobacteria, c__Actinobacteria, o__Actinomycetales, f__Dermatophilaceae, g__Piscicoccus, s__intestinalis	CCTACGGGAGGCAGCAGTGGGGAATATTGCACAATGGGCG-<br>CAAGCCTGATGCAGCGAC-<br>GCCGCGTGAGGGATGAAGGCCTTCGGGTTGTAAACCTCTTT<br>CGTCAGGGAAGAAGCGAAAGTGACGGTACCTGAAGAA-<br>GAAGCACCGGCTAACTACGTGCCAG-<br>CAGCCGCGGTAATACGTAGGGTGCGAGCGTTGTCCGGAATT<br>ATTGGGCGTAAAGAGCTTGTAGGCGGTTT-

		GTCGCGTCTGCCGTGAAAAC-<br>CTAGGGCTTAACTCTGGGCGTGCGGTGGGTACGGGCAGGCT<br>AGAGTGTGGTAGGGGAGACTGGAATTCCTGGTG-<br>TAGCGGTGAAATGCGCAGATATCAGGAG-<br>GAACACCGATGGCGAAGGCAGGTCTCTGGGCCATAACTGA<br>CGCTGAGAAGCGAAAGCATGGGGAGCGAACAGGATTAGA-<br>TACCCTGGTAGTCC
3466729	k__Bacteria, p__Firmicutes, c__Clostridia, o__Clostridiales, f__[Tissierellaceae], g__, s__	CCTACGGGAGGCGGCAGTGGGGAATATTGCACAATGGAG-<br>GAAACTCTGATGCAGCGAC-<br>GCCGCGTGAGCGATGAAGGTCTTCGGATCGTAAAGCTCTGT<br>CCTAAGGGACGATAATGACGGTACCTTAGGAGGAA-<br>GCTCCGGCTAACTACGTGCCAG-<br>CAGCCGCGGTAATACGTAGGGAGCAAGCGTTGTCCGGATTT<br>ATTGGGCGTAAAGGGTGCGTAGGCGGCCTGA-<br>TAAGTCAGATGTGAAAGGCGGAGGCTCAAC-<br>CTCCGTTAGCATCTGAAACTGTCAGGCTTGAGTGAAGGAGA<br>GGAAAGTGGAATTCCTAGTGTAGCGGTGAAATGCGTAGA-<br>TATTAGGAGGAATAC-<br>CGGTGGCGAAGGCGACTTTCTGGACTTAAACTGACGCTGAG<br>GCACGAAAGTGTGGGGAGCAAACAGGATTAGATACCCTGG-<br>TAGTC
3587177	k__Bacteria, p__Planctomycetes, c__Phycisphaerae, o__Phycis-phaerales, f__Phycisphaeraceae, g__, s__	CCTACGGGAGGCTGCAGTAACGAATATTCCG-<br>CAATGCGCGAAAGCGTGACGGAG-<br>CAATGCCGCGTGCAGGATGAAGCCCCTCGGGGTGTAAACTG<br>CTGTCAGGGTCCACCAAGCTCTGAGGAGACCCAGAGGAA-<br>GAGCCGGCTAACTCTGTGCCAG-<br>CAGCCGCGGTAATACAGAGGGCTCGAGCGTTAATCGGAAT<br>CACTGGGCTTAAAGGGTGCGCAGGCGGATGGG-<br>CAAGTGCTTTGTGAAATCCCACGGCTTAAC-<br>CGTGGAATGGCTTGGCAGACTGCCCGTCTTGAGCCCGGCAG<br>GGGCCGTCGGAACGGTAGGTGGAGCGGTGAAATGCGTAGA-<br>TATCTACCGGAAC-<br>GCCAAAGGTGAAGACAGACGGCTGGGCCGGCGCTGACGCT<br>CAGGCACGAAAGCGTGGGGAGCGAACGGGATTAGA-<br>CACCCCGGTAGTCC
1065051	k__Bacteria, p__Actinobacteria, c__Thermoleophilia, o__Soliru-brobacterales, f__, g__, s__	CCTACGGGAGGCAGCAGTGGGGAATCTT-<br>GCACAATGCGCGAAAGCGTGATGCAGCAAC-<br>GCCGCGTGAGGGAAGACGGCCTTCGGGTTGTAAACCTCTTT<br>CAGTTGGGACGAAGGTCGGGTGGTTAATAGC-<br>TATTCCGACTGACGGTACCTTCACAAGAA-<br>GCCCCGGCTAACTACGTGCCAGCAGCCGCGGTAATACGTAG<br>GGGGCAAGCGTTGTCCGGAATCATTGGGCGTAAA-<br>GAGCGTGTAGGCGGCTTGA-<br>TAAGTCGGCTGTGAAAGTCAGGGGCTCAACCCTTGAATGCC<br>GGCCGATACTGTCAAGCTAGAGTCCGGAAGAGGCGAGTG-<br>GAATTCCTGGTG-<br>TAGCGGTGAAATGCGCAGATATCAGGAGGAACACCAATGG<br>CGAAGGCAGCTCGCTGGGACGGTACTGACGCTGAGAC-<br>GCGAAAGCGTGGGGAGCAAACAG-<br>GATTAGATACCCTGGTAGTCC

805492	k__Bacteria, p__TM6, c__SJA-4, o__, f__, g__, s__	CCTACGGGAGGCAGCAGTGAGGAATATTGCG-<br>CAATGGGCGAAAGCCTGACGCAGCGAC-<br>GCCGCGTGGAGGATGAAGGTCTTTGGATTGTAAACTTCTGTT<br>AAGTGGGAAGAAAGACGGGATTTTAATACGATCCCGG-<br>GATGACGGTACCATTAGAGAAA-<br>GCACCGGCTAACTTCGTGCCAGCAGCCGCGGTAATACGAAG<br>GGTGCTAGCGTTATTCGGAATTATTGGGCGTAAAGGGTGCG-<br>CAGGCGG-<br>TATGTTAAGTCAACTGTTAAATACCTCAGCTTAACTGGGGAT<br>CAGCGGTAGATACTGGCATACTAGAGAATGGAAGAGA-<br>GAAGTGGAATTCTTGGAGTAGCGG-<br>TAAAATGCGTAGATCTCAAGAGGAACACCGATGGCGAAGG<br>CAGCTTCTTGGTCCATTCTGACGCTCATGCACGAAA-<br>GCGTGGGGAGCAAACAGGATTAGATACCCTGGTAGTCC
3724491	k__Bacteria, p__Firmicutes, c__Clostridia, o__Clostridiales, f__[Tissierellaceae], g__Sporan-aerobacter, s__	CCTACGGGAGGCAGCAGTGGGGAATATTGCACAATGGAG-<br>GAAACTCTGATGCAGCGAC-<br>GCCGCGTGAGCGATGAAGGTTTTCGAATCGTAAAGCTCTGT<br>CCTAAGAGACGATAATGACGGTACCTTAGGAGGAA-<br>GCCCCGGCTAACTACGTGCCAG-<br>CAGCCGCGGTAATACGTAGGGGGCGAGCGTTGTCCGGATTT<br>ATTGGGCGTAAAGGGTGCGTAGGCGGCCTT-<br>GTAAGTCAGATGTGAAATCTCACGGCTTAAC-<br>CGTGGTAAGCATTTGAAACTGTGAGGCTTGAGTACAGGAGA<br>GGAGAGTGGAATTCCTAGTGTAGCGGTGAAATGCGTAGA-<br>TATTAGGAGGAATACCAG-<br>TGGCGAAGGCGACTCTCTGGACTGTAACTGACGCTGAGGCA<br>CGAAAGCGTGGGGAGCAAACAGGATTAGATACCCTGG-<br>TAGTC
1104935	k__Bacteria, p__Planctomycetes, c__Phycisphaerae, o__Phycis-phaerales, f__Phycisphaeraceae, g__, s__	CCTACGGGAGGCTGCAGCAACGAATCTTCCG-<br>CAATGGGCGAAAGCCTGACGGAG-<br>CAATGCCGCGTGAAGGATGAAGCCCCTCGGGGTGTAAACTT<br>CTGTCAGGGTCTAGAAACGTTGATCGGACCCAGAG-<br>GAAGGGCCGGCTAACTTCGTGCCAG-<br>CAGCCGCGGTAATACGAAGGGCCCAAGCGTTGCGCGGAAT<br>TACTGGGCTTAAAGGGTGCGCAGGCGGACCTG-<br>CAAGTACCCTGTGAAATCCCACAGCTTAACTGTGGAATTGC<br>AGGGTATACTGCAGGTCTTGAGCCCGGCAGAGGCCATCG-<br>GAACTCTAGGTG-<br>GAGCGGTGAAATGCGTAGATATCTAGAGGAACGCCAAAGG<br>TGAAGACAGATGGCTGGGCCGGTGCTGACGCTCAGGCAC-<br>GAAAGCGTGGGGAGCGAACGG-<br>GATTAGATACCCCGGTAGTCC
3843950	k__Bacteria, p__Actinobacteria, c__Actinobacteria, o__Actinomy-cetales, f__Streptomycetaceae, g__Streptomyces, s__	CCTACGGGAGGCAGCAGTGGGGAA-<br>TATTGCACAATGGGCGAAAGCCTGATGCAGCGAC-<br>GCCGCGTGAGGGATGACGGCCTTCGGGTTGTAAACCTCTTT<br>CAGCAGGGAAGAAGCGCAAGTGACGGTACCTGCAGAA-<br>GAAGCGCCGGCTAACTACGTGCCAG-<br>CAGCCGCGGTAATACGTAGGGCGCAAGCGTTGTCCGGAATT<br>ATTGGGCGTAAAGAGCTCGTAGGCGGCTTGTCACGTCGGTT-<br>GTGAAA-<br>GCCCGGGGCTTAACCCCGGGTCTGCAGTCGATACGGGCAGG

		CTAGAGTGTGGTAGGGGAGATCGGAATTCCTGGTG-TAGCGGTGAAATGCGCAGATATCAG-GAGGAACACCGGTGGCGAAAGGCGGATCTCTGGGCCATTACTGACGCTGAGGAGCGAAAGCGTGGGGAGCGAACAG-GATTAGATACCCTGGTAGTCCA
4001495	k__Bacteria, p__Cyanobacteria, c__Chloroplast, o__Streptophyta, f__, g__, s__	CCTACGGGAGGCAGCAGTGGGGAATTTTCCG-CAATGGGCGAAAGCCTGACGGAG-CAATGCCGCGTGAAGGCAGAAGGCCCACGGGTCATGAACTTCTTTTCTCGGAGAAGAAAAATGACGGTATCTGAGGAA-TAA-GCATCGGCTAACTCTGTGCCAGCAGCCGCGGTAAGACAGAGGATGCAAGCGTTATCCGGAATGATTGGGCGTAAA-GCGTCTG-TAGGTGGCTTTTCAAGTCCGCCGTCAAATTCCAGGGCTCAACCCTGGACAGGCGGTAGAAACTACCAAGCTGGAGTACGG-TAGGGG-CAGAGGGAATTTCCGGTGGAGCGGTGAAATGCGTTGAGATCGGAAAGAACACCAACGGCGAAAGCAC-TCTGCTGGGCCGACACTGACACTGAGAGACGAAA-GCTAGGGGAGCAAATGGGATTAGATNCCCCAGTAGTC
4093084	k__Bacteria, p__Actinobacteria, c__Acidimicrobiia, o__Acidimicrobiales, f__, g__, s__	CCTACGGGAGGCAGCAGTGGGGAATCTTGCG-CAATGGGCGAAAGCCTGACGCAGCAAC-GCCGCGTGCGGGACGAAGGCCTTCGGGTTGTAAACCGCTTTCAGCAGGGACGAAACTGACGGTACCTGCAGAAGAA-GCCCCGGCCAACTACGTGCCAG-CAGCCGCGGTAATACGTAGGGGGCGAGCGTTGTCCGGATTTATTGGGCGTAAAGAGCTCGTAGGCGGCTGGG-CAAGTCGGGTGTGAAAACTCCAGGCTCAACTTGGAGACGCCACTTAATACTGCCCTGGCTTGAGTCCGGTAGGGGAGCGCG-GAATTCCTGGTG-TAGCGGTGAAATGCGCAGAGATCAGGAGGAACACCGGTGGCGAAGGCGGCGCTCTGGGCCGGTACTGACGCTGAG-GAGCGAAAGCGTGGGGAGCGAACAG-GATTAGATACCCTGGTAGTC
4303163	k__Bacteria, p__Actinobacteria, c__Actinobacteria, o__Actinomycetales, f__Pseudonocardiaceae, g__Pseudonocardia, s__	CTCTTACGGGAGGCAGCAGTGGGGAATTTTGCG-CAATGGGCGGAAGCCTGACGCAGCGAC-GCCGCGTGGGGGATGACGGCCTTCGGGTTGTAAACCTCTTTCGCCATCGACGAAGCCTTGTGTGGTGACGATAGGTGGA-GAAGAAGCACCGGCCAACTAC-GTGCCAGCAGCCGCGGTAATACGTAGGGTGCGAGCGTTGTCCGGAATTATTGGGCGTAAAGAGCTCG-TAGGCGGTGTGTCGCGTCGATCGTGAAAACTT-GGGGCTCAACTCTGAGCTTGCGGTCGATACGGGCATCACTGGAGTTCGGCAGGGGAGACTGGAATTCCTGGTG-TAGCGGTGAAATGCGCAGATATCAGGAG-GAACACCGGTGGCGAAGGCGGGTCTCTGGGCCGATACTGACGCTGAGGAGCGAAAGCGTGGGGAGCGAACAGGATTAGA-TACCCTGGTAGTC

4314389	k__Bacteria, p__Actinobacteria, c__Actinobacteria, o__Actinomycetales, f__Pseudonocardiaceae, g__, s__	CCTACGGGAGGCAGCAGTGGGGAATCTTGCG-CAATGGGCGAAAGCCTGACGCAGCGAC-GCCGCGTGAGGGATGACGGCCTTCGGGTTGTAAACCTCTTTCAGCTCCGACGAAGCGAGAGTGACGGTAGGAGCAGAA-GAAGCACCGGCCAACTACGTGCCAG-CAGCCGCGGTAATACGTAGGGTGCAAGCGTTGTCCGGAATTATTGGGCGTAAAGAGCTCG-TAGGCGGTCTGTCGCGTCGGCTGTGAAAACTCGGGGCTCAACTCCGAGCTTGCAGTCGATACGGGCAGGCTAGAGTGTCGG-CAGGGGAGACTGG-GAATTCCTGGTGTAAGCGGTGAAATGCGGCAGATATCAGGAGGAACACCGGGTGGCGAAAGGCGGGTCTCTGGGCCGA-TACTGACACTGAGGAGCGAAAA-GCGTGGGGAGCAAAACAAGAATAGATACCCCTGTTAGTC
4323733	k__Bacteria, p__Actinobacteria, c__Actinobacteria, o__Actinomycetales, f__Mycobacteriaceae, g__Mycobacterium, s__	CCTACCGGGGAGGGCAGGCAGTTTGGG-GAATTATTGCCACACATTGGGGCGCCAAGCCTT-GAATGCAGGCGACGCCGCGTGAAGGGATGACGGCCTTTCGGGTTGTTAAACCTTCTTTCAGCACAGACGAAGCGCGAG-TGACGGTATGTG-CAGAAGGAAGGACCGGCCCAACTACGTGCCAGCAGCCCGCCGGTATTACGTAGGGTCCGAGCGTTGTCCG-GAATTACTGGGCGTAAAGAGCTCG-TAGGTGGTTTGTCGCGTTGTTCGTGAAAACTCACAGCTTAACTGTGGGCGTGCGGGCGATACGGGCAGACTGGAGTACTG-CAGGGGAGACTGGAATTCCTGGTG-TAGCGGTGGAATGCGCAGATATCAGGAGGAACACCGGTGGCGAAGGCGGGTCTCTGGGCAGTAACTGACGCTGAG-GAGCGAAAGCGTGGGGAGCGAACAG-GATTAGATACCCTGGTAGTCC
4326463	k__Bacteria, p__Planctomycetes, c__Phycisphaerae, o__Phycisphaerales, f__, g__, s__	CCTACGGGAGGCTGCAGTAACGAATATTGGACAATGCGG-GAAACCGTGATCCAG-CAATGCCGCGTGCGGGACGAAGCCCCTCGGGGTGTAAACCGCTGTCAGGGCCTACCAACTCAATGAGGAGGCCCAGAG-GAAGAGAC-GGCTAACTCTGTGCCAGCAGCCGCGGTAATACAGAGGTCTCGAGCGTTAATCGGAATCACTGGGCTTAAAGGGTGCG-CAGGCGGGCCCGCGAGTGCTTT-GTGAAATCCCACGGCTCAACCGTGGAGCAGCTTGGCATACTGCGGGTCTTGAGCTCGGCAGGGGTCGTCGGAAC-GCTAGGTGGAGCGGTGAAATGCGTAGA-TATCTAGCGGAACGCCAAAGGTGAAGACAGACGACTGGGCCGACGCTGACGCTCAGGCACGAAAGCGTGGGGAGCAAAC-GGGATTAGATACCCCGGTAGTCC
656881	k__Bacteria, p__Proteobacteria, c__Gammaproteobacteria, o__Enterobacteriales, f__Enterobacteriaceae, g__Escherichia, s__coli	CCTACGGGAGGCAGCAGTGGGGAATATTGCACAATGGGCG-CAA-GCCTGATGCAGCCATGCCGCGTGTATGAAGAAGGCCTTCGGGTTGTAAAGTACTTTCAGCGGGGAGGAAGGGAG-TAAAGTTAATACCTTTGCTCATTGAC-GTTACCCGCAGAAGAAGCACCGGCTAACTCCGTGCCAGCAGCCGCGGTAATACGGAGGGTGCAAGCGTTAATCG-

		GAATTACTGGGCGTAAAGCGCAC-<br>GCAGGCGGTTTGTTAAGTCAGATGTGAAATCCCCGGGCTCA<br>ACCTGGGAACTGCATCTGATACTGGCAAGCTTGAGTCTCG-<br>TAGAGGGGGG-<br>TAGAATTCCAGGTGTAGCGGTGAAATGCGTAGAGATCTGGA<br>GGAATACCGGTGGCGAAGGCGGCCCCCTGGACGAA-<br>GACTGACGCTCAGGTGCGAAA-<br>GCGTGGGGAGCAAACAGGATTAGATACCCTGGTAGTCC
4413327	k__Bacteria, p__Firmicutes, c__Bacilli, o__Lactobacillales, f__Lactobacillaceae, g__, s__	CCTACGGGAGGCAGCAGTAGGGAATCTTCCACAATGGAC-<br>GAAAGTCTGATGGAGCAAC-<br>GCCGCGTGAGTGAAGAAGGGTTTCGGCTCGTAAAACTCTGT<br>TGTTAAAGAAGAACATATCTGAGAG-<br>TAACTGTTCAGGTATTGACGGTATTTAACCAGAAA-<br>GCCACGGCTAACTACGTGCCAGCAGCCGCGGTAATACGTAG<br>GTGGCAAGCGTTGTCCGGATTTATTGGGCGTAAA-<br>GCGAGCG-<br>CAGGCGGTTTTTTAAGTCTGATGTGAAAGCCTTCGGCTCAAC<br>CGAAGAAGTGCATCGGAAACTGGGAAACTTGAGTG-<br>CAGAAGAGGACAGTGGAACTCCATGTG-<br>TAGTGGTGAAATGCGTAGATATATGGAAGAACACCAGTGG<br>CGAAAGCACCTATTTGGACATGTACTGAC-<br>GCTAAGGCTCGAAAGTGTGGGGAGCAAAAC-<br>GGATTAGATACCCGTGTAGTC
4414230	k__Bacteria, p__Actinobacteria, c__Actinobacteria, o__Actinomycetales, f__, g__, s__	CCTACGGGAGGCAGCAGTGGGGAATATTGCG-<br>CAATGGGCGAAAGCCTGACGCAGCGAC-<br>GCCGCGTGAGGGACGAAGGTCTTCGGATTGTAAACCTCTTT<br>CAGCAGGGACGAAGCGAAAGTGACTGTACCTGCAGAA-<br>GAAGCACCGGCTAACTACGTGCCAG-<br>CAGCCGCGGTAATACGTAGGGTGCAAGCGTTGTCCGGATTT<br>ATTGGGCGTAAAGAGCTCGTAGGCGGTCTGTCGCGTCG-<br>GATGTGAAAACCCAATGGCTTAAC-<br>CTTGGGCCTGCATTCGATACGGGCAGACTAGAGTGTGGTAG<br>GGGAAACTGGAATTCCTGGTGTAGCGGTGGAATGCGCAGA-<br>TATCAGGAGGAACAC-<br>CGATGGCGAAGGCAGGTTTCTGGGCCATAACTGACGCTGAG<br>GAGCGAAAGCGTGGGGAGCAAACAGGATTAGA-<br>TACCCTGGTAGTCC
4461579	k__Bacteria, p__Acidobacteria, c__Acidobacteria-6, o__iii1-15, f__, g__, s__	CCTACGGGAGGCAGCAGTGGGGAATTGTTCGCAATGGGCG-<br>CAAGCCTGACGACGCAAC-<br>GCCGCGTGGAGGACGAAGATTTTCGGATCGTAAACTCCTTT<br>CGATCGGGACGAATCGGTCGCGACCTAACACGTCGCGG-<br>CACTGACGGTACCGAGAGAAGAA-<br>GCCCCGGCTAACTCCGTGCCAGCAGCCGCGGTAATACGGGG<br>GGGGCAAGCGTTGTTCGGAATTACTGGGCG-<br>TAAAGGGCGCGTAGGCGGCCTGCTAAGTCG-<br>GACGTGAAATCCCCCGGCTCAACCGGGGAACTGCGTCCGAT<br>ACTGGTGGGCTCGAATCCGGGAGAGGGATGTG-<br>GAATTCCAGGTGTAGCGGTGAAATGCG-<br>TAGATATCTGGAGGAACACCACCGGTGGCGAAGGGAGCAA<br>TACTGGACCGGATATGACGCTGGGGAGCGAAAGCCAGGG-<br>GAGCAAACGGGATTAGATACCCCAGTAGTC

M04428:485:000000000-J6G24:1:2106:16995:3650 1:N:0:TAAGGCGA+GAGCCTTA/M04428:485:000000000-J6G24:1:2106:16995:3650 2:N:0:TAAGGCGA+GAGCCTTA	k__Bacteria, p__Proteobacteria, c__Gammaproteobacteria, o__Salinisphaerales, f__, g__, s__	CCTACGGGGGGCAGCAGTGGGGAA-TATTGGACAATGGGCGAAA-GCCTGATCCAGCCATGCCGCGTGTGTGAAGAAGGCCCTAGGGTTGTAAAGCACTTTAAGCAGGAAAGAAAA-GCGCCGGGTTAATACCCCGAGTGTGTTGACGG-TACCTGCAGAATAAGCACCGGCTAACTTCGTGCCAGCAGCCGCGGTAATACGAAGGGTGCAAGCGTTAATCG-GAATTACTGGGCGTAAAGCGAGTG-CAGGCGGTTGCGTGTGTCGGATGTGAAAGCCCTGGGCTCAACCTGGGAATTGCATTCGAAACTGCGCGACTAGAATATGG-CAGAGGAAGACGGAATTCCAC-GTGTAGCGGTGAAATGCGTAGATATGTGGAGGAACACCAATGGCGAAGGCAGTCTTCTGGGCCAATATTGAC-GCTGAGGCTCGAAAGCGTGGGTAG-CAAACAGGATTAGATACCCCAGTAGTC
M04428:485:000000000-J6G24:1:1106:13652:5335 1:N:0:TAAGGCGA+GAGCCTTA/M04428:485:000000000-J6G24:1:1106:13652:5335 2:N:0:TAAGGCGA+GAGCCTTA	k__Bacteria, p__Bacteroidetes, c__[Rhodothermi], o__[Rhodothermales], f__[Balneolaceae], g__, s__	CCTACGGGGGGCTGCAGTGAGGAATCTTGCGCAATGGGG-GAAACCCTGAC-GCAGCCATGCCGCGTGCCGGAAGACGGCCCTACGGGTTGTAAACGGCTTTTGACGGGAAGAAACTCTTGAATTCGTTCGA-GATTGACGGTACCGTCCGAA-TAAGCACCGGCTAACTCCGTGCCAGCAGCCGCGGTAATACGGAGGGTGCAAGCGTTGTCCGGAATTATTGGGTG-TAAAGGGTGCGTAGGCGTCCTT-GCAAGTTTTCGGTGAAAGCCACGAGCTCAACTCGGGAAGTGCCGAAAAAACTGCAAGGATTGAGTCCAGCAGAGGTCGGTG-GAATTGGTGGTG-TAGCGGTGAAATGCACAGATATCACCAAGAACACCCGTGGCGAAGGCGGCCGACTGGTCTGGCACTGACGCTGAGGCAC-GAAAGCGTGGGTAGCGAACAGGATTAGATACCCTTGTAGTC
M04428:485:000000000-J6G24:1:1104:18686:13019 1:N:0:TAAGGCGA+GAGCCTTA/M04428:485:000000000-J6G24:1:1104:18686:13019 2:N:0:TAAGGCGA+GAGCCTTA	k__Bacteria, p__TM7, c__TM7-1, o__, f__, g__, s__	CCTACGGGTGGCAGCAGTAGGGAATTTTTCACAATGGAC-GAAAGTCTGATGGAGCAAC-GCCGCGTGCAGGATGAAGGCCTTAGGGTTGTAAACTGCTTTTGTATATGAAGAATATGACGGTAGTATACGAA-TAAGGATCGGCTAACTCCGTGCCAG-CAGCCGCGGTCATACGGAGGATCCAAGCGTTATCCGGAATTACTGGGCGTAAAGAGTTGCGTAGGCGGCACTGTGTGTCGA-TAGTGAAATCGTACGGCTCAAC-CGTATTAACATTATCGAAACTGCAGAGCTAGAGGACGAGAGAGGTTACTGGAATTCCTTGTGTAGGAGTGAAATCCGTAGA-TATAAGGAGGAACAC-CAATGGCGTAGGCAGGTAACTGGCTCGTTCCTGACGCTCAGGCACGAAAGCATGGGTAGCAAACGGGATTAGATACCCCAG-TAGTC
M04428:485:000000000-J6G24:1:1105:23489:17369 1:N:0:TAAGGCGA+GAGCCTTA/M04428:485:000000000-	k__Bacteria, p__Bacteroidetes, c__[Rhodothermi], o__[Rhodothermales], f__[Balneolaceae], g__, s__	CCTACGGGGGGCAGCAGTGAGGAATCTTGCGCAATGGAC-GAAAGTCTGACGCAGCCAC-GCCGCGTGCCGGAAGAAGGCCCTATGGGTTGTAAACGGCTTTTACCGGGGAAGAACATGTCGGATTTGTCCGGCACTGAC-GGTACCCGGGGAATAAGCAC-CGGCTAACTCCGTGCCAGCAGCCGCGGTAATACGGAGGGTGCAAGCGTTGTCCGGAATCATTGGGTGTAAAGGGTGCG-

J6G24:1:1105:234<br>89:17369<br>2:N:0:TAAGGC<br>GA+GAGCCTT<br>A		TAGGCGGGCTGTTAC-<br>GTCCGGGGTGAAATCTTGCCGCTCAACGGCAAACGGGCCTT<br>GGATACGGGCAGTCTTGAGTATGAGAGAGGCCGGTG-<br>GAATTCGTGGTG-<br>TAGCGGTGAAATGCATAGATATCACGAAGAACACCCGTGG<br>CGAAGGCGGCCGGCTGGCTCATAACTGACGCTGAGGCAC-<br>GAGAGCGTGGGGAGCGAACAG-<br>GATTAGATACCCGAGTAGTC
M04428:485:0000<br>00000-<br>J6G24:1:1115:960<br>3:12054<br>1:N:0:TAAGGC<br>GA+GAGCCTT<br>A/M04428:485:00<br>0000000-<br>J6G24:1:1115:960<br>3:12054<br>2:N:0:TAAGGC<br>GA+GAGCCTT<br>A	k__Bacteria, p__Firmicutes, c__Clostridia, o__Clostridiales, f__, g__, s__	CCTACGGGTGGCAGCAGTCGGGAATTTTGGACAATGGGG-<br>GAAACCCTGATCCAGCGAC-<br>GCCGCGTGAGCGATGAAGGCCTTCGGGTTGTAAAGCTCTGT<br>CTTGAGGGACGAAGGCCAAGGGGAGGAAATGCCCTTT-<br>GGATGACGGTACCTCAAGAGGAA-<br>GCCCCGGCTAACTACGTGCCAGCAGCCGCGGTAATACGTAG<br>GGGGCAAGCGTTGTCCGGAATGACTGGGCG-<br>TAAAGGGCGTCTAGGCGGCCTT-<br>GTAAGTCGCGCGTGAAAGGCCACGGCTCAACCGTGGAGGT<br>GCGTGGGAAACTGCGAGGCTTGAGGGCAGGAGAGGAAA-<br>GCGGAATTCCTGGTGTAGCGGTG-<br>GAATGCGTAGATATCAGGAAGAACACCAGTGGCGAAGGCG<br>GCTTTCTGGACTGTACCTGACGCTGAAGCGCGAAA-<br>GCGTGGGGAGCGAACGGGATTAGATACCCGAGTAGTC
M04428:485:0000<br>00000-<br>J6G24:1:2102:210<br>99:9848<br>1:N:0:TAAGGC<br>GA+GAGCCTT<br>A/M04428:485:00<br>0000000-<br>J6G24:1:2102:210<br>99:9848<br>2:N:0:TAAGGC<br>GA+GAGCCTT<br>A	N/A	CCTACGGGCGGCAGCAGTGAGGAATATTGGACAATGAAC-<br>GAAAGTTTGATCCAGCAA-<br>TATCGCGTGAGTGATACTTTTTGAACTTAGCTATATTTTTATA<br>TTTAAGAGAAAATTAAATTCGTAAAGCTCTTTCAATTTT-<br>GATGAAGTAGACAG-<br>TATTTTTAAAAGAAGTCCCGACTAACTTCGTGCCAGCAGTC<br>GCGGTAAAACGGAGGGGGCAAGCGTTATTCACCTT-<br>GACTGGGCGTAAAGGGTAC-<br>GTAGGCTGTTTTATATTTTTGAAACAAGTAATCTAAGCTTAA<br>CTTTGAAGTTGTTTTAAAAACAATAAGACTTGAGGTTATAA-<br>GAAGTAAGTTACATTTTTAG-<br>TGTAAAGGTGAAATTTTCAAATATTAAAAAGATATCTTGAT<br>GGCGAAAGCTACTTACTAGGTTAAACCTGAC-<br>GCTGAGGTACGAAAGCATGGGGATCAAACAG-<br>GATTAGATACCCGAGTAGTC
M04428:485:0000<br>00000-<br>J6G24:1:2104:156<br>47:19154<br>1:N:0:TAAGGC<br>GA+GAGCCTT<br>A/M04428:485:00<br>0000000-<br>J6G24:1:2104:156<br>47:19154<br>2:N:0:TAAGGC<br>GA+GAGCCTT<br>A	k__Bacteria, p__Planctomycetes, c__Planctomycetia, o__Plancto-mycetales, f__Planctomyceta-ceae, g__Planctomyces, s__	CCTACGGGCGGCTGCAGTCGAGAATCTTCGG-<br>CAATGGGCGAAAGCCTGACCGAGCGAC-<br>GCCACGTGCGGGATGACGGCCTTCGGGTTGTAAACCGCTGT<br>CGAGGGGGATGAAATCACTCAGGGCTATCCCTGGGTGTT-<br>GACAGAGCCCTGGAGGAA-<br>GCGCGGGCTAAGTACGTGCCAGCAGCCGCGGTAATACGTA<br>CTGCGCGAACGTTATTCGGAATCAC-<br>TGGGCTTAAAGGGTGCGTAGGCGGCTTATCAA-<br>GCCCGTGGTGAAAGGTCCCGGCCCAACCGGGGACGTGCTTC<br>GGGGACTGATAGGCTTGGGCGATCTAGGGGTCTGTG-<br>GAACTCCCGGTG-<br>GAGCGGTGAAATGTGTTGAGATCGGGAGGAACGCCGGTGG

	CGAAAGCGACAGACTGGGGATTGGCCGACGCTGAGGCAC-GAAAGCTAGGGTAGCGAACGG-GATTAGATACCCGTGTAGTC

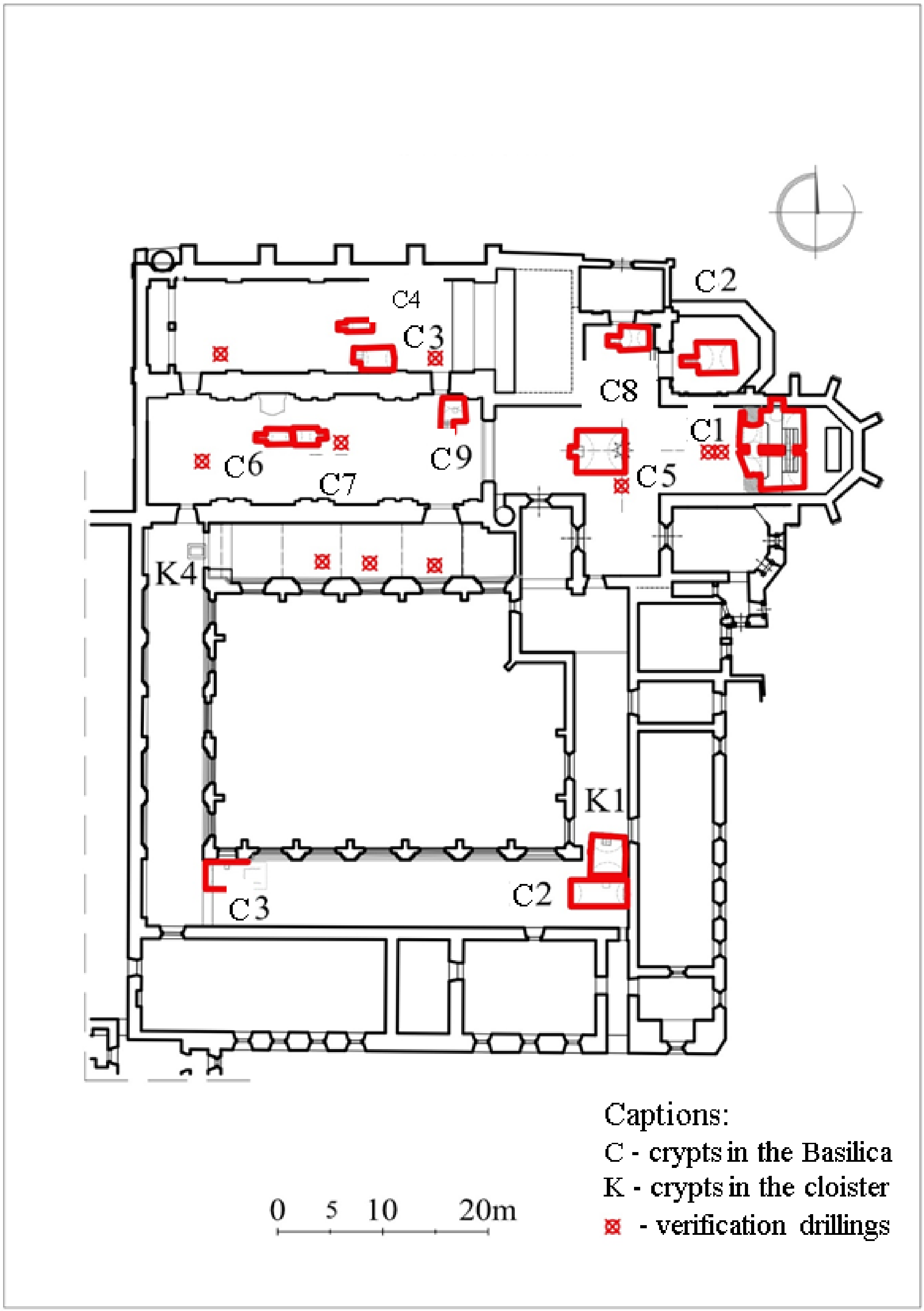

Figure S1. Schematic plan of the Basilica St. Francis of Assini in Cracow and location of the crypts in the church and in the cloister.

Sample code		Description
C2/b4-1	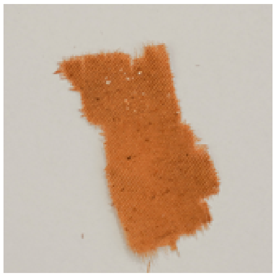	Satin children's dress made for the grave. The front part is formed of two pieces of fabric. There is no part covering the back. (18th century)
C2/b4-2	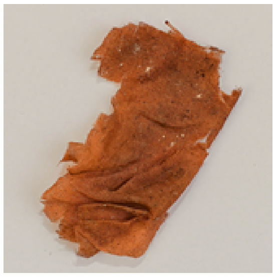	Pillowcase made of the same satin as the garment C2/b4-1. A rectangular piece of fabric was folded in half and sewn along one edge with a straight stitch.
C3/b7	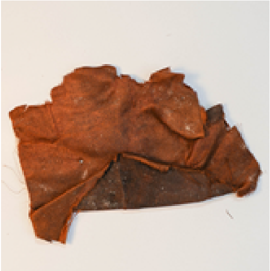	Zhupan, male national costume, made of satin, fastened with 15 buttons. Long sleeves with a wide cuff in the shape of a dog's ear.
C5/b5-1	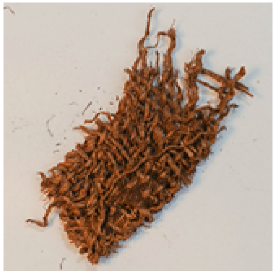	Silk textile sampled from upper part of grave cloth.
C5/b5-2	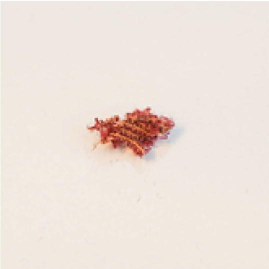	Woollen cloth taken from the hems of the same grave fabric.

Sample code		Description
C5/b9-1	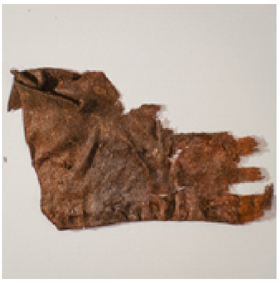	Dress worn during the lifetime made of satin. Tight bodice combined with a wide, wrinkled skirt. Wide, oval neckline, wide sleeves reaching the elbow. (17th century)
C5/b9-2	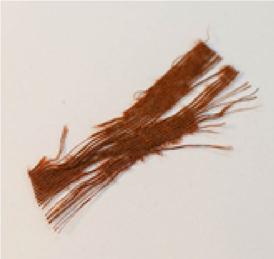	Silk cap worn during life, made of two parts of the same shape, connected at the back with a stitch behind the needle. (17th century)
C5/b11	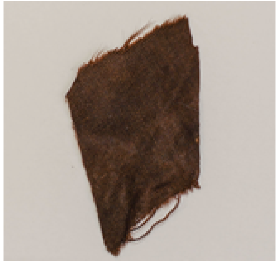	Women's dress worn during the lifetime, made of silk rep, long, a tight-fitting bodice fastened with buttons connected to a wide skirt with a slit at the front. (17-18th century)
C5/b14	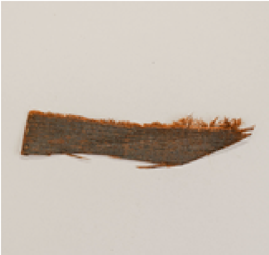	Zhupan, national men's costume, long, reaching to the knee, open, fastened with 23 buttons made of braided string. Long sleeves with a trapezoidal wristband.
C5/b19	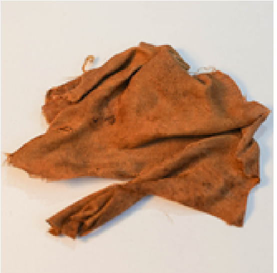	Zhupan, a national men's costume, long, reaching to the knees, fastened with 16 buttons. Very long sleeves without cuffs.

Sample code		Description
C5/b25	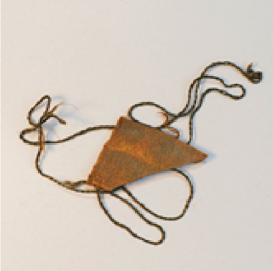	Chekhman, Polish national costume. Fastened up to the waist with buttons threaded through loops. Long sleeves with hooks.
C6/b1	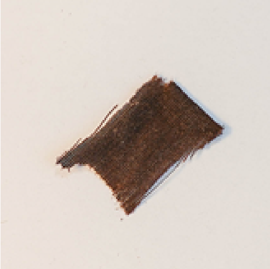	Women's cap made of one rectangular piece of fabric, straight sides, back wrinkled with numerous small pleats, edges finished with silk lace.
C6/b2	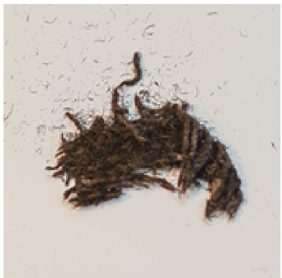	Fabric that covers the coffin from the outside. The fabric was nailed to the boards with brass nails.
C6/b5	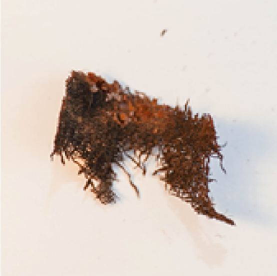	Fragment of an undefined grave cloth.
C7/b2	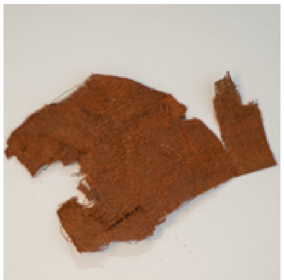	Zhupan, men's national costume, made of silk satin, fastened with 24 buttons made of braided string at the front.

Sample code		Description
C7/b4-1	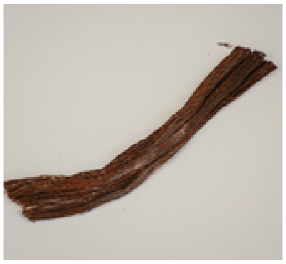	An openwork belt tied several times around a short Zhupan with decorative ends of twisted strings. (17th century)
C7/b4-2	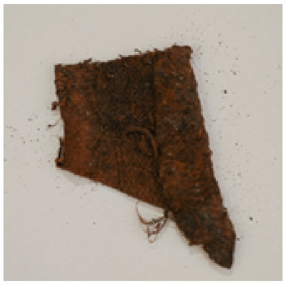	Lining of the short Zhupan.
C8/b6	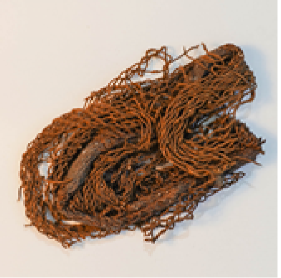	Kontush belt, the traditional men's robe. (17th century)
C8/b7	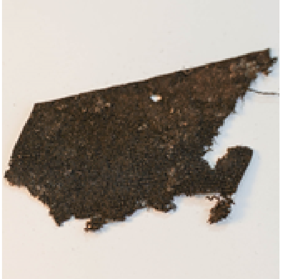	Adult woman's gown, specially made for the grave, made from a single piece of fabric, without a back covering. (18th century)
C9/b1-1	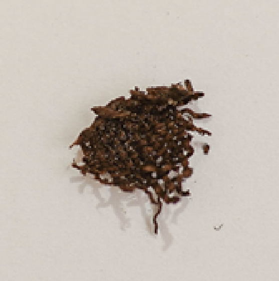	Silk textile sampled from chasuble found in the burial of the priest.

Sample code		Description
C9/b1-2	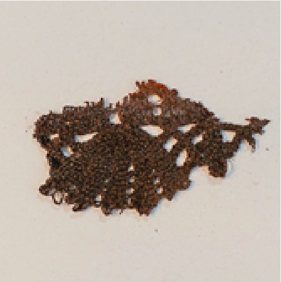	Silk cloth of patch sewn onto a damaged chasuble.
C9/b1-3	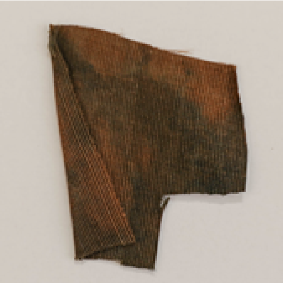	Silk damask from a fragment of a chasuble.
C9/b7	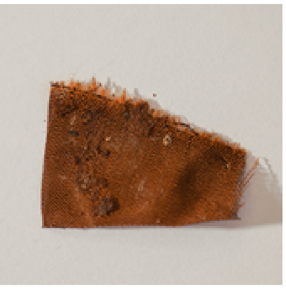	Coffin upholstery fabric.

Figure S2. Images and description of the burial textile samples.

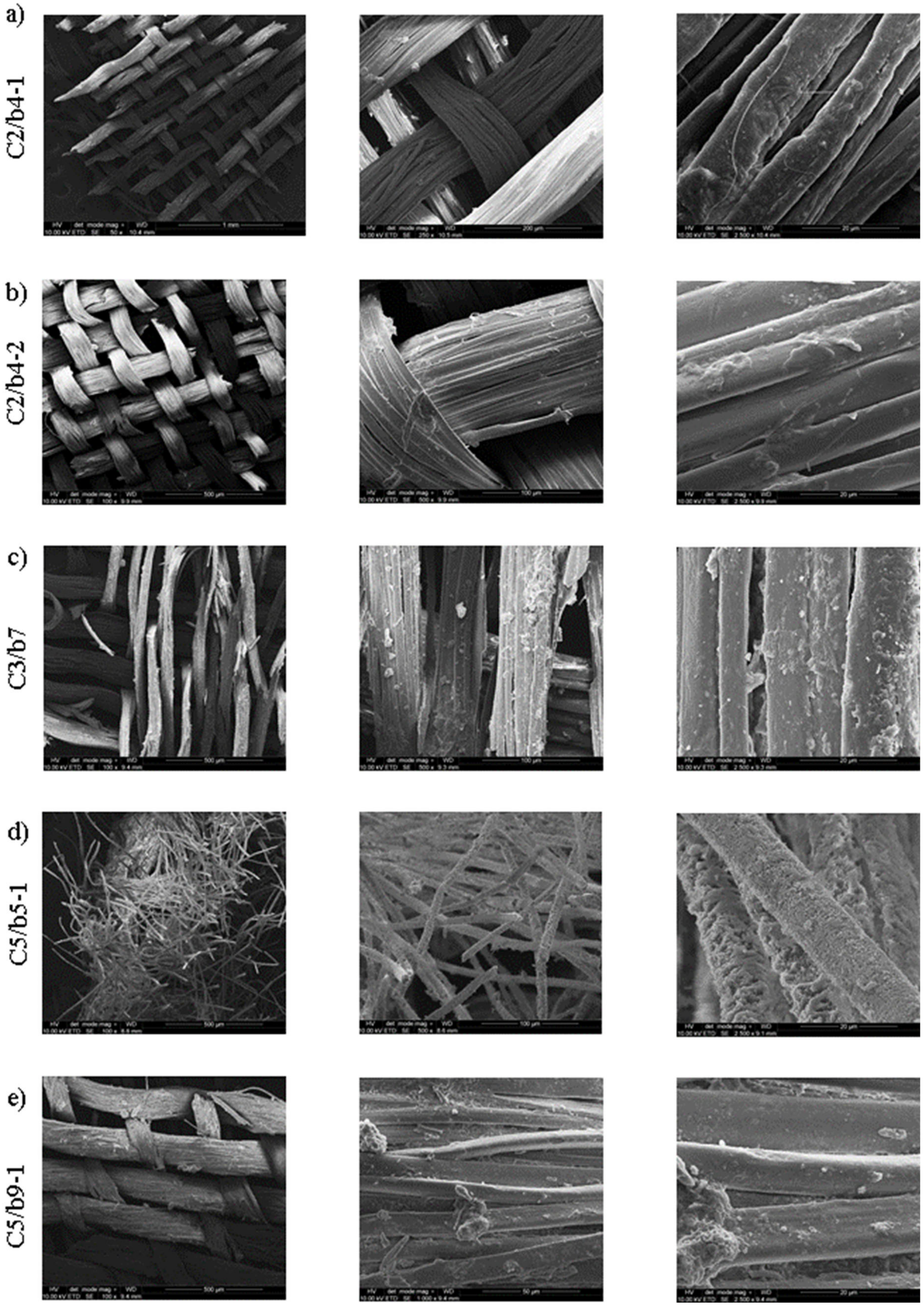
a)
C2/b4-1
b)
C2/b4-2
c)
C3/b7
d)
C5/b5-1
e)
C5/b9-1

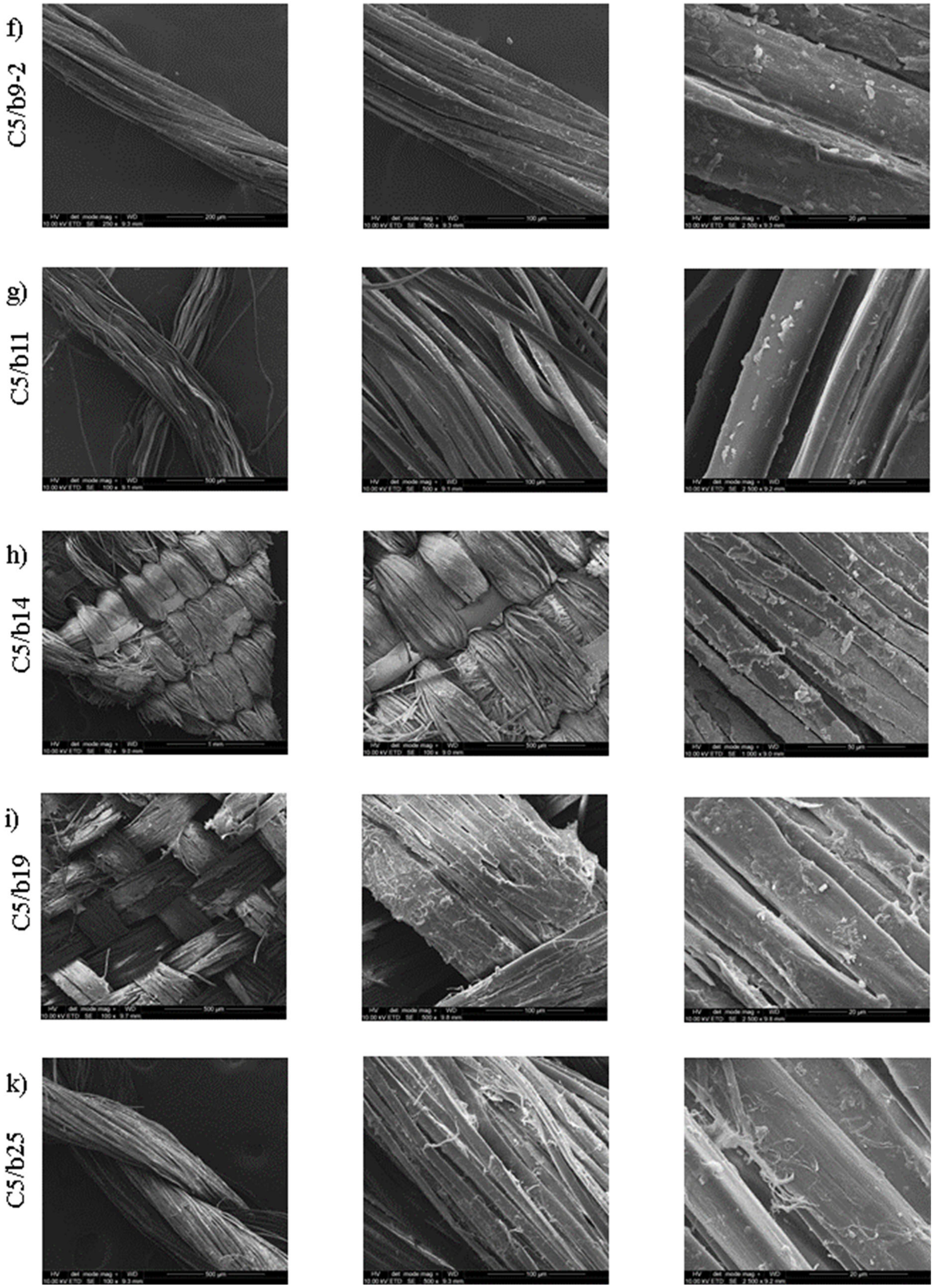
f)
C5/b9-2
g)
C5/b11
h)
C5/b14
i)
C5/b19
k)
C5/b25

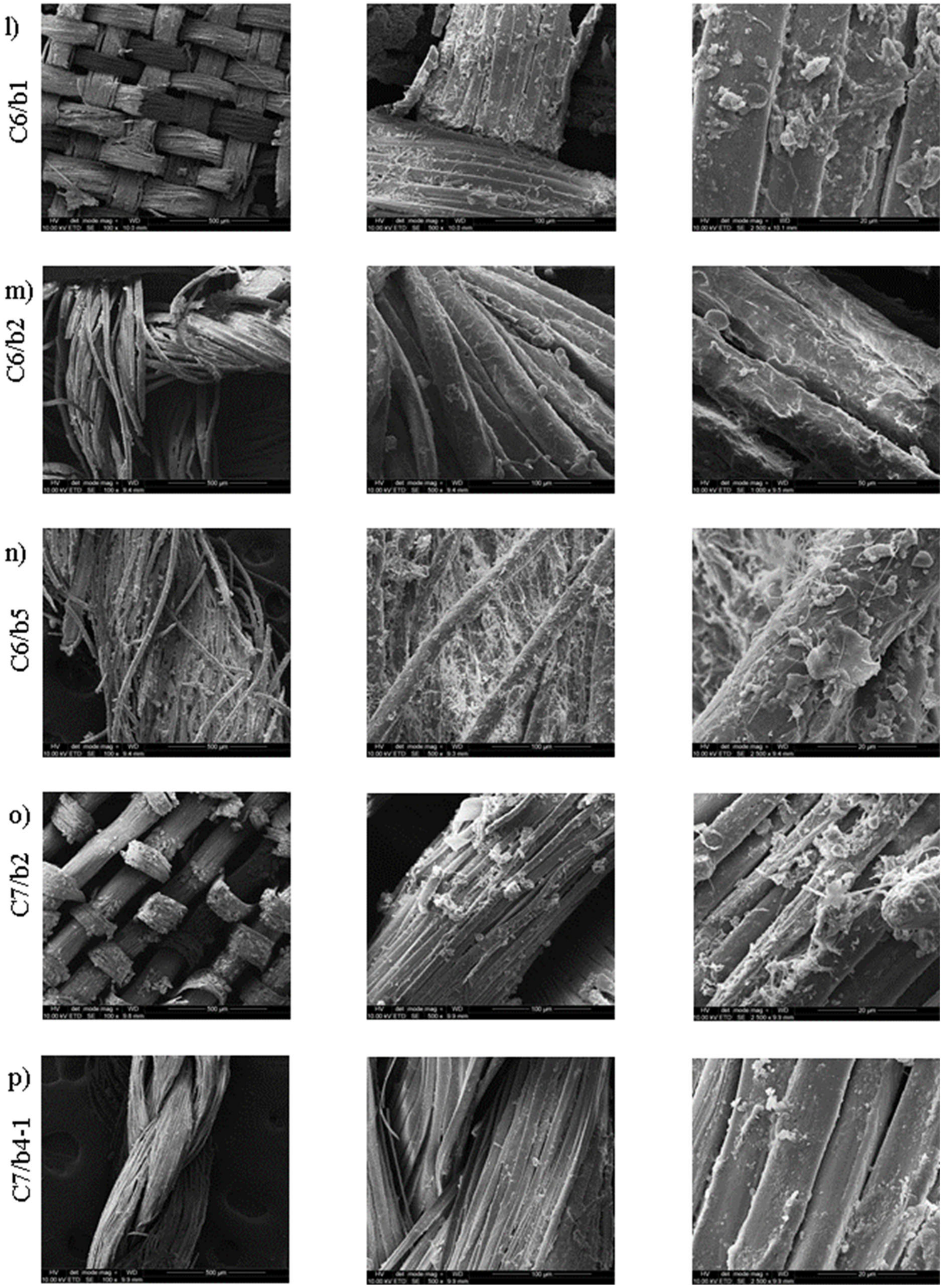
l)
C6/b1
m)
C6/b2
n)
C6/b5
o)
C7/b2
p)
C7/b4-1

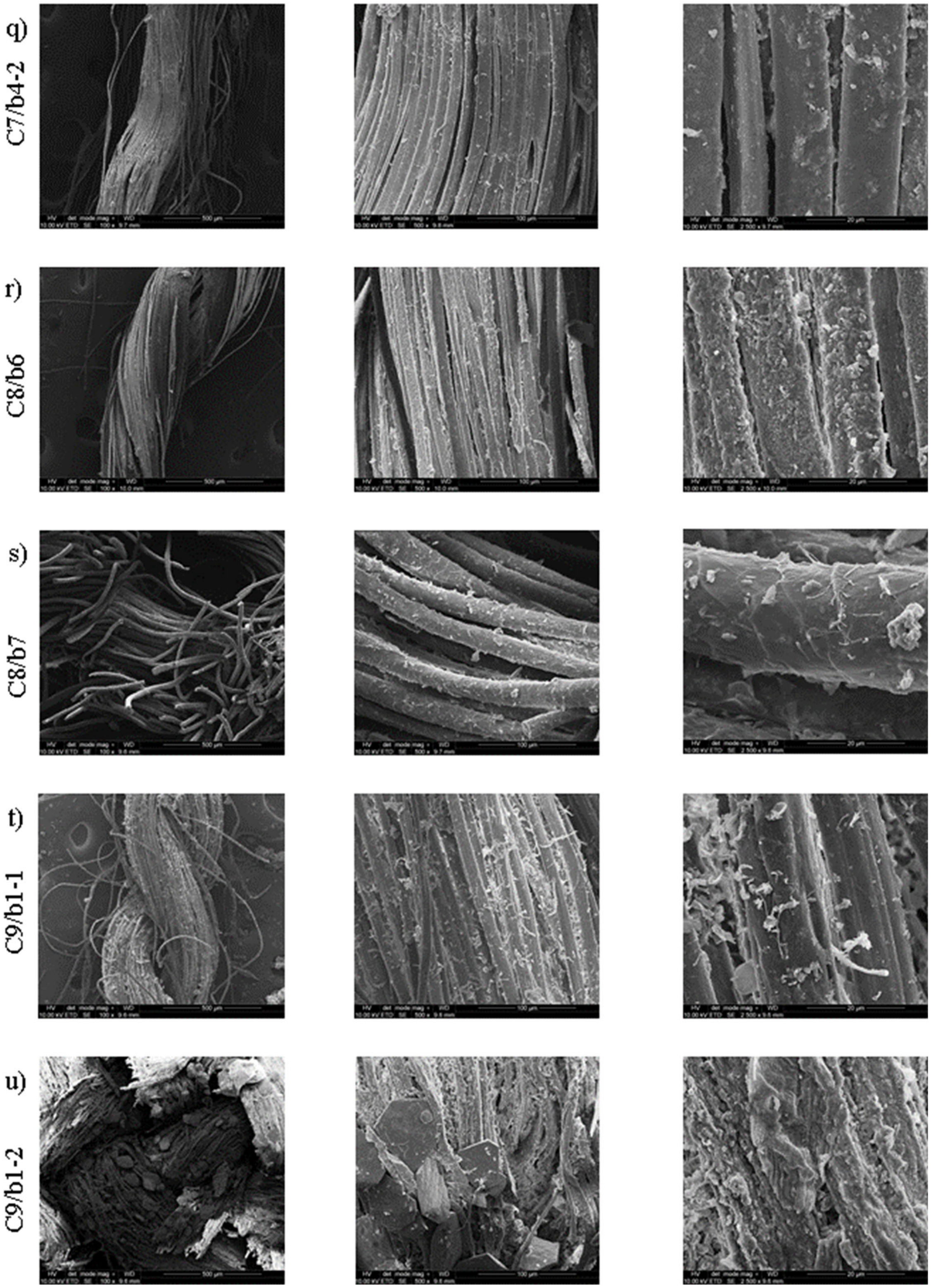
q) C7/b4-2
r) C8/b6
s) C8/b7
t) C9/b1-1
u) C9/b1-2

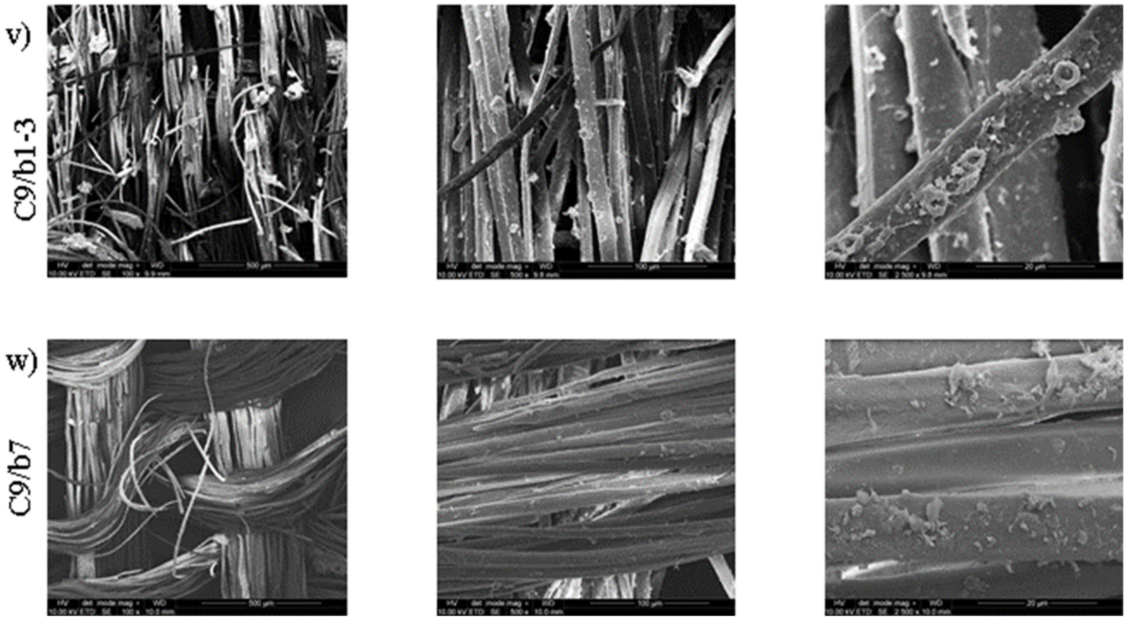

Figure S3. Compilation of SEM images of a investigated threads at a magnification of 100×, 500× and 2500×.

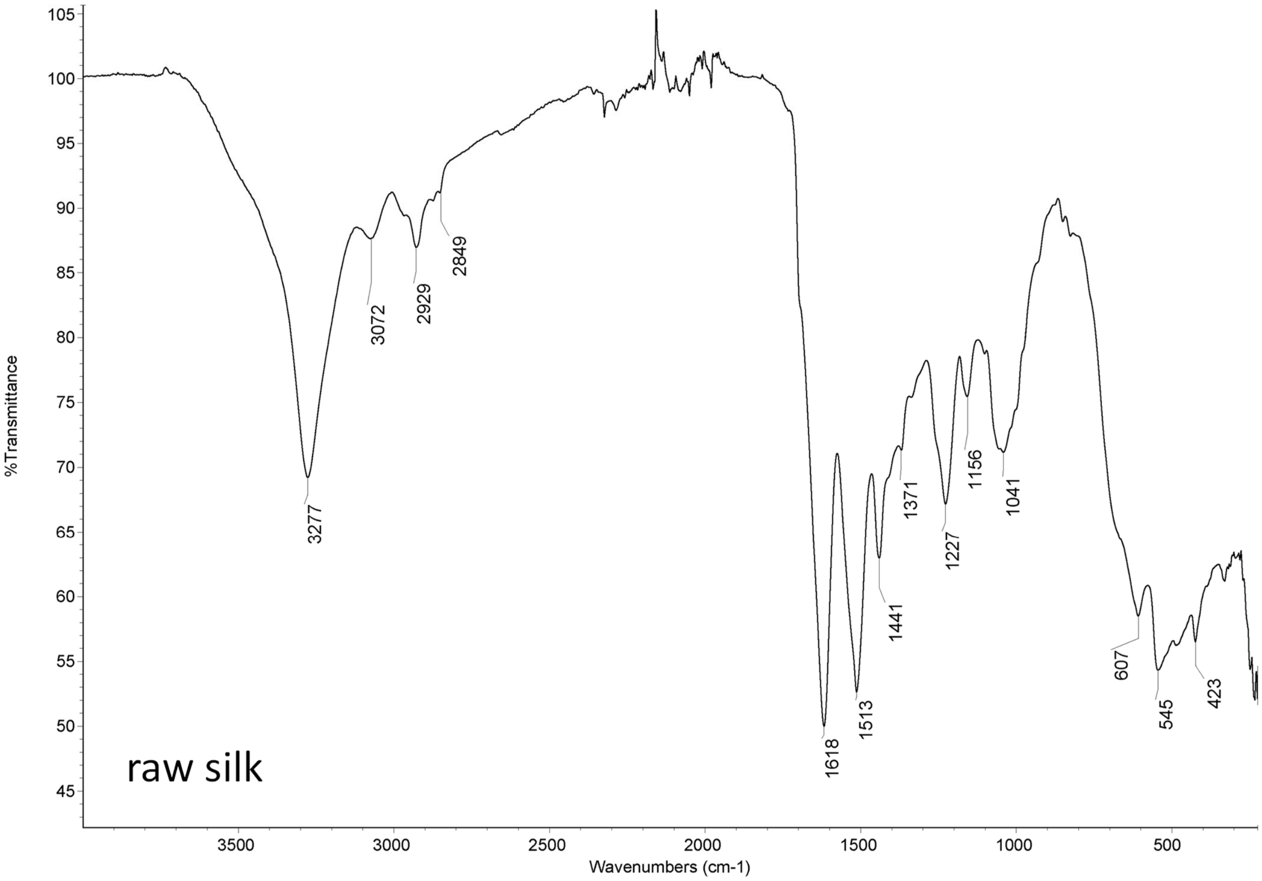
raw silk
%Transmittance
Wavenumbers (cm-1)
3277
3072
2929
2849
1618
1513
1441
1371
1227
1156
1041
607
545
423

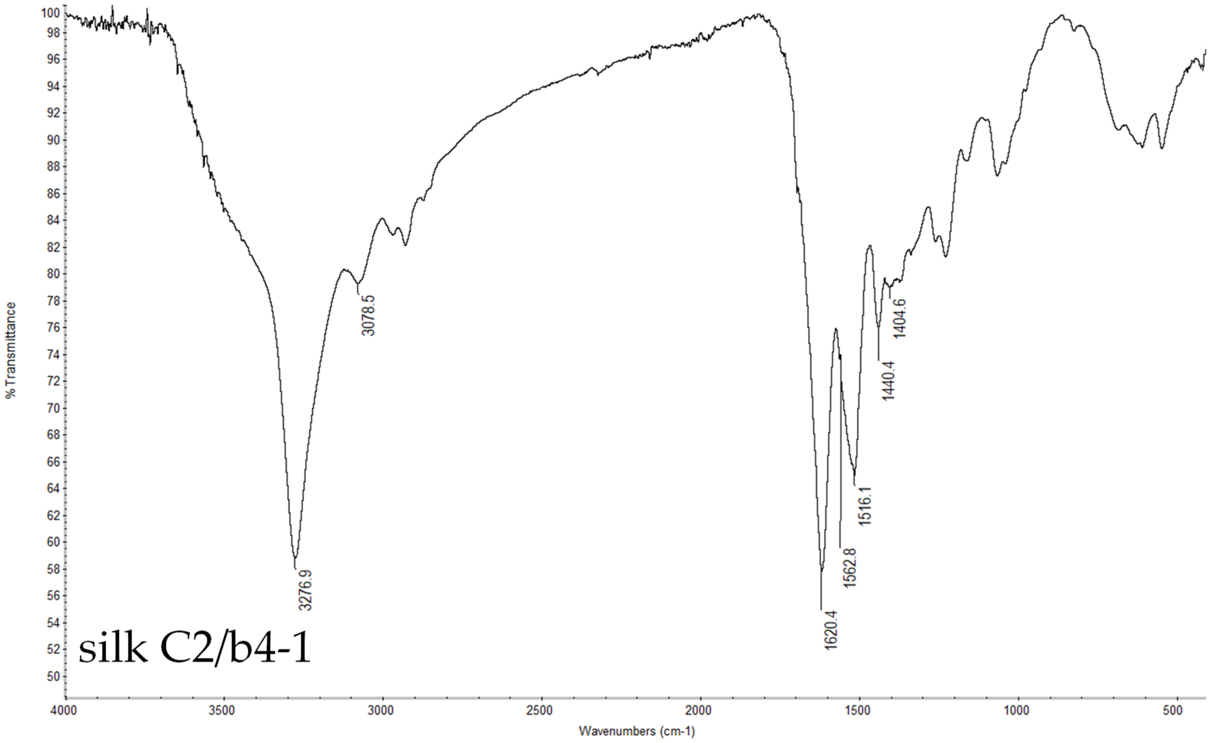
silk C2/b4-1
%Transmittance
Wavenumbers (cm-1)
3276.9
3078.5
1620.4
1562.8
1516.1
1440.4
1404.6

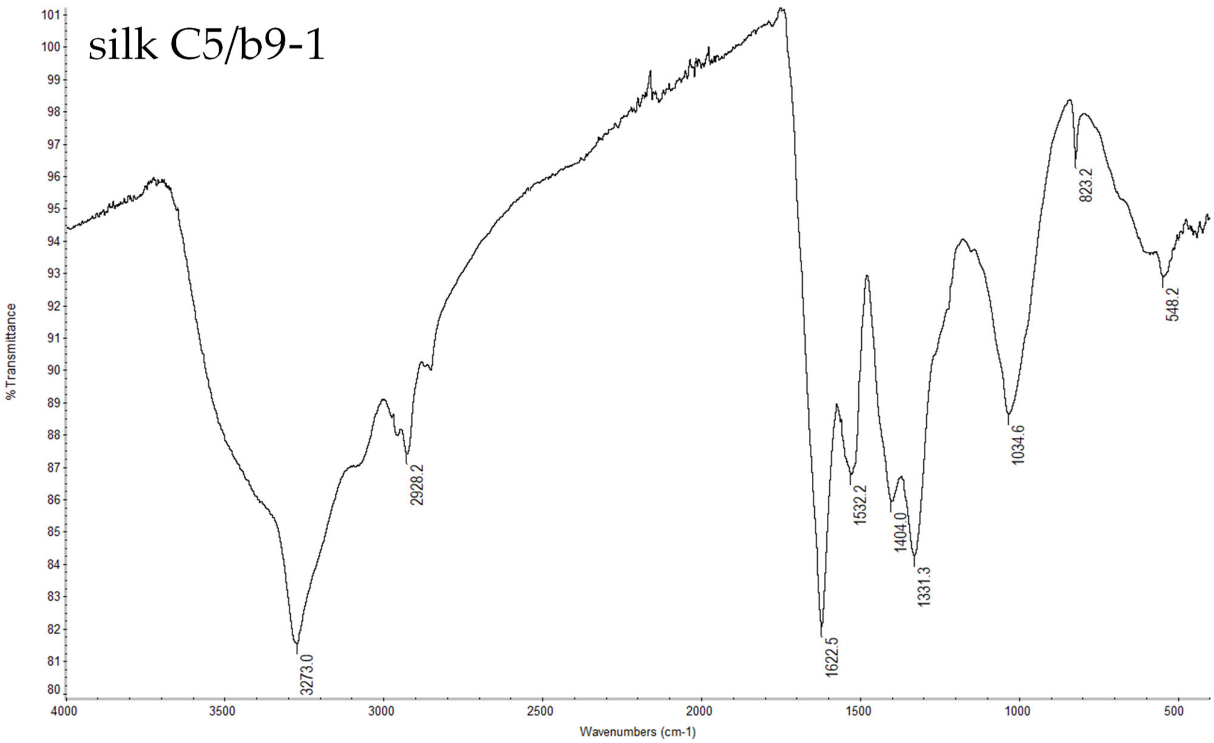
silk C5/b9-1
% Transmittance
Wavenumbers (cm-1)
3273.0
2928.2
1622.5
1532.2
1404.0
1331.3
1034.6
823.2
548.2

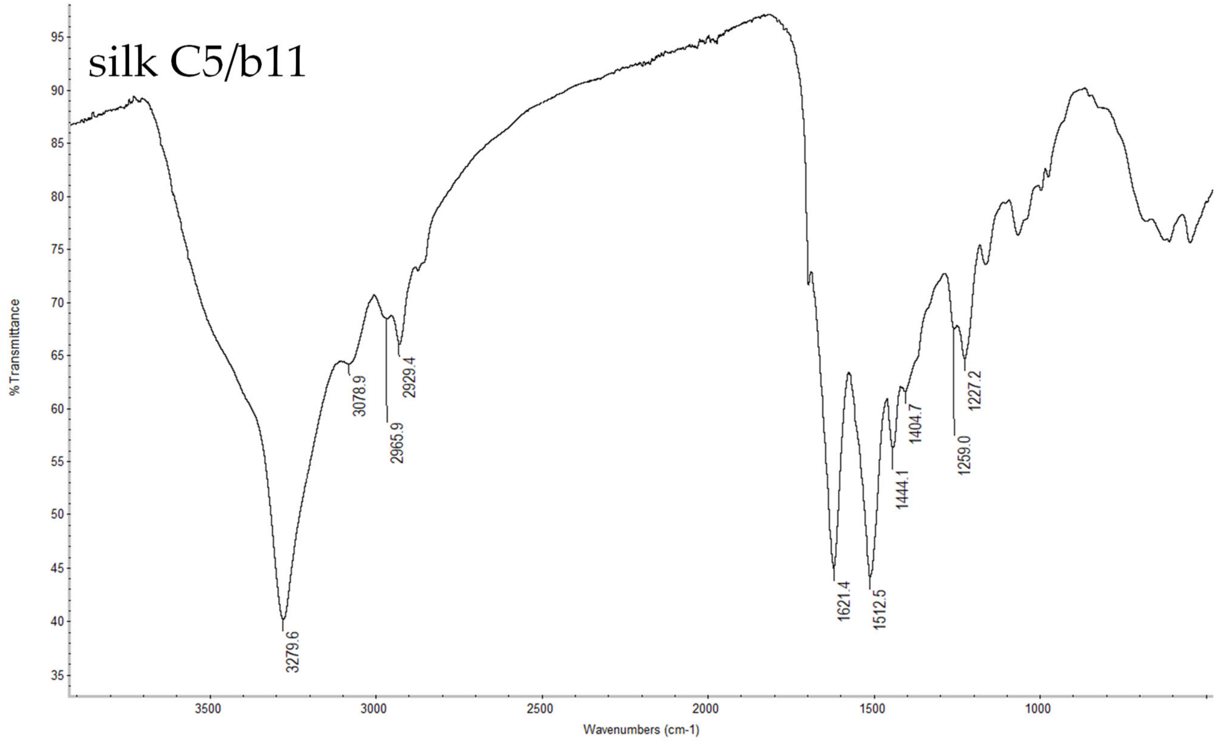
silk C5/b11
% Transmittance
Wavenumbers (cm-1)
3279.6
3078.9
2965.9
2929.4
1621.4
1512.5
1444.1
1404.7
1259.0
1227.2

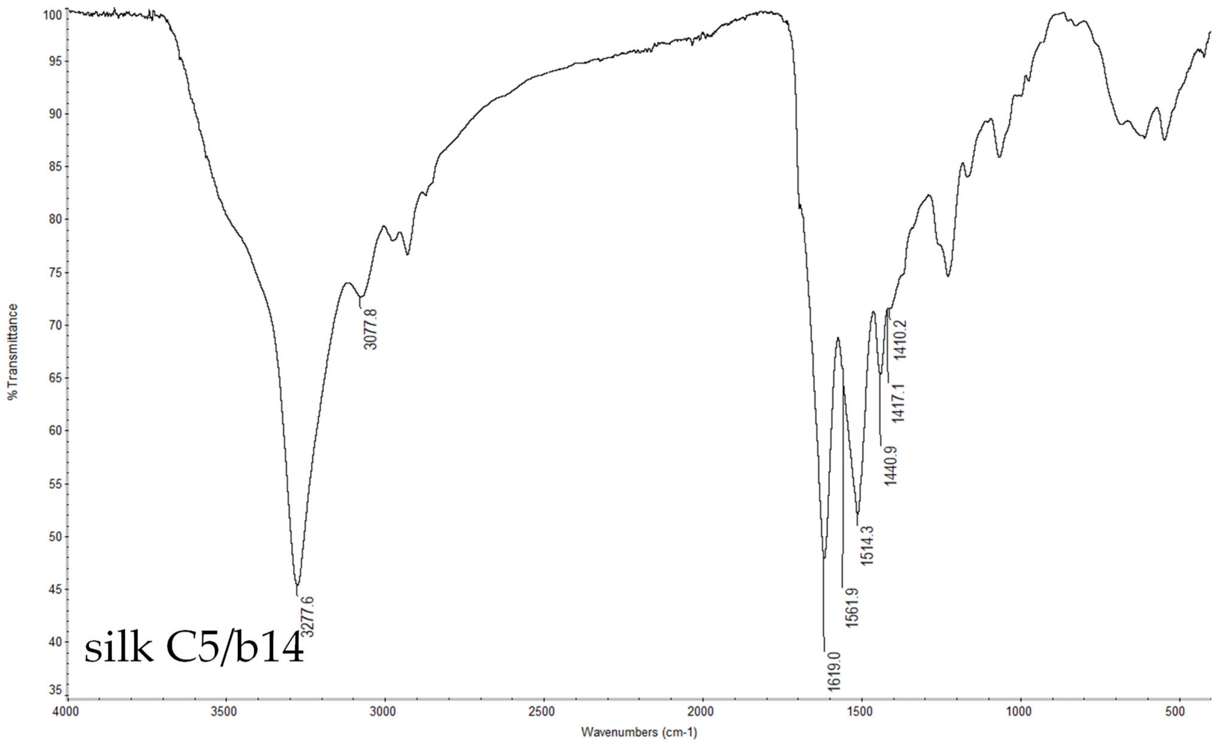
% Transmittance
Wavenumbers (cm-1)
silk C5/b14
3277.6
3077.8
1619.0
1561.9
1514.3
1440.9
1417.1
1410.2

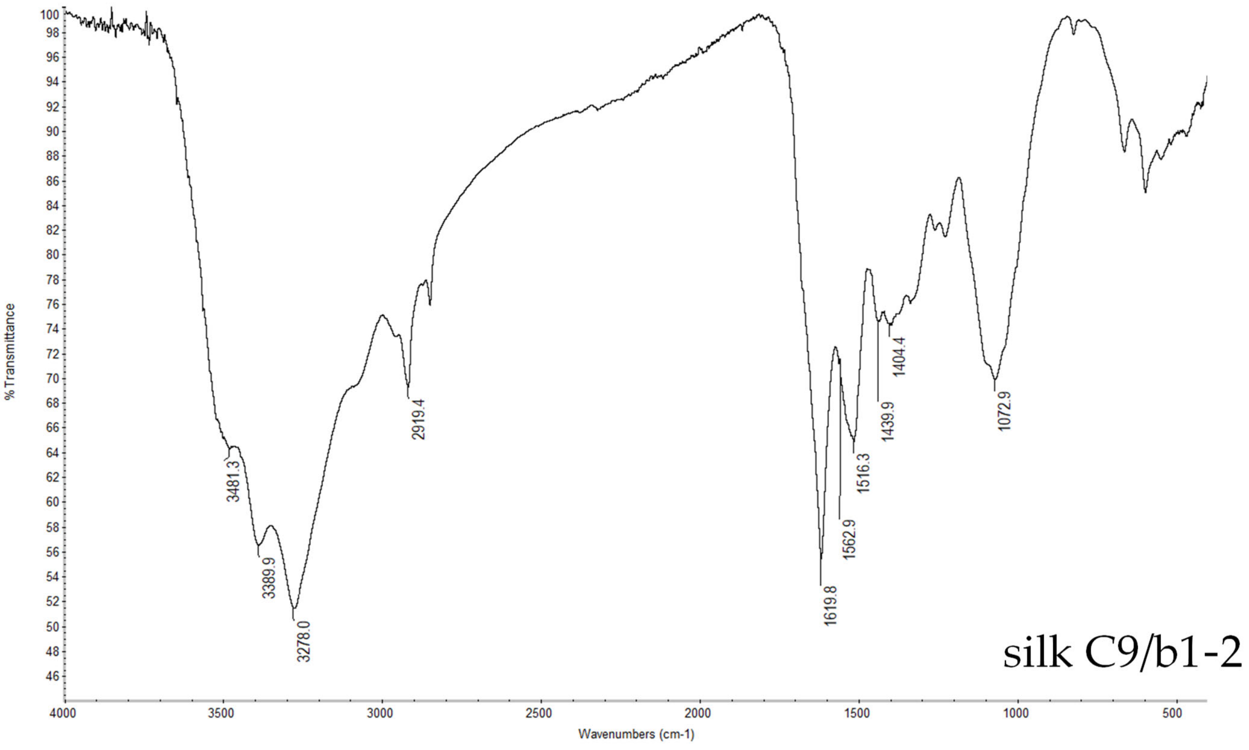
% Transmittance
Wavenumbers (cm-1)
silk C9/b1-2
3481.3
3389.9
3278.0
2919.4
1619.8
1562.9
1516.3
1439.9
1404.4
1072.9

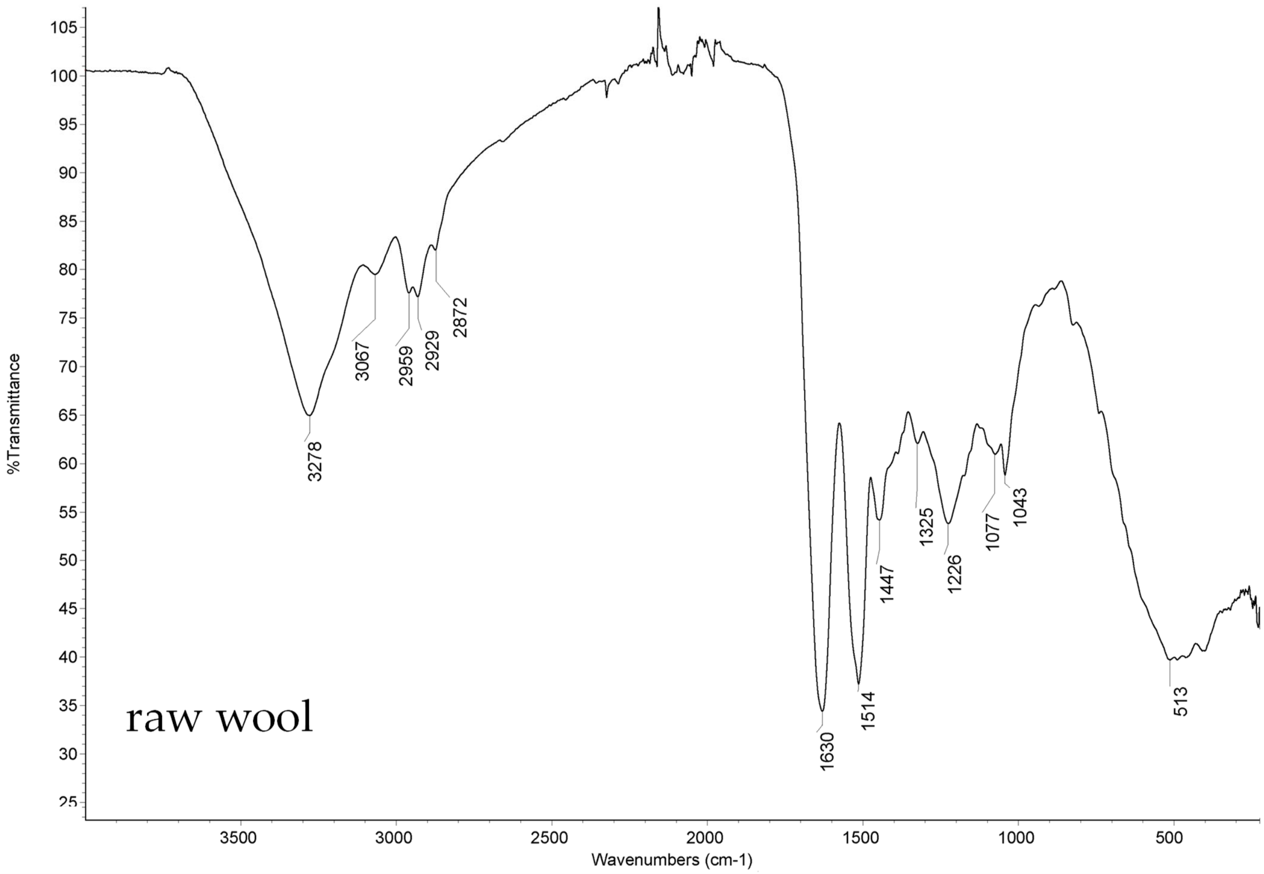
raw wool
%Transmittance
Wavenumbers (cm-1)
3278
3067
2959
2929
2872
1630
1514
1447
1325
1226
1077
1043
513

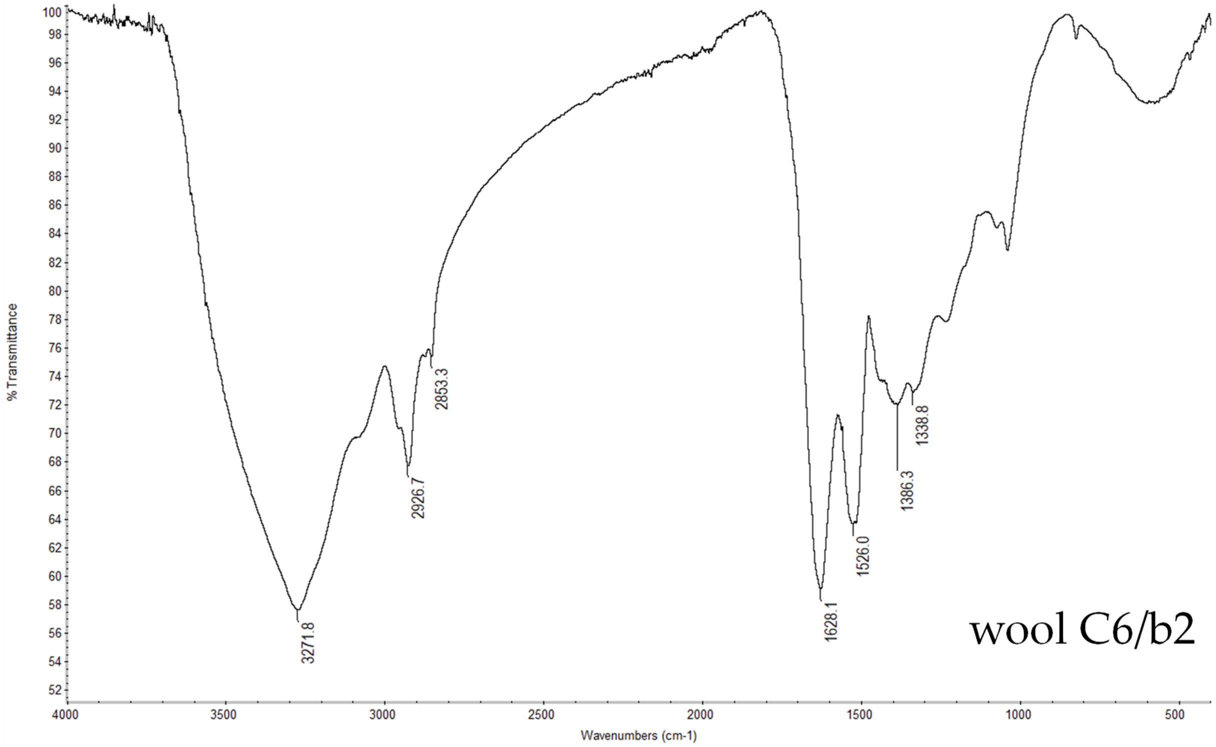
wool C6/b2
% Transmittance
Wavenumbers (cm-1)
3271.8
2926.7
2853.3
1628.1
1526.0
1386.3
1338.8

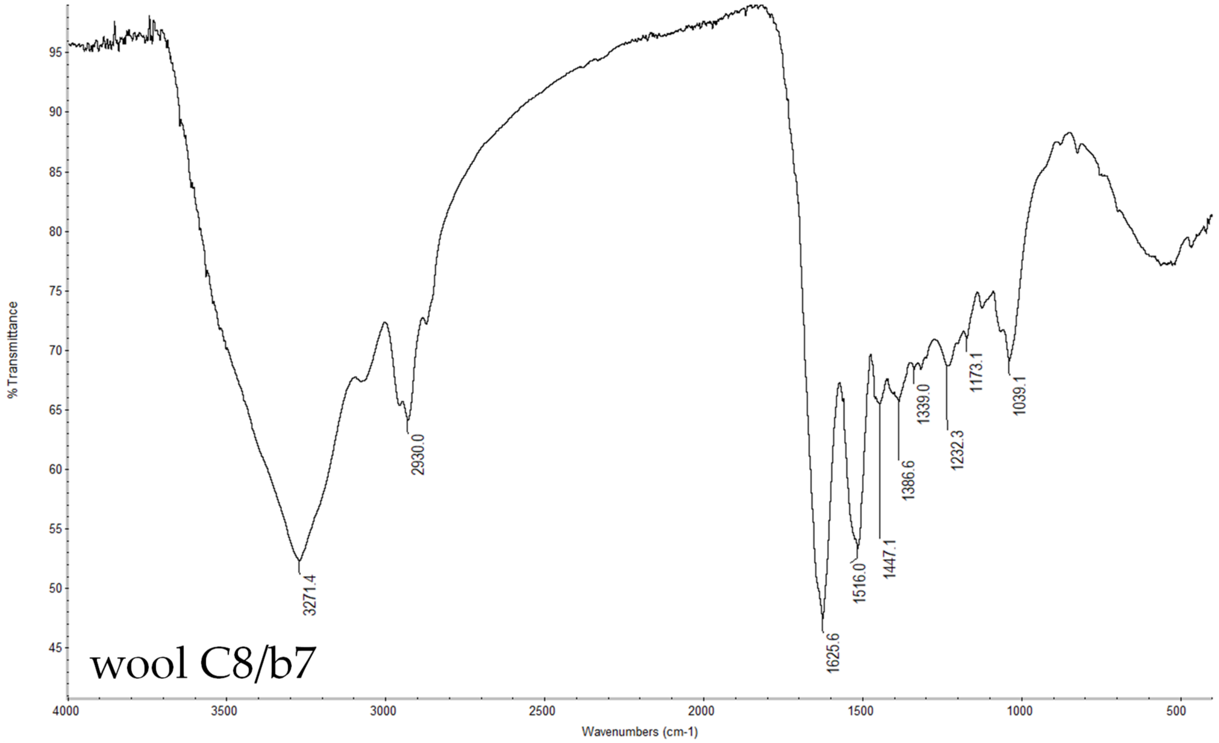

Figure S4. FT-IR spectra of the selected threads.

Munjistin (m/z 283) and rubiadin (m/z 253), the minor anthraquinone components in madder, were identified based on the presence of characteristic daughter ions formed by the further loss of CO, CO_2, or both of these molecules together.

Carminic acid (m/z 491), loses carbon dioxide forming fragment ion at m/z 447. The C-glucoside moiety of carminic acid decomposes via cross-ring cleavages (losses of 90, 120, and 148 Da) resulting in formation of ions at m/z 357 $[^{0.3}X–H–CO_2]^-$, 327 $[^{0.2}X–H–CO_2]^-$, and 299 $[^{0.1}X–H–CO_2]^-$.

Flavokermesic acid (m/z 313), kermesic acid 7-C-α-glucofuranoside (dc IV), kermesic acid 7-C-β-glucofuranoside (dc VII), and 5-aminokermesic acid (m/z 490) are the minor colorants present in American and Polish cochineals [49, 50]. In the spectrum of kermesic acid, fragment ions $[M–H–CO_2]^-$ at m/z 285, $[M–H–CO_2–CO]^-$ at m/z 257, $[M–H–2CO_2–CO]^-$ at m/z 213, $[M–H–2CO_2–2CO]^-$ at m/z 185, and $[M–H–3CO_2–CO]^-$ at m/z 169 is observed. Flavokermesic acid anion (m/z 313) formed fragment ions attributed to the subsequent losses of CO_2 (44 Da) and CO (28 Da) registered at m/z 269, 241, and 197, while the first step of its fragmentation leads to the formation of the $[M–H–CO]^-$ ion at m/z 285.

Erythrolaccin ($[M–H]^-$ at m/z 285) is decarboxylated form of kermesic acid with similar fragmentation pathways and the ions observed at m/z 257, 241, 229, and 213 corresponded to $[M–H–CO]^-$, $[M–H–CO_2]^-$, $[M–H–2CO]^-$, and $[M–H–CO_2–CO]^-$, respectively.

Fisetin and sulfuretin are the main dye components in young fustic. Fisetin has a mass peak $[M–H]^-$ at m/z 285 and characteristic product ions of aglycone RDA fragmentation at m/z: 241 $[M–H–CO_2]^-$, 229 $[M–H–2CO]^-$, 149, 135, and 121 respectively. Sulfuretin with a molecular anion at m/z 269 give fragment ions at m/z: 241 $[M–H–CO]^-$, 225 $[M–H–CO_2]^-$, 213 $[M–H–2CO]^-$, 195 $[M–H–2CO–H_2O]^-$.

Protosappanin B (m/z 303 $[M–H]^-$) has two main fragment ions at m/z 243 $[M–H–C_2H_4O_2]^-$ and 231 $[M–H–C_3H_4O_2]^-$ formed by inner cleavage of ring.

Curcumin has the molecular anion peak ($[M–H]^-$ at m/z 367) and daughter ions at m/z 217 $[M–H–C_9H_9O_2]^-$, 173 $[M–H–C_9H_9O_2–CO_2]^-$, 149 $[M–H–C_{12}H_{11}O_4]^-$, and 134 $[M–H–C_{12}H_{11}O_4–CH_3]^{\bullet-}$, respectively

Chryoseriol (m/z at 299) was fragmented by the loss of a $CH_3^{\bullet}$ unit to form an ion at m/z 284 which is characteristic for the methoxy derivatives of flavones, and subsequently by the neutral loss of a CO molecule yielding a product ion at m/z 256.

Rhamnazin was identified based on the molecular anion $[M–H]^{-}$ at m/z 329 and the fragment ions at m/z 314 $[M–H–CH_3]^{\bullet-}$, 301 $[M–H–CO]^{-}$, 299 $[M–H–2CH_3]^{-}$, 286 $[M–H–CO–CH_3]^{\bullet-}$, 271 $[M–H–CO–2CH_3]^{-}$, and 258 $[M–H–CO–CH_3–CO]^{\bullet-}$.
